# Symbiotic efficiency of *Rhizobium leguminosarum* sv. *trifolii* strains originating from the subpolar and temperate climate regions

**DOI:** 10.1038/s41598-024-56988-1

**Published:** 2024-03-15

**Authors:** Monika Janczarek, Marta Kozieł, Paulina Adamczyk, Katarzyna Buczek, Michał Kalita, Anna Gromada, Aleksandra Mordzińska-Rak, Cezary Polakowski, Andrzej Bieganowski

**Affiliations:** 1 Department of Industrial and Environmental Microbiology, Faculty of Biology and Biotechnology, Institute of Biological Sciences, Maria Curie-Skłodowska University, 19 Akademicka, 20-033 Lublin, Poland; 2 Department of Genetics and Microbiology, Faculty of Biology and Biotechnology, Institute of Biological Sciences, Maria Curie-Skłodowska University, 19 Akademicka, 20-033 Lublin, Poland; 3 Department of Biochemistry and Molecular Biology, Faculty of Medical Studies, Medical University in Lublin, 1 Chodźki, 20-093 Lublin, Poland; 4 Department of Natural Environment Biogeochemistry, Institute of Agrophysics, Polish Academy of Sciences, 4 Doświadczalna, 20-290 Lublin, Poland

**Keywords:** Microbiology, Environmental sciences

## Abstract

Red clover (*Trifolium pratense* L.) is a forage legume cultivated worldwide. This plant is capable of establishing a nitrogen-fixing symbiosis with *Rhizobium leguminosarum* symbiovar *trifolii* strains*.* To date, no comparative analysis of the symbiotic properties and heterogeneity of *T. pratense* microsymbionts derived from two distinct geographic regions has been performed. In this study, the symbiotic properties of strains originating from the subpolar and temperate climate zones in a wide range of temperatures (10–25 °C) have been characterized. Our results indicate that all the studied *T. pratense* microsymbionts from two geographic regions were highly efficient in host plant nodulation and nitrogen fixation in a wide range of temperatures. However, some differences between the populations and between the strains within the individual population examined were observed. Based on the *nodC* and *nifH* sequences, the symbiotic diversity of the strains was estimated. In general, 13 alleles for *nodC* and for *nifH* were identified. Moreover, 21 and 61 polymorphic sites in the *nodC* and *nifH* sequences were found, respectively, indicating that the latter gene shows higher heterogeneity than the former one. Among the *nodC* and *nifH* alleles, three genotypes (I–III) were the most frequent, whereas the other alleles (IV–XIII) proved to be unique for the individual strains. Based on the *nodC* and *nifH* allele types, 20 *nodC-nifH* genotypes were identified. Among them, the most frequent were three genotypes marked as A (6 strains), B (5 strains), and C (3 strains). Type A was exclusively found in the temperate strains, whereas types B and C were identified in the subpolar strains. The remaining 17 genotypes were found in single strains. In conclusion, our data indicate that *R. leguminosarum* sv. *trifolii* strains derived from two climatic zones show a high diversity with respect to the symbiotic efficiency and heterogeneity. However, some of the *R. leguminosarum* sv. *trifolii* strains exhibit very good symbiotic potential in the wide range of the temperatures tested; hence, they may be used in the future for improvement of legume crop production.

## Introduction

The Fabaceae is a large plant family that encompasses ~ 22,360 species classified to 770 genera^1–4^. Legumes belonging to this family occur worldwide and many of them are important protein sources for humans and animals and play an essential role in crop rotation by increasing nitrogen (N) levels available to plants^4,5^. Most Fabaceae plants are able to establish N-fixing symbiotic associations with bacteria, collectively called rhizobia^6–14^. This process, referred to as biological nitrogen fixation (BNF), provides essential economic and ecological benefits, since it substantially contributes to agricultural production and the N cycle on the Earth^15^. BNF yields more than 100 million tons of fixed N/year to soil, with ~ 60 million tons of N_2_ fixed biologically by agriculturally cultivated legume crops^12,16–19^. BNF contributes to a significant reduction of the amounts of synthetic N fertilizers applied in agriculture, limiting their adverse impacts on natural ecosystems^16,20^. Therefore, the use of rhizobia in sustainable agriculture reduces the need for synthetic N fertilizers^18,21^.

The term rhizobia refers to a polyphyletic group of Gram-negative bacteria that live in soils saprophytically and, in suitable conditions, enter into an intracellular symbiotic relationship with compatible plant hosts^7,21^. The establishment of a symbiosis requires an exchange of molecular signals originating from both symbiotic partners, with the greatest importance of plant flavonoids and rhizobial lipochitooligosaccharides (also called Nod factors)^18,21^. This “molecular dialogue” leads to formation of special organs on legume roots (or occasionally stems), called nodules, inside which bacteria proliferate and differentiate into bacteroid forms able to reduce N_2_ into ammonia, which is then used by the host^6,21–23^. The symbiotic interactions are characterized by high host specificity, since individual legumes are recognized and infected by only some rhizobial species. Usually, rhizobia have a very narrow host plant range. For example, *R. leguminosarum* sv. *trifolii* strains are dominant microsymbionts of plants from the genus *Trifolium* (*T. pratense, T. repens*, and *T. rubens*), whereas *R. leguminosarum* sv. *viciae* strains establish N-fixing symbiotic interactions with *Pisum*, *Vicia*, *Lens*, and *Lathyrus*^21,24–29^*.* However, some rhizobial species can nodulate many hosts (e.g., *R. gallicum* strains establish symbioses with legumes from the genera *Phaseolus, Caliandra, Gliricidia*, *Sesbania,* and *Piptadenia*)^21,30,31^. Rhizobia are characterized by large genomes (up to 9 Mbp) consisting of one chromosome and a few very large plasmids and chromids of different sizes (from ca. 100 kb to 2 Mb)^32–34^. Among these plasmids, one plasmid, called the symbiotic plasmid (pSym), carries the majority of the genes involved in nodulation (*nod*) and N fixation (*nif* and *fix*)^7,18,21^. However, other plasmids can also carry other copies of these genes (e.g., *fixGHIS* and *NOQP*), as well as, in some strains, the only copy of *fixL*^8,9,34^.

Soil is a challenging environment for bacteria, where the conditions may change rapidly and bacteria have to acclimate and adapt in order to survive. The diversity of strains occupying nodules is a function of their biodiversity in the rhizosphere. To survive as saprophytes and to nodulate, rhizobia have to compete with other bacterial species and with other rhizobial strains, thus competitive traits are very important for nodulation success^21,24,25,35^. Therefore, studies on rhizobial biodiversity are an important approach in finding stress-tolerant native isolates^11,36,37^. As demonstrated in several papers, various environmental factors influence the composition and activity of rhizobial populations in soil and the rhizosphere. Among them, soil pH, drought, heavy metals, and temperature are the major abiotic stress factors^10,20,36–39^. Accordingly, the search for rhizobial isolates with high tolerance to stress conditions may be a way of improving legume yields, especially in more adverse climate and soil conditions. Clovers (*Trifolium* spp.) represent a large genus of the Fabaceae family encompassing ~ 250 different species from different geographical regions (Europe, North and South America, Australia, and Africa)^40–42^. The red clover (*Trifolium pratense* L.) is one of the most widely cultivated forage plants in Europe, North America, and Australia^43,44^. Microsymbionts of clover plants belong to *R. leguminosaum* sv. *trifolii* and they occur in various geographical regions, including those characterized by highly stressful conditions, such as low temperatures in arctic and subarctic climatic zones^45,46^. To give more insight into the influence of low temperature on the genetic diversity of *T. pratense* microsymbionts, we performed comparative analysis of the genetic diversity of strains isolated from root nodules of red clover plants grown in two European regions that essentially differ in temperature conditions (i.e., northern Norway and south-eastern Poland)^46^. In total, 120 strains (60 strains for each geographic region) were genetically characterized. A high degree of heterogeneity was found within the studied populations of clover root isolates. However, a lower genetic diversity of the strains from the subpolar zone than those from the temperate zone was reported, suggesting that a low temperature can negatively influence the genetic diversity of rhizobial strains^46^.

In this study, we intended to check whether the symbiotic potential of clover root isolates originating from different climatic regions differs and how low temperature influences the symbiotic efficiency of these strains with their red clover host. For this analysis, 31 representative strains were selected, which reflected the genetic diversity of populations (i.e., 16 and 15 strains from the temperate and subpolar population, respectively). We examined and compared various symbiotic properties of the clover root isolates originating from the two geographic regions characterized by essentially different temperature conditions.

## Results

### Determination of clover root infection and nodulation effectiveness by *R. leguminosarum* sv. *trifolii* strains

**Figure 1:**
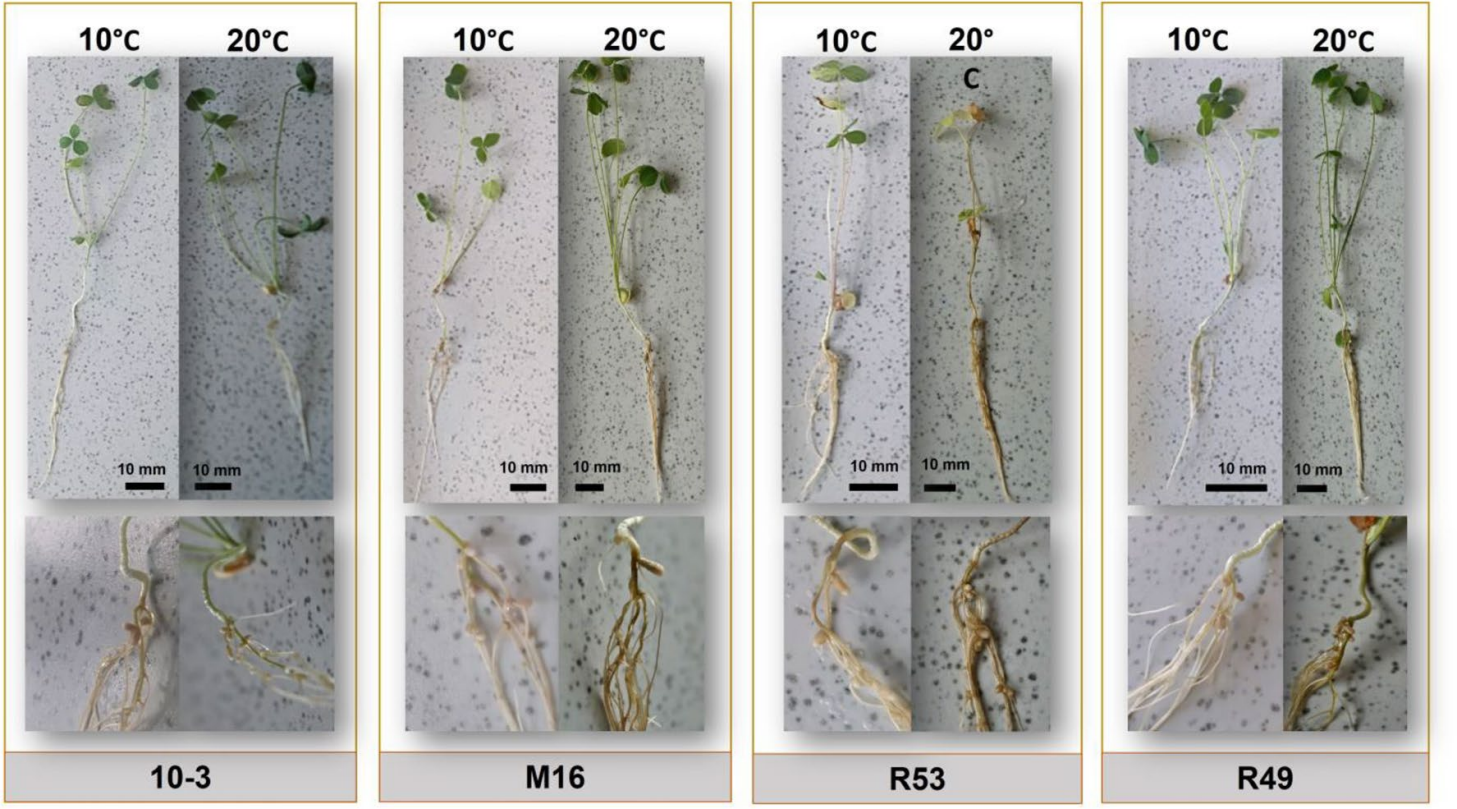
Appearance of 35 days clover plants inoculated with two representative *R. leguminosarum* sv. *trifolii* strains from each climate zone population tested; temperate strains 10-3 and M16 and subpolar strains R52 and R49 grown at low (10 °C) and optimal (20 °C) temperatures, respectively.

To characterize the symbiotic properties of the microsymbionts isolated from the root nodules of red clover plants, we selected 31 representative strains from both climatic populations, reflecting their high genetic diversity (i.e., 16 and 15 representatives of the temperate and subpolar climate population, respectively). For this purpose, 5-week experiments in control conditions using N-free Fåhraeus agar^47^ and *T. pratense* as a host plant were performed. Four temperatures 10, 15, 20, and 25 °C were tested. The clover plants were examined after each 7 days post inoculation (dpi) and the number of nodules subsequently appearing on the roots was counted. The nodules were well visible because of their size and shape and the plant medium clarity. The older nodules had elongate shape and light pink color caused by the presence of leghemoglobin that confirmed nodule functionality (Fig. 1). The effectiveness of host root infection, given as % of the inoculated plants that had root nodules, was the first symbiotic parameter characterized. As shown in Tables S1 and S2 (Supplementary material), all the tested *R. leguminosarum* sv*. trifolii* strains from both populations were highly effective in nodulation of red clover plants in a wide range of temperatures (10–25 °C). At 20–25 °C, which are temperatures optimal for the growth of both symbiotic partners, about 50% of the tested plants had root nodules already after the first week of the experiment (7 dpi) (at 20 °C), and the values were even higher at 25 °C. Moreover, 100% of the tested plants grown at 20 °C and 25 °C had nodules after 28 dpi. We observed that the low temperature significantly affected root infection and nodulation (Tables S1 and S2). With respect to the individual strains examined, temperate strains 4–3, 10–3, M2, M16, M19, and subpolar strains R1, R23, R49, R118, and R137 were the most efficient in this process. The comparison of the effectiveness of clover root infection by the strains from the two climate collections revealed that the differences observed between them were not very high. The subpolar strains exhibited only slightly higher effectiveness of root infection at low temperatures (10 °C and 15 °C) than the temperate strains (Table 1). The highest differences were observed at 14 dpi at 10 °C (1.91-fold, *p* = 0.012) and 15 °C (1.26-fold, *p* ≥ *0.05*) as well as 21 dpi at 15 °C (1.18-fold, *p* = 0.038). All these data indicate that symbiosis of *R. leguminosarum* sv*. trifolii* strains with clover plants was well-adapted to cold stress.

**Table 1:** Effectiveness of clover root infection by *R. leguminosarum* sv*. trifolii* strains examined in a wide range of temperatures.

Origin of population	Strains	Temp. (°C)	% of plants with root nodules observed during the 35-day experiment, calculated for all strains from each climate collection				
			7 dpi	14 dpi	21 dpi	28 dpi	35 dpi
Temperate climate region	2-2, 3-1, 3-3, 4-3, 5-8, 6-11, 8-3, 8-11, 10-3, KW1-9, KW2-9, M2, M14, M16, M19, 24.2	10	0 ± 0^(aA)^	6.8 ± 3.1^(aB)^*	57.8 ± 7.2^(aC)^	83.7 ± 6.3^(aD)^	91.9 ± 8.2^(aD)^
		15	0 ± 0^(aA)^	32.8 ± 5.2^(bB)^	76.6 ± 3.4^(bC)^*	93.7 ± 6.4^(bD)^	97.8 ± 2.2^(bD)^
		20	54.7 ± 9.7^(bA)^	97.5 ± 2.5^(cB)^	99.4 ± 4.4^(cB)^	100 ± 0^(cB)^	100 ± 0^(bB)^
		25	94.1 ± 5.9^(cA)^	100 ± 0^(cA)^	100 ± 0^(cA)^	100 ± 0^(cA)^	100 ± 0^bA)^
Subpolar climate region	R1, R13, R23, R26, R32, R41, R49, R51, R53, R56, R66, R70, R108, R118, R137	10	0 ± 0^(aA)^	13 ± 2.7^(aB)^*	63 ± 7.0^(aC)^	92.3 ± 7.7^(aD)^	98 ± 8.0^(aD)^
		15	0 ± 0^(aA)^	41.3 ± 8.7^(bB)^	90.7 ± 5.7^(bC)^*	97.7 ± 2.3^(aD)^	100 ± 0^(aD)^
		20	54.7 ± 10.2^(bA)^	97 ± 7.0^(cB)^	99.3 ± 4.3^(bB)^	99.7 ± 4.7^(aB)^	100 ± 0^(aB)^
		25	92.5 ± 7.5^(cA)^	99 ± 5.0^(cB)^	99.7 ± 4.7^(bB)^	100 ± 0^(aB)^	100 ± 0^(aB)^

Results are presented as mean ± SD. Lower case letters in brackets indicate statistically significant differences (*p* ≤ 0.05) for each population tested at different temperatures at a particular time point (dpi); upper case letters in brackets indicate statistically significant differences (*p* ≤ 0.05) for each population tested at particular temperatures at different time points (dpi); two-way ANOVA (Tukey’s post hoc test);

* indicates statistically significant differences (*p* ≤ 0.05) between two populations tested at each temperature and time point (dpi).

The second symbiotic parameter tested was the efficiency of nodule induction on clover roots (Tables S3 and S4). The strains from two climatic regions proved to be highly effective in host plant nodulation. Among the temperate strains, 2–2, 3–3, KW1-9, KW2-9, M2, and M19 were the most efficient. This was observed already at 7 dpi and at further time of the experiment (up to 35 dpi) at the optimal temperatures (20 °C and 25 °C) (Table S3). Interestingly, all the tested temperate strains were able to induce nodules on the host roots even at as low temperature as 10 °C. The analysis of the number of nodules formed at 10 °C and 15 °C showed that strains 3–1, KW1-9, KW2-9, M2, M16, M19, and 24.2 exhibited the highest nodulation efficiency (especially at 21 dpi). The highest results were recorded for strain KW1-9 (0.95 nodules per root at 10 °C and 2.65 at 15 °C, respectively) (Table S3). With respect to this symbiotic trait, the subpolar strains were also highly effective and even more effective at low temperatures (10 °C and 15 °C) than the temperate strains (Table S4). When the 35 dpi plants were analyzed, R1, R13, R26, and R32 proved to be the most effective in clover root nodulation at 10 °C, whereas strains R1, R53, R70, and R137 exhibited the highest efficiency at 25 °C.

Next, a comparative analysis of the nodulation efficiency of the strains from both populations was performed. Based on this, only moderate differences between two climate populations were found (Table 2). The highest difference was found for the 14 dpi plants at 10 °C, which were nodulated 2 times more effectively by the subpolar strains than the temperate strains. A slight difference in this parameter was also observed at 15 °C for the 14 dpi and 21 dpi plants. This tendency was inverted at 20 °C and 25 °C. The temperate strains induced little more root nodules than the subpolar strains at all the time points (7–35 dpi). However, the differences observed were not statistically significant, (*p* ≥ 0.05). Thus, our results confirm similar adaptation of both the temperate strains and subpolar strains in a wide range of temperatures.

**Table 2:** Efficiency of clover root nodulation by *R. leguminosarum* sv*. trifolii* strains examined in a wide range of temperatures.

Origin of population	Strains	T. (°C)	Average number of nodules per plant (dpi)				
			7	14	21	28	35
Temperate climate region	2-2, 3-1, 3-3, 4-3, 5-8, 6-11, 8-3, 8-11, 10-3, KW1-9, KW2-9, M2, M14, M16, M19, 24.2	10	0 ± 0^(aA)^	0.07 ± 0.05^(aB)^	0.93 ± 0.63^(aC)^	2.06 ± 1.0^(aCD)^	2.82 ± 1.1^(aD)^
		15	0 ± 0^(aA)^	0.44 ± 0.21^(bB)^	1.46 ± 1.16^(aBC)^	2.54 ± 1.5^(aC)^	3.36 ± 1.1^(aC)^
		20	0.91 ± 0.54^(bA)^	4.02 ± 1.4^(cB)^	7.16 ± 3.5^(bBC)^	8.95 ± 3.45^(bC)^	10.27 ± 2.9^(bC)^
		25	2.46 ± 0.69^(cA)^	5.94 ± 2.1^(cB)^	7.68 ± 2.82^(bBC)^	10.41 ± 3.9^(bC)^	12.22 ± 2.0^(bC)^
Subpolar climate region	R1, R13, R23, R26, R32, R41, R49, R51, R53, R56, R66, R70, R108, R118, R137	10	0 ± 0 ^(aA)^	0.14 ± 0.1^(aB)^	1.31 ± 0.51^(aC)^	2.35 ± 0.95^(aC)^	3.16 ± 1.54^(aCD)^
		15	0 ± 0^(aA)^	0.55 ± 0.25^(bB)^	1.75 ± 0.9^(aC)^	2.80 ± 1.2^(aC)^	3.77 ± 0.78^(aCD)^
		20	0.88 ± 0.22^(bA)^	3.29 ± 1.1^(cB)^	7.56 ± 2.2^(bC)^	10.14 ± 2.85^(bC)^	11.66 ± 3.11^(bC)^
		25	2.39 ± 0.91^(cA)^	5.85 ± 1.25^(dB)^	8.83 ± 2.3^(bB)^	12.81 ± 3.21^(bBC)^	16.97 ± 3.83^(bC)^

Results are presented as mean ± SD. Lower case letters in brackets indicate statistically significant differences (*p* ≤ 0.05) for each population tested at different temperatures at a particular time point (dpi); upper case letters in brackets indicate statistically significant differences (*p* ≤ 0.05) for each population tested at particular temperatures at different time points (dpi) (two-way ANOVA, Tukey’s post hoc test). No statistically significant differences (*p* ≤ 0.05) between two populations tested at each temperature and time point (dpi) were found.

In order to check whether differences in root infection and nodule formation effectiveness observed between the studied strains could be caused by differences in their growth rate in the rhizosphere and on the root, we have performed an experiment, in which growth kinetics of the strains was examined at 10, 15, 20 and 25 °C during 0–96 h (Tables S5 and S6). In general, the tested strains grew very fast and effectively at 25 °C. However, they grew slower at 20 °C and 15 °C and very slow and poorly at 10 °C. Only moderate differences between the growth rate of the strains were observed when the data for the particular temperature were analyzed. Among the strains tested, 3-1, 3-3, 4-3, 6-11, 8-11, M14, M19, 24.2, R26, R32, R108, R118, R137 were characterized by fast and robust growth at higher temperatures (20–25 °C), whereas 2-2, 6-11, M14, M16, R13, R26, R49, R108, R118, R137 were characterized by faster and more intensive growth at low temperatures (10–15 °C). The strains 6-11, M14, R26, R108, R118, and R137 were distinguished from others by higher growth rates in a wide range of the tested temperatures. However, faster and more robust growth of these strains did not translate on their root infection and nodule formation effectiveness (Tables S1–S4). Since identical bacterial dose was added to each clover root in the case of all strains, moderate differences in growth rates observed between the individual strains seems not to have a dominant role in their effectiveness of host root infection and nodule formation under tested conditions.

### Determination of weight of clover plants inoculated with *R. leguminosarum* sv. *trifolii* strains

**Figure 2:**
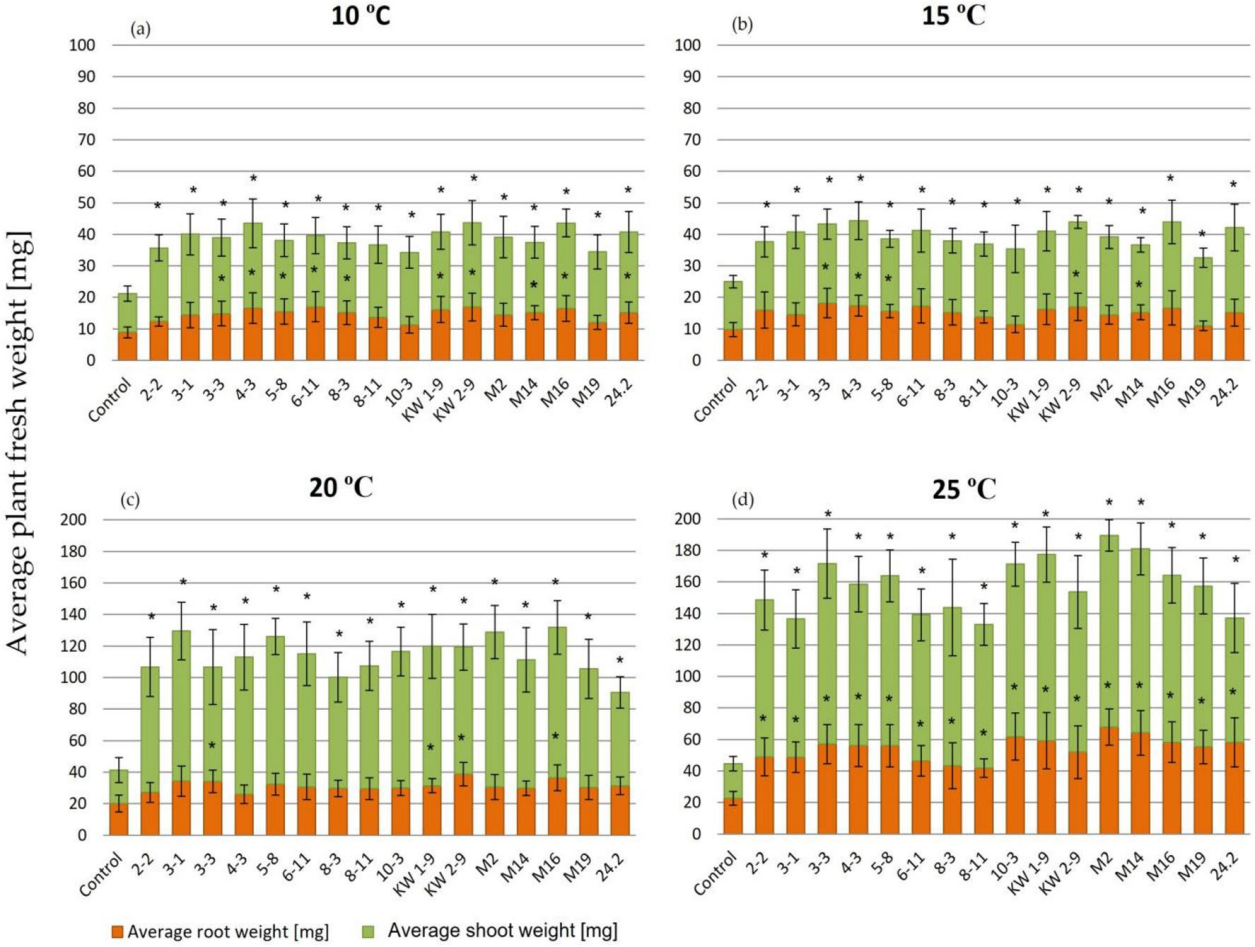
Weight of 35 dpi clover plants inoculated with the *R. leguminosarum* sv. *trifolii* strains derived from the temperate climate region and cultivated in a wide range of temperatures (10–25 °C). Results are presented as mean ± SD, statistically significant differences compared to the control (uninfected plants) at *p* ≤ 0.05 (*) (ANOVA, post hoc Tukey’s test).

**Figure 3:**
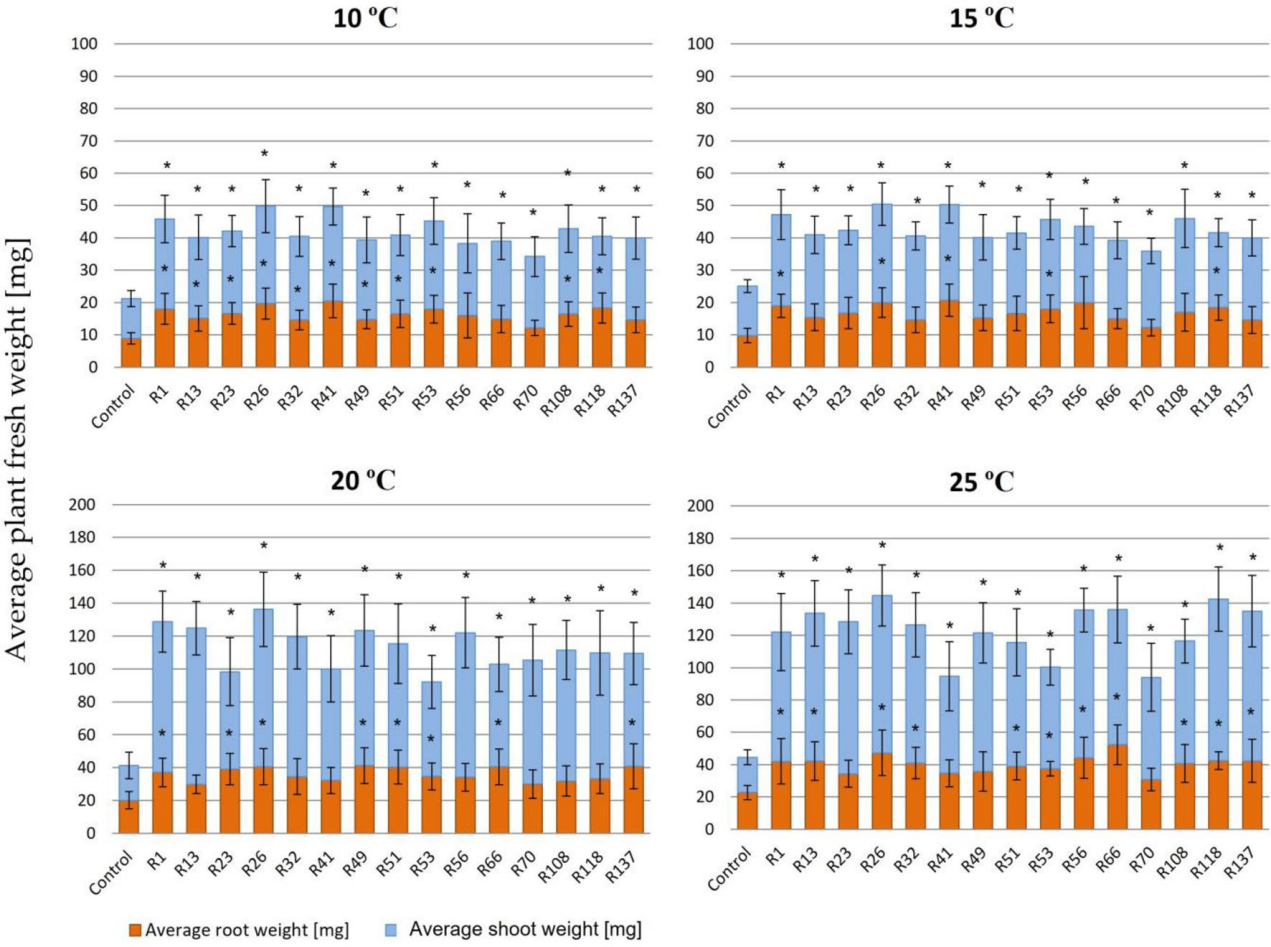
Weight of 35 dpi red clover plants inoculated with the *R. leguminosarum* sv. *trifolii* strains derived from the subpolar climate region and cultivated in a wide range of temperatures (10–25 °C). Results are presented as mean ± SD, statistically significant differences compared to the control (uninfected plants) at *p* ≤ 0.05 (*) (ANOVA, post hoc Tukey’s test).

Subsequently, the weight and length of 35 dpi clover plants grown in the N-free medium in a wide range of the temperatures (10–25 °C) were determined (Figs. 1, 2, and 3, Table 3). These parameters reflect, among others, the efficiency of the symbiotic process occurring inside legume root nodules. The appearance of 35 day clover plants (and their roots with nodules) inoculated by two representatives from each studied population and grown at low (10 °C) and optimal (20 °C) temperatures, respectively, is shown in Fig. 1.

**Table 3:** Average weight of clover plants inoculated with the temperate and subpolar *R. leguminosarum* sv. *trifolii* strains and grown in a wide range of temperatures (10–25 °C).

Plants inoculated with	Average fresh weight of clover plants (mg)				Average fresh shoot weight (mg)				Average fresh root weight (mg)			
	10 °C	15 °C	20 °C	25 °C	10 °C	15 °C	20 °C	25 °C	10 °C	15 °C	20 °C	25 °C
Temperate strains	38.3 ± 5.4^(aA)^	39.6 ± 4.1^(aA)^	114.1 ± 17.6^(bA)^	157 ± 32.1^(bA)^	24.3 ± 3.1^(aA)^	24.5 ± 3.3^(aA)^	82.6 ± 11^(bA)^	103 ± 13^(bA)^	14.6 ± 2.2^(aA)^	15.3 ± 3.1^(aA)^	29.4 ± 6.7^(bA)^	53.1 ± 9.4^(cA)^
Subpolar strains	41.9 ± 8.2^(aA)^	43 ± 6.6^(aA)^	115.8 ± 22.2^(bA)^	121.8 ± 23^(bA)^	25.5 ± 3.5^(aA)^	26.1 ± 4.3^(aA)^	78.9 ± 12^(bA)^	81 ± 18^(bA)^	16.4 ± 4.0^(aA)^	16.9 ± 3.1^(aA)^	36.1 ± 4.3^(bA)^	40.3 ± 9.6^(bA)^
Control plants	21.2 ± 2.4^(aB)^	25 ± 3.1^(aB)^	41.2 ± 3.5^(bB)^	44.5 ± 3.6^(bB)^	12.3 ± 2.1^(aB)^	15.2 ± 3.3^(aB)^	21.1 ± 3.1^(bB)^	21.8 ± 4^(bB)^	8.9 ± 1.2^(aB)^	9.8 ± 1.5^(aB)^	20.1 ± 2^(bB)^	22.7 ± 3.2^(bB)^

Results are presented as mean ± SD. Lower case letters in brackets indicate statistically significant differences (*p* ≤ 0.05) for plants inoculated with the strains from each population tested and control plants tested at different temperatures; upper case letters in brackets indicate statistically significant differences (*p* ≤ 0.05) for plants inoculated with the strains from both populations and uninoculated plants tested at particular temperatures (two-way ANOVA, post hoc Tukey’s test). No statistically significant differences (*p* ≤ 0.05) between two populations tested at each temperature and time point (dpi) were found.

Plants with no bacterial inoculation were used as a control. In general, we observed that, together with the increase in the temperature (from 10 to 25 °C), the length and weight of the entire plants as well as their shoots and roots importantly increased. Moreover, these two parameters of clover plants inoculated with the rhizobial strains were essentially higher than those of the uninoculated plants. This was found in all the temperature variants, indicating that the tested strains are effective clover microsymbionts with high biomass production potential. The plants grown at 20 °C and 25 °C were about twice longer than those grown at 10 °C and 15°. Similar results were obtained for the plants infected by the strains from both climatic populations. The control plants grown at all the tested temperatures were much smaller than those inoculated with rhizobia (data not shown).

The determination of the fresh weight of clover plants inoculated with the rhizobial strains and grown in a wide range of temperatures (10–25 °C) revealed higher differences between the individual strains at the particular temperature and between the temperatures for the individual strains tested than those found for the plant length data.

In general, the results obtained indicated that low temperatures (10–20 °C) and N absence were factors limiting clover growth (Figs. 2 and 3). The plants inoculated with rhizobia had essentially higher weight than the uninfected plants in all the temperature variants (from 1.97 times at 10 °C to 3.53 times at 25 °C) (Table 3). The differences in the shoot weight between the inoculated and uninoculated plants were even higher (from 1.97 times at 10 °C to 4.74 times at 25 °C).

Moreover, the weight of the plants inoculated with temperate and subpolar strains was compared. In general, the plants infected by these bacteria and grown at 25 °C showed very high total and shoot weights, respectively (Table 3, Figs. 2 and 3). In these conditions, more temperate strains were highly efficient in symbiosis than the subpolar strains. The differences observed in biomass production between these plants probably reflected a symbiotic N-fixing capacity of the particular strains examined. The plants cultivated at lower temperatures (10–20 °C) had essentially lower weights than those cultivated at 25 °C. Slightly higher total mass (and the mass of shoots and roots) was observed when they were inoculated with the subpolar-origin bacteria than those inoculated with the temperate strains. These data indicate that more subpolar strains were symbiotically efficient in plant mass production at low temperatures (10–15 °C) than the temperate strains (Table 3). When biomass production efficiency of the individual strains was analyzed in detail, 3-1, 4-3, 6-11, KW1-9, KW2-9, M2, M16, and 24.2 proved to be the most efficient at the low temperature (10 °C) among the temperate strains (Fig. 2a) (the average weight of the total plants and their shoots was ~ 40 mg and over 25 mg, respectively). The majority of the tested subpolar strains were highly effective in N fixation at 10 °C (Fig. 3a), since plants weighing ~ 40 mg were found for all strains with the exception of R49, R56, R66, and R70. At high temperature (25 °C), the most efficient strains were 2-2, 3-3, 4-3, 5-8, 10-3, KW1-9, KW2-9, M2, M14, M16, and M19 (Fig. 2d) (the average plant and shoot weight exceeded 150 mg and 100 mg, respectively). A majority of the tested subpolar strains were not as effective as the temperate strains at 25 °C (Fig. 3d) (plants inoculated with these bacteria did not achieve the weight of 150 mg). Only plants inoculated with R26 and R118 yielded the total mass of 140 mg and the shoot weight above 100 mg.

### Determination of nitrogenase activity and N concentration in clover plants inoculated with *R. leguminosarum* sv.* trifolii* strains

**Figure 4:**
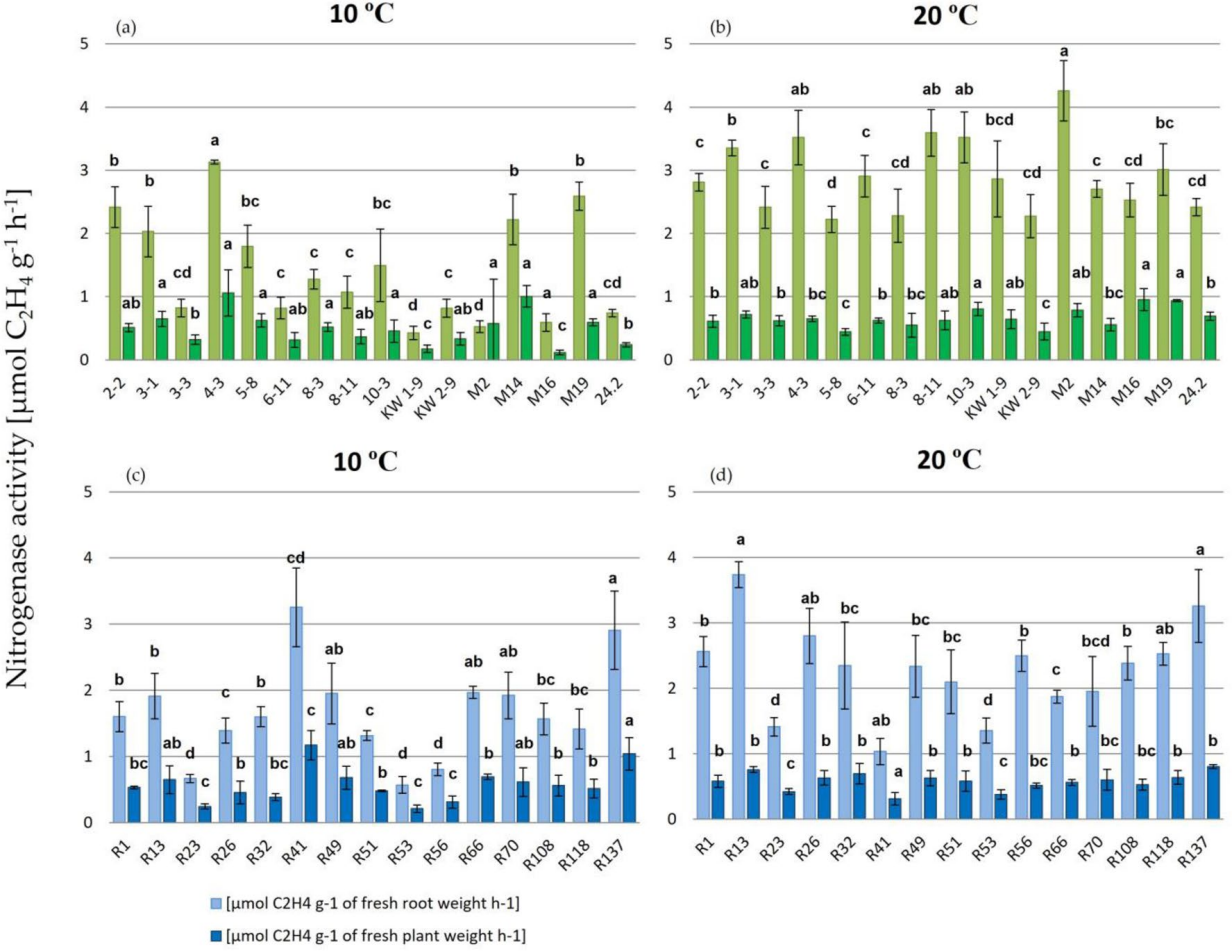
Nitrogenase activity estimated in root nodules of 35 dpi clover plants inoculated with *R. leguminosarum* sv. *trifolii* strains from two geographic regions and cultivated at 10 °C and 20 °C (temperate strains—green bars, subpolar strains—blue bars). Results are presented as mean ± SD; statistically significant differences in the nitrogenase activity determined at individual temperatures in roots of plants inoculated with different strains (*p* ≤ 0.05; ANOVA, post hoc Tukey’s test) are marked with different letters.

In a further work, other symbiotic properties of the rhizobial strains that are important in legume mass production efficiency were determined. First, nitrogenase activity in root nodules of the 35 dpi plants was determined using an acetylene reduction assay (ARA), in which ethylene production from acetylene was assessed. This rhizobial enzyme plays a crucial role in symbiotic N fixation, since it is responsible for N_2_ reduction to NH_4_^+^ ions. Although ARA has some limitations related with its sensitiveness to plant disturbance and sometimes poor correlation between ARA and %N in plant biomass, this technique was useful and suitable for confirmation and determination of the nitrogenase activity in clover root nodules occupied by individual strains. The activity of this symbiotic system was determined at two temperatures (10 °C and 20 °C). The obtained values were calculated per g of fresh plant weight and per g of fresh root weight, respectively (Fig. 4), since clover root nodules are too small to obtain appropriate mass of fresh nodules (especially at 10 °C, when the number of nodules formed is low). The roots of clover plants uninfected by rhizobia were used as a negative control. These plants had no nodules and, in a consequence, no ethylene production ability (data not shown).

In general, our data confirmed that all the studied strains exhibited the nitrogenase activity and were fixing N at both the tested temperatures (Fig. 4). Moreover, the activity of this symbiotic system observed as the ethylene production effect was higher when this parameter was determined at 20 °C than 10 °C, indicating that the temperature has an important influence on this enzymatic activity. This effect was observed in the plants inoculated with all the tested strains irrespective of their climate origin. Furthermore, some differences among the strains were detected, when this parameter was determined at the same temperature. For an example, this activity in the nodules of the plants inoculated with the temperate strains was in the range from 0.43 (KW1-9) to 2.59 (M19) at 10 °C (Fig. 4a) and from 2.22 (5–8) to 4.2 (M2) µmol g^−1^ h^−1^ of fresh roots at 20 °C, respectively (Fig. 4b). In the case of the subpolar strains, the activity was in the range of 0.66 (R23)–3.25 (R41) at 10 °C (Fig. 4c) and 1.35 (R53)–3.73 (R13) at 20 °C, respectively (Fig. 4d). The highest activity at 10 °C (values higher than 1.3) was exhibited by temperate strains 3-1, 4-3, KW2-9, M16, and M19 (Fig. 4a) and subpolar strains R1, R13, R26, R32, R41, R49, R51, R66, R108, R118, and R137 (Fig. 4c). In turn, the highest enzymatic activity at 20 °C (values higher than 3.0) was determined for 3-1, 4-3, 8-11, 10-3, KW2-9, M2, and M16 (temperate strains) (Fig. 4b) and R13, R49, and R137 (subpolar strains) (Fig. 4d). Thus, our data show a diversity in respect to the activity of this symbiotic system among rhizobial strains. The average values for all the temperate strains determined at 10 °C and 20 °C were 1.13 ± 0.5 and 2.97 ± 0.65 µmol g^−1^ h^−1^, respectively, whereas the average values for all the subpolar strains determined at 10 °C and 20 °C were 1.5 ± 0.75 and 2.52 ± 1.1 µmol g^−1^ h^−1^, respectively. However, the differences between the two populations observed at the same temperature were not statistically significant.

**Figure 5:**
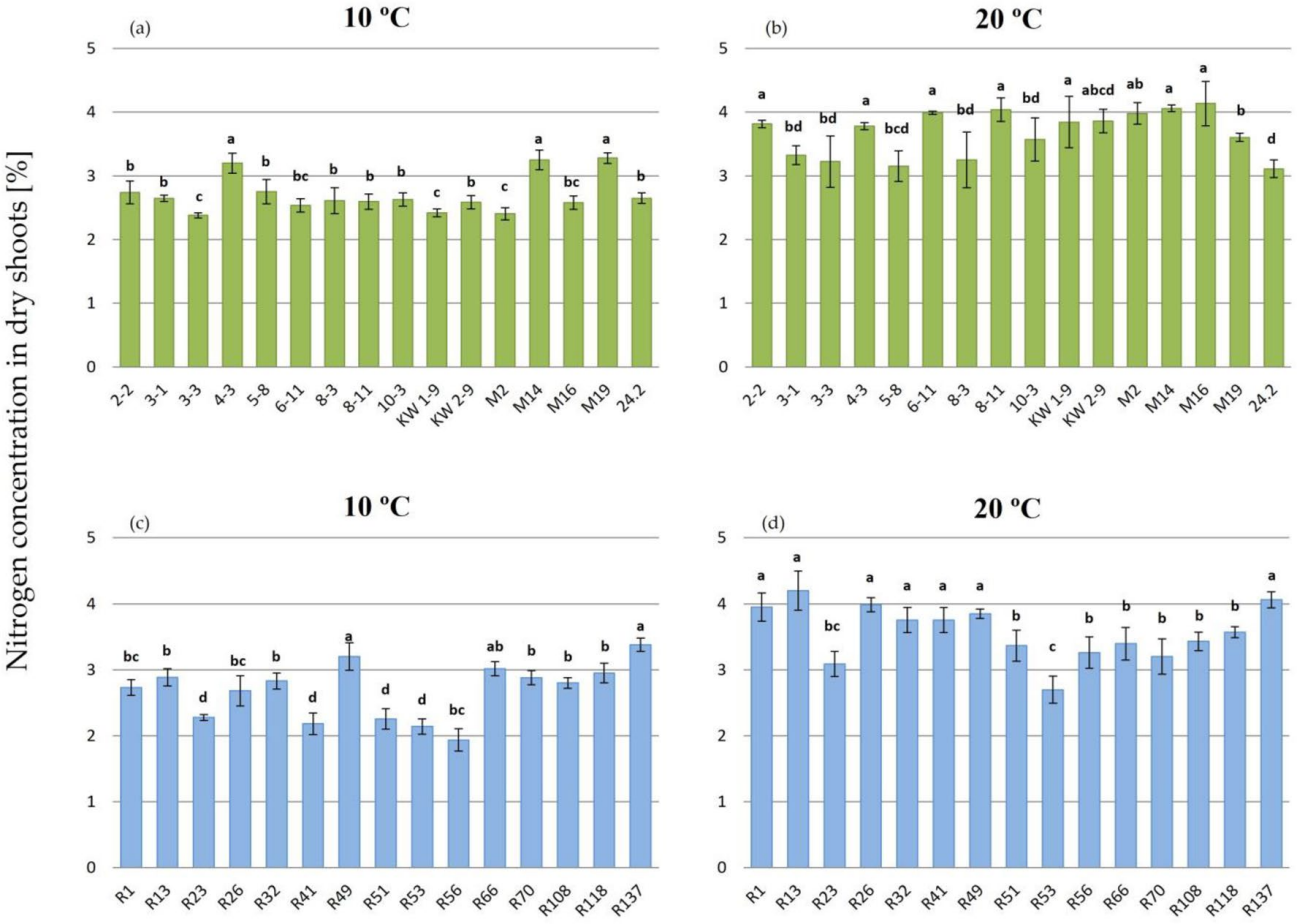
Nitrogen concentration in dry shoots of 35 dpi clover plants inoculated with *R. leguminosarum* sv. *trifolii* strains from two geographic regions and cultivated at 10 °C and 20 °C (temperate strains—green bars, subpolar strains—blue bars). Results are presented as mean ± SD; statistically significant differences in %N determined in dry shoots of plants inoculated with different strains and grown in individual temperatures (*p* ≤ 0.05; ANOVA, post hoc Tukey’s test) are marked with different letters.

Next, N concentration (%) in the dry shoots of the 35 dpi clover plants cultivated at 10 °C and 20 °C was also determined. In general, the plants grown at 20 °C were characterized by higher %N than those grown at 10 °C, regardless of the strain used for plant inoculation (Fig. 5). The average values calculated for the plants inoculated with all the temperate and subpolar strains were very similar in both temperature variants: 2.71%N at 10 °C and 3.71%N at 20 °C (*p* = 0.211) in the case of the temperate population and 2.7%N at 10 °C and 3.53%N at 20 °C in the case of the subpolar population (*p* = 0.267). The analysis of the plants inoculated with the individual strains revealed some differences in this parameter. The %N in dry shoots of the plants inoculated with the temperate strains was in the range from 2.38 (3–3) to 3.28 (M19) at 10 °C (Fig. 5a) and from 3.15 (5–8) to 4.13 (M16) for 20 °C (Fig. 5b), respectively. The higher diversity with respect to this symbiotic parameter was found for the subpolar strains (at both temperatures), since %N ranged from 1.93 (R56) to 3.38 (R137) at 10 °C (Fig. 5c) and from 2.43 (R23) to 4.2 (R13) at 20 °C (Fig. 5d). Thus, our data indicate that both the temperature at which the clover plants were cultivated and the strain type used for host inoculation had an influence on %N in clover shoots.

### Determination of the genetic diversity and phylogenetic relatedness of red clover microsymbionts using two symbiotic *nodC* and *nifH* genes

**Figure 6:**
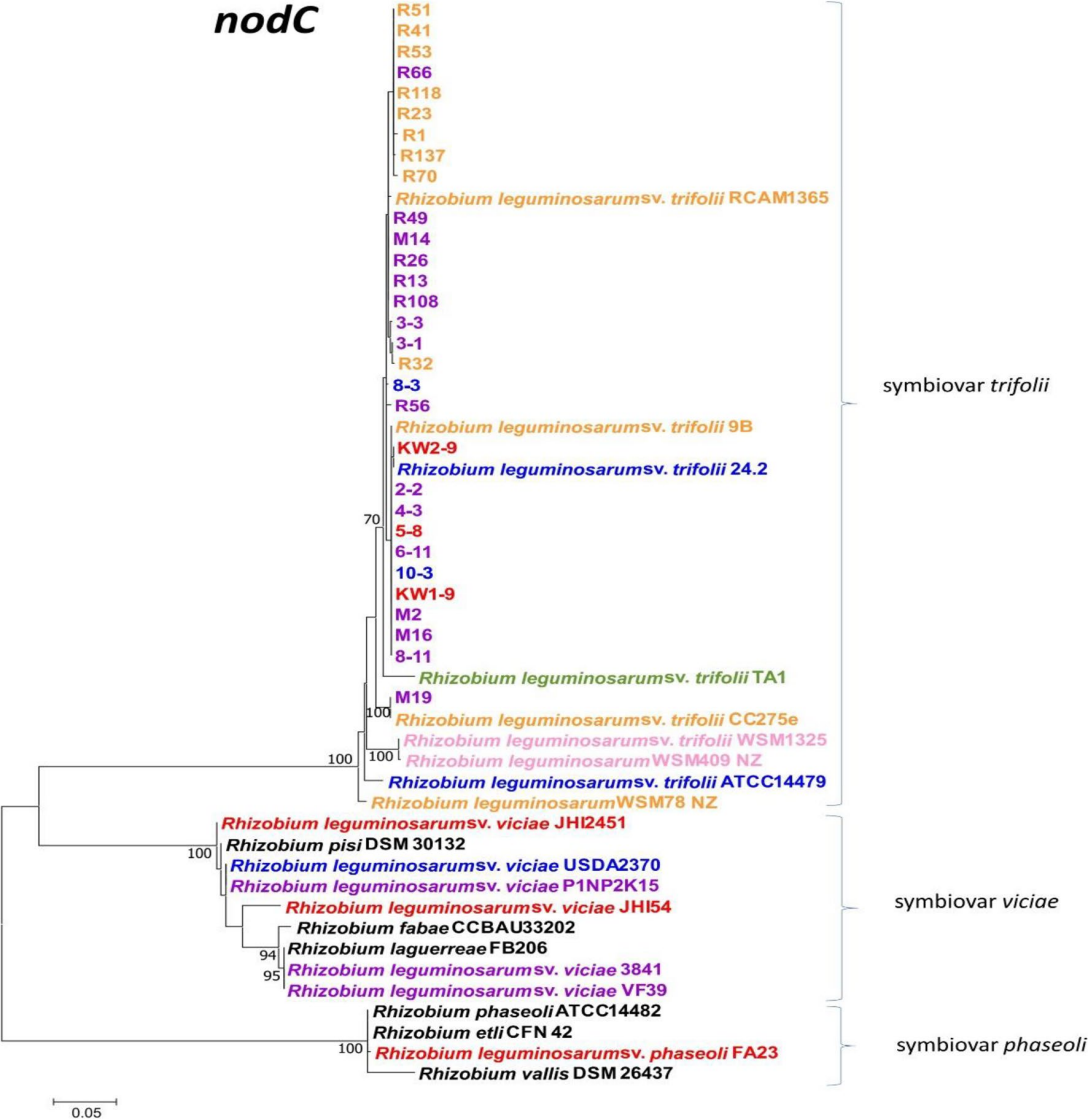
Maximum Likelihood (ML) tree based on 548-bp long *nodC* sequences showing relationships of the red clover isolates with selected members of different *R. leguminosarum* sv. belonging to various gs and reference strains of different *Rhizobium* species. The colors indicate gs: orange—gsA, purple—gsB, green—C, pink—H, blue—E, red—K. Bootstrap values (based on 1000 replicates) are shown on the branches. The scale bar represents the number of nucleotide substitutions per site. The phylogenetic analysis was conducted in MEGAX using the Maximum Likelihood algorithm with the General Time Reversible model plus Invariant site plus Gamma rate distribution (GTR + I + G).

**Figure 7:**
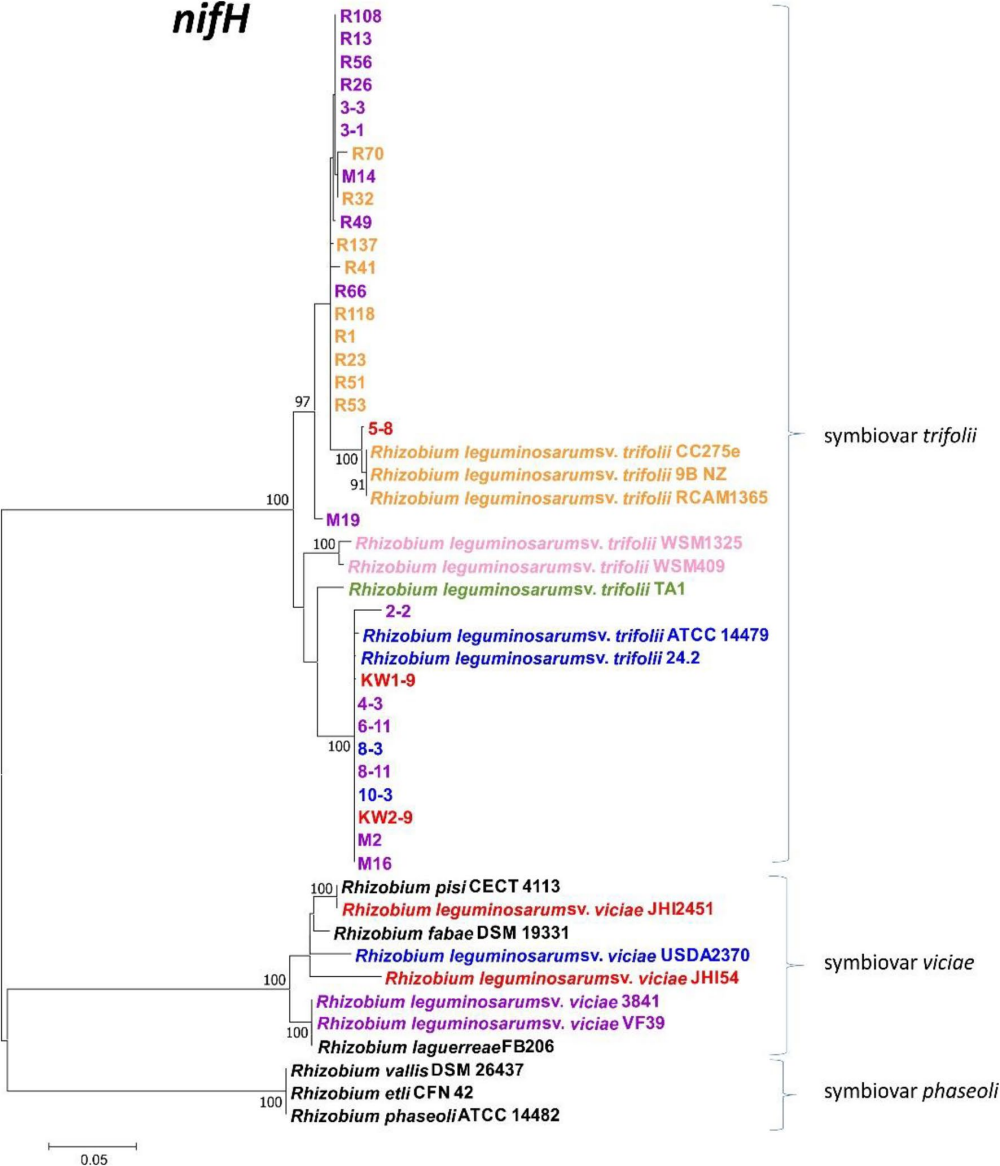
Maximum Likelihood tree based on 747-bp long *nifH* sequences showing relationships of the red clover isolates with selected members of different *R. leguminosarum* sv. belonging to various gs and reference strains of different *Rhizobium* species. The colors indicate g: orange—gsA, purple—gsB, green—C, pink—H, blue—E, red—K. Bootstrap values (based on 1000 replicates) are shown on the branches. The scale bar represents the number of nucleotide substitutions per site. The phylogenetic analysis was conducted in MEGAX using the Maximum Likelihood algorithm with the General Time Reversible model plus Invariant site plus Gamma rate distribution (GTR + I + G).

Previously, the red clover isolates were genetically characterized using 16S rRNA and multilocus sequence analysis (MLSA) of five house-keeping genes (*atpD, recA, rpoB, gyrB,* and *glnII*)^46^. Based on these analyses, the examined strains were classified to *R. leguminosarum* species. In this study, we characterized the symbiotic diversity of these *T. pratense* root nodule isolates. For this purpose, two key symbiotic markers, *nodC* and *nifH,* were sequenced and their phylogenetic analysis was performed (Figs. 6, 7). The *nodC* gene encodes an enzyme involved in the synthesis of chitin oligosaccharide backbones of the Nod factor, whereas the *nifH* gene encodes the Fe protein of nitrogenase^7,48,49^.

The comparative analysis of 548-bp long *nodC* sequences revealed 13 alleles among the 31 studied strains, of which 6 alleles were unique to 11 subpolar isolates, 6 were unique to 14 temperate isolates, and one allele was shared by 1 temperate isolate and 4 subpolar isolates. In total, 21 polymorphic sites were identified in the *nodC* genes of the studied strains. The temperate strains showed 97.8–100% *nodC* sequence similarity, whereas the *nodC* sequences of the subpolar strains were similar in 98.9–100%. The sequences of this gene in all the 31 studied strains were similar to each other in the range of 97.2–100%. One temperate strain M19 was the least similar to the other 30 strains and, after excluding it from the analysis, the sequence identity of the *nodC* gene between the temperate and subpolar strains increased to 98.7–100%. Thus, our results indicated a low level of *nodC* sequence heterogeneity between the analyzed strains despite their different geographic origin. The root nodule isolates from both geographic regions had *nodC* sequences most similar to the reference *R. leguminosarum* sv. *trifolii* strains (95.2–100%), supporting affiliation of the studied strains to this symbiotic variant.

All the studied and reference *R. leguminosarum* sv. *trifolii* strains were compared to representatives of sv. *viciae* and *phaseoli*. The sv. *trifolii* strains shared 73.5–75.9% and 67.5–69.5% *nodC* sequence similarity with strains representing sv. *viciae* and *phaseoli*, respectively. The *nodC* sequence similarity ranged from 93.9% to 100% in the group of the reference strains belonging to the sv. *viciae* and from 96.3 to 100% among the reference strains belonging to the sv. *phaseoli*. Moreover, the reference strains of the sv. *viciae* were similar to those of the sv. *phaseoli* in the range of 72.2–74.2%, indicating that there is a clear gap in the *nodC* sequence similarities between different *R. leguminosarum* sv.

In the maximum likelihood (ML) *nodC* phylogram, the studied strains and *R. leguminosarum* sv. *trifolii* representatives formed a common cluster that was clearly separated from the two other sv., i.e. *viciae* and *phaseoli*, which also formed groups separated from each other (Fig. 6). All the three sv. clusters were supported by 100% bootstrap values. 30 out of the 31 studied strains were grouped with *R. leguminosarum* sv. *trifolii* strains 9B and RCAM1365, which were also isolated from *T. pratense* root nodules, although this cluster was not well supported by bootstrapping. Two subclusters and two separate branches were identified within this cluster; one included 10 of the 15 temperate isolates and reference strains 9B and 24.2, whereas the other subgroup encompassed 14 subpolar and 3 temperate isolates as well as reference strain RCAM1365. Strains 8–3 and R56 were found on separate branches within this large cluster. The remaining M19 isolate differed from the other 30 studied strains at 9 nucleotide positions of the analyzed *nodC* gene fragment but showed 100% sequence identity of *nodC* with the gene from strain *R. leguminosarum* CC275e, with which it formed a separate branch in the *nodC* phylogenetic tree.

Similarly to *nodC,* the sequence analysis of 747-bp long fragments of the *nifH* gene in *T. pratense* microsymbionts allowed identification of 13 alleles. However, a higher level of *nifH* heterogeneity was estimated for the studied strains in comparison to the *nodC* sequence, which were similar to each other in the range of 94.2–100%. Moreover, a threefold higher number (i.e., 61) of polymorphic sites were identified in the sequences of *nifH* than in the *nodC* sequences. As in the case of the *nodC* gene, the isolates from both climatic regions were characterized by the highest *nifH* sequence similarity with the *R. leguminosarum* sv. *trifolii* representatives (94.2–100%), confirming their close symbiotic relationships with the reference strains isolated from *Trifolium* spp. root nodules. All the strains exhibited *nifH* sequences that were considerably less similar to those of the *nifH* gene of the reference strains belonging to the sv. *viciae* (79.1–82.1%) and *phaseoli* (81.5–82.7%). The representative strains of both these sv. showed 81.9–82.5% similarity of *nifH* sequences to each other.

Based on the phylogenetic analysis of the *nifH* gene, the studied isolates and the reference rhizobial strains were grouped into three well-defined clusters that corresponded to sv. *trifolii, viciae*, and *phaseoli* (Fig. 7). All the clusters were supported by 100% bootstrap values. All the analyzed *T. pratense* root nodule isolates clustered with the reference strains of the sv. *trifolii*. The overall grouping of the studied strains within the sv. *trifolii* clade was similar to that identified in the *nodC* phylogram, with some differences. Interestingly, all the examined subpolar strains were grouped with 4 temperate strains (3-1, 3-3, M14, and 5-8) and 3 reference strains CC275e, 9B, and RCAM1365. The second subclade included 10 temperate isolates and 5 reference *R. leguminosarum* strains (ATCC 14,479, TA1, WSM1325, WSM409, 24.2). As in the *nodC* analysis, M19 was found on a separate branch (Fig. 7).

Next, the analysis of the *nodC* and *nifH* alleles was performed in more detail. In general, 13 alleles for *nodC* and 13 alleles for *nifH* were identified within the 31 strains of *R. leguminosarum* sv. *trifolii* representing both climatic zone populations. Among the *nodC* alleles, the most frequent were those marked as type I (identified in 9 strains: 4-3, 6-11, 8-11, M2, M16, 10-3, 2-2, 5-8, KW1-9), type II (in 6 strains: R23, R41, R51, R53, R66, and R118), and type III (in 5 strains: R13, R26, R49, R108, M14) (Table 4, strains marked in blue color). The other alleles, designated as IV–XIII, proved to be unique for particular strains (Table 4, marked in yellow). A similar tendency was found in the case of *nifH,* namely, three types were the most frequent among the identified alleles: type I in 8 strains (4-3, 6-11, 8-11, 8-3, M2, M16, 10-3, KW2-9), type II in 6 strains (R1, R23, R51, R53, R66, and R118), and type III in 6 strains (R13, R26, R56, R108, 3-1, 3-3). Type IV was found in two strains (R32 and M14), whereas the other *nifH* alleles (V–XIII) were unique for individual strains (Table 4). Based on these results, we determined the *nodC-nifH* genotypes for the studied strains. In total, 20 *nodC-nifH* genotypes were identified. Among them, three genotypes, marked as A (6 strains), B (5 strains), and C (3 strains), were the most frequent. Type A was found exclusively in the temperate strains, whereas types B and C were determined for the subpolar strains. The other 17 *nodC-nifH* genotypes were found uniquely in single strains. The *nodC-nifH* genotypes determined for the reference strains were also unique (Table 4, marked in light grey). Thus, our data indicate that nearly half of the analyzed strains (14) represented frequent symbiotic genotypes based on the *nodC* and *nifH* alleles, whereas the other strains possessed unique *nodC-nifH* genotypes.

**Table 4:**
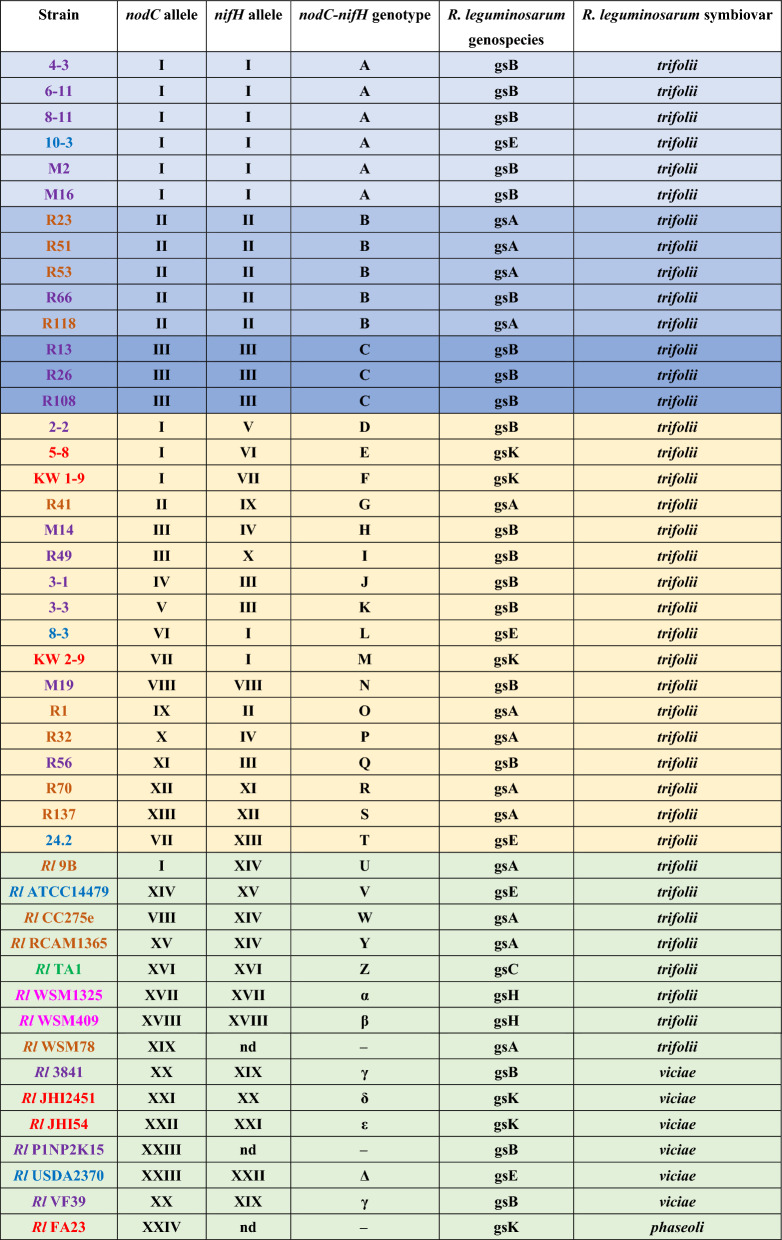
List of *nodC* and *nifH* alleles and *nodC-nifH* genotypes identified in the studied *R. leguminosarum* sv. *trifolii* and the reference strains used in the phylogenetic analyses.

*Rl* Rhizobium leguminosarum, *nd* no data. The font colors indicate Rl genospecies orange—gsA, purple—gsB, green—gsC, pink—gsH, blue—gsE, red—gsK; background colors indicate types of *nodC-nifH* genotype: light blue—A, medium blue –B, dark blue—C, yellow—genotypes unique for individual strains, light green—genotypes of reference Rl strains.

Furthermore, we compared the symbiotic genotypes of the studied strains with their membership in the *R. leguminosarum* genospecies (gs) determined by us earlier based on MLSA of the house-keeping genes (*atpD, recA, rpoB, gyrB,* and *glnII*)^46^. The occurrence of multiple genospecies (18) within the *R. leguminosarum* species complex has been confirmed recently by Young and colleagues^14^. In accordance with this finding, we assigned these 31 *R. leguminosarum* sv. *trifolii* strains into four gs (A—9 strains, B—16 strains, K—3 strains, and E—3 strains). Among them, gsA and gsB were the most frequently represented. The temperate strains belonged to B, E, and K, whereas the subpolar strains were representatives of A and B (Table 4).

## Discussion

Legumes, comprising 22,360 species, are among the largest group of angiosperms representing the outcome of a high diversification rate^18,50^. The successful adaptation of these plants to new climates and/or ecological niches has contributed to their occupancy in diverse habitats. Among these plants, there are many commercial legumes that not only are important sources of food and forage crops^6,21^, but also are cultivated as green manure and pioneering plants to restore damaged lands^51–53^. Legumes are able to establish a symbiosis with rhizobia residing inside special nodular structures on host roots. The efficiency of the rhizobium-legume symbiosis is modulated by the specificity and effectiveness of natural populations of rhizobia^11,54,55^. In exchange for carbon nutrients from the host, rhizobia convert atmospheric N_2_ into its usable form, thus making the plant self-sufficient in N requirements^18,21,56^. N is one of the crucial macronutrients required for plant growth and productivity. Given the benefits offered by rhizobia, the practice of inoculating various legumes with competitive strains to increase BNF effects is promising^7,11,12,15^. Hence, there is a need to identify and select rhizobial strains from the natural environment that will be highly efficient in various stress conditions.

Therefore, to broaden our knowledge in this field, we characterized the symbiotic performance of *T. pratense* microsymbionts isolated from root nodules of plants grown in the subpolar and temperate climate zones. To date, no comparative analysis of the symbiotic efficiency and heterogeneity of *R. leguminosarum* sv. *trifolii* strains originating from two distinct geographic regions differing essentially in annual and daily temperature profiles has been performed. We estimated various symbiotic parameters (host root infection and nodule formation effectiveness, nitrogenase activity) of *T. pratense* isolates and estimated their biomass productivity in different temperature conditions (10–25 °C). In addition, the symbiotic heterogeneity of the strains was determined based on the sequence analysis of two symbiotic *nodC* and *nifH* genes and compared with their genomic diversity estimated on the basis of five house-keeping genes (*atpD, recA, rpoB, gyrB, glnII*).

In this study, we confirmed that all the tested strains representing both climate populations were highly efficient in BNF, although some differences between the two populations and between the strains within the individual populations were observed. In general, all these strains effectively infected *T. pratense* roots and induced nodules in a wide range of temperatures, indicating that the particular strains were very well-adapted to these environmental conditions (Tables 1, 2 and S1–S4). However, the low temperature had a negative effect on the infection and nodulation processes, since the time needed for the formation of the first nodules was longer and the number of nodules induced at 10 °C was ~ four–fivefold lower in comparison to the number of nodules on plants cultivated at 25 °C. Moderate differences in the growth rate observed between the strains (Tables S5–S6), suggested that this physiological trait has not the dominant influence on root infection and nodulation effectiveness under tested conditions.

With respect to biomass production, all the tested strains were also highly efficient. The plants inoculated with these bacteria had significantly higher weight than the weight of the uninoculated (control) plants (from ~ 2 to 3.7 times, depending on the strain and temperature used) (Figs. 1, 2, and 3). Generally, the clover growth and biomass production supported by BNF were temperature-dependent, and 20–25 °C proved to be optimal for these processes. The nitrogenase activity was also essentially higher at 20 °C than at 10 °C (~ twofold) (Fig. 4). Our data are in congruence with the findings reported earlier by Ryle and others^57^, who confirmed the effect of temperature on nitrogenase activity. These authors estimated that the nitrogenase activity in white clover root nodules increased together with an increase in temperature from 5 to 25 °C, and a linear relationship was found. Interestingly, similar activity levels at days and nights were found at the same temperature, indicating that the action of this enzyme is light independent^57^. Moreover, our data show a moderate diversity in the activity of this symbiotic system among the strains tested at the same temperature.

The N concentration in the dry shoot matter both for individual strains and each of the particular populations tested were also determined. The high efficiency of this process was confirmed (Fig. 5). However, some differences between the plants inoculated with the individual strains were found. The plant cultivation temperature also had an influence on this symbiotic parameter, since %N was higher in the plants grown at 20 °C than in those grown at 10 °C. As shown by other data, the average %N in clover hay is 2.45%, and this value depends on several factors, e.g. the plant type and its growth phase, plant parts, soil fertility, type and intensity of fertilization, and weather conditions^58^. Our data indicate that the growth conditions and the strains used in this experiment resulted in high clover yielding. Similar positive effects of rhizobia on %N in plant mass were also obtained in studies of other legumes^59–61^. In many papers^13,24–28,42,62^, the influence of rhizobia on plant production was studied using only one symbiotic parameter (mainly plant shoot and root weight). In this study, we characterized a symbiotic potential of the strains using three parameters; i.e., plant shoot and root weight, nitrogenase activity, and %N in dry shoot mass (Figs. 2, 3, 4, and 5). Thanks of them, more broaden view of the symbiotic performance of the strains can be obtained. Although ARA has some limitations, this technique proved to be useful for determination of the nitrogenase activity in the strains residing in clover root nodules. Based on ARA data, some differences among the strains tested under the same conditions were found, indicating that this is a strain-specific trait. However, we did not found a correlation between ARA and %N data for some of the studied strains (e.g., R41 at 10 °C), indicating that ARA is not a reliable method to quantify N fixation. This can be explained by the fact that apart from the activity of nitrogenase, action of other important components and enzymes of both symbiotic partners are also required for optimal BNF efficiency^6,9,12,16,17,21^. Based on our results, fresh plant shoot weight and %N in dry shoots are parameters which more properly show the effect of rhizobial inoculation on the plant growth and productivity.

Based on all data, a few strains from each of the studied populations (4-3, KW1-9, KW2-9, M2, M16 from the temperate population and R1, R13, R23, R26, and R137 from the temperate population) characterized by high symbiotic performance in the wide range of temperatures (Figs. 2, 3). Therefore, these strains are promising for further studies and potential applications as single inoculants or mixed formulations. This finding is in congruence with the results of a recent study conducted by Rodríguez-Navarro and others^62^, who showed that the efficiency of Spanish *Trifolium*-nodulating rhizobia was related to the clover species used, and the majority of the tested strains had high BNF potential with a wide range of clover species, making them valuable strains for inoculant manufactures. The practice of inoculating various legumes (including important food and forage crops) to increase BNF effects with highly competitive rhizobia is widespread^11–13,63^. Rhizobial strains introduced into the environment as inoculants are known to determine and enhance the symbiotic performance and N_2_ fixation rates^64^.

In the current study, the symbiotic diversity of the strains was also investigated using two essential symbiotic genes involved in nodulation (*nodC*) and nitrogen fixation (*nifH*) processes, respectively (Figs. 6, 7). Although multiple *nod* and *nif* genes are needed for establishment of successful symbiosis with compatible host plants, many studies indicate that one representative of *nod* genes (mainly *nodC* and less frequent *nodA*) and one representative of *nif* genes (*nifH* is the most often used), are very often sufficient to classify the isolates to individual symbiovar^14,53,62,65–75^. Interestingly, we detected identical numbers of alleles (13) for both *nodC* and *nifH* genes in the 31 clover root isolates representing the two climate populations. A threefold higher number of polymorphic sites in the *nifH* sequences (61) than in *nodC* (21) were detected, which indicates that the former gene has higher heterogeneity than the latter one. Among both *nodC* and *nifH* alleles, three genotypes (I-III) were the most frequent, whereas the other alleles (IV-XIII) proved to be unique for individual strains. Based on the identified types of the *nodC* and *nifH* alleles, 20 *nodC-nifH* genotypes were identified (Table 4). Among them, three genotypes marked as A (6 strains), B (5 strains), and C (3 strains) were predominant and together constituted nearly half of the examined strains (45.16%). Type A was exclusively found in the temperate strains, whereas types B and C were identified in the subpolar strains. The other 17 genotypes were unique for single strains. Thus, our results confirm the high heterogeneity of the *T. pratense* isolates within both populations. In contrast, Sbabou and others identified only 15 alleles among 202 *R. leguminosarum* strains isolated from *Vicia ervilia* plants from Northern Morocco^66^. The PCR–RFLP analysis of *nodEF* and *nifDK* intergenic regions of arctic and subarctic populations of *R. leguminosarum* sv. *trifolii* strains derived from three clover species (red clover, white clover, and alsike clover) from Northern Norway also showed a lower diversity of symbiotic genes than that in our strains. A total of 12 *nodEF* genotypes and 6 *nifDK* genotypes were identified among 75 strains^41^. Taken together, the degree of symbiotic diversity of rhizobial populations depends on several factors such as the legume host species, place of their origin, and climate conditions as well as symbiotic genes and techniques used.

It is well known that numerous rhizobial genes contribute to plant nodulation (*nod*) and nitrogen fixation (*nif, fix*) processes, and some of them are used for characterization of symbiotic relationships and host range prediction^1,14,21,53,67–75^. Among these genes, *nodC* and *nifH* are used in most studies to classify rhizobial strains to particular symbiotic variants (sv.). The term ‘symbiovar’ is not a formal taxonomic category, but this concept was proposed to describe rhizobia sharing a common assembly of genes that provide suitable host specificity^74^. Symbiovars reflect bacterial adaptation to legumes and the concept aims to group the strains within a species that are able to establish symbiosis with a specific legume^69,75^. *Trifolium* spp. plants are restrictive hosts, since they are nodulated only by strains from the symbiovar *trifolii* that mainly belong to *R. leguminosarum* and sporadically to *R. pisi* species^69^. In our earlier study, we classified these clover root isolates to *R. leguminosarum* species^46^ and results obtained in this work allowed us to classify them to the symbiovar *trifolii*. Generally, phylogenetic analysis of two symbiotic *nodC* and *nifH* genes are sufficient to assign strains to a particular symbiovar, as it was confirmed for many isolates from different species^1,14,21,53,67–75^. For an example based on the *nodC* sequences, isolates from *Phaseolus vulgaris* were classified to symbiovars *phaseoli* and *gallicum*^70^. The *nodC* and *nifH* genes were also sufficient to identify three new symbiovars *cenepequi, glycinis*, and *cajani* among isolates belonging to *Bradyrhizobium* genus derived from legumes grown in Western Australia and South Africa^73^. However, since the symbiotic information is encoded on plasmids or other mobile genetic elements that can be laterally transferred, a particular sv. can be maintained in various diverging bacteria lineages. The results of several studies have shown that horizontal transfer of symbiosis-related genes is far more frequent between closely related strains within a single species or genus than between species of different genera^7–9,37,67^. In these cases, the phylogenetic analysis of more than two symbiosis genes can be required. The transfer is usually detected as a result of phylogenetic incongruence between symbiotic and housekeeping genes and is determined by visual assessment of phylogenetic trees of the two sets of genes^4,9,14,21^. As a result of *nod* or *nif* horizontal transfer, different species can be characterized by very similar symbiotic genotypes. In this study, representatives of *R. leguminosarum*, *R. pisi*, *R. fabae*, and *R. laguerreae* share similar *nodC* and *nifH* sequences, as it is seen in the phylogeny trees (Figs. 6, 7); hence, they can be affiliated to the same sv. *viciae* despite their different chromosomal backgrounds. By contrast, diverse symbiotic genes can be harbored by strains belonging to the same species and even gs, as demonstrated in the *R. leguminosarum* species complex (Rlc), in which 3 sv. were distinguished^5,14,46^.

This phenomenon for *R. leguminosarum* has recently been described by Young and others. Based on concatenated sequences of 120 core genes and calculated pairwise average nucleotide identity (ANI) between 429 Rlc genomes, the researchers concluded that Rlc includes 18 distinct gs and 7 unique strains not grouped with these gs^14^ (gsC—147 strains, E—79, B—45, A—38, N and R—12 each, D, O, Q—8 each, M and K—6 each, H and I—5 each, L, G, S—3 each, J and P—2 each). Using the *nod* sequences present in their genomes, these Rlc strains were placed in one of the three sv. *trifolii*, *viciae*, or *phaseoli*. Interestingly, our results and those published by other researchers demonstrated that several distinct Rlc gs coexist at one site, and the same gs are found in various regions largely differing of local environmental conditions. For example, gsA was found in Australia, Greece, India, the USA, Russia, Norway, and Poland, gsB in Greece, Germany, China, and Peru, gsC in Australia, gsD in the USA, and gsE in Russia, Italy, the USA, Peru, and Ethiopia^14,32,76–79^.

The isolates used in this study were previously analyzed to estimate their genetic diversity using MLSA^46^. Given the results of phylogenetic analysis based on the concatenated sequences of five house-keeping genes (*atpD, recA, rpoB, gyrB, glnII*), the isolates were classified into four Rlc gs: A, B, E, and K (Table 5)^46^. Gs A, B, and E were numerous among the *R. leguminosarum* strains, whereas gsK was very rare (to date, only 6 strains, including our 3, have been classified into this gs). On the basis of the *nodC* and *nifH* sequences, all the strains analyzed in this study were assigned to the sv. *trifolii* (Figs. 6, 7). They were grouped in both phylogenetic trees with representative strains belonging to the sv. *trifolii* of Rlc gs A, B, C, E, H, and K (Figs. 6, 7, Table 4). Some of the reference Rlc strains used for this comparative analysis of symbiotic genes were grouped with rhizobia of the sv. *viciae* and *phaseoli*. For example, all the currently identified Rlc strains from gsK, i.e. our isolates KW1-9, KW2-9, and 5-8, as well as *R. leguminosarum* JHI2451, *R. leguminosarum* JHI54, and *R. leguminosarum* FA23 demonstrated very similar concatenated *atpD-glnII-gyrB-recA-rpoB* sequences^46^ but diverse *nodC* sequences (Fig. 6). The results obtained here are in congruence with the data provided by other researchers showing a clear discrepancy between the evolutionary histories of core and symbiotic genes^2–4,7,14,21,67^, as it was observed here that the strains from the same MLSA clade (Rlc gsK) were placed in distinct positions in the *nodC* phylogenetic tree. The strains KW1-9, KW2-9, and 5-8 isolated from *T. pratense* root nodules were grouped with the other *Trifolium* spp. root nodule isolates and with *R. leguminosarum* sv. *viciae* JHI245 and *R. leguminosarum* sv. *viciae* JHI54 isolated from root nodules of *Pisum sativum* and *Vicia sativa*, respectively. These reference strains were placed among the other strains of the symbiovar *viciae* (Fig. 6). These data are in congruence with other studies showing that *nodC* is a very good symbiotic marker that used together with other symbiotic genes ensures successful classification of *R. leguminosarum* strains into particular symbiovars^14,21,53,67–75^.

**Table 5:** Bacterial strains used in this study.

Strains	Characteristics	Sources
R1, R23, R32, R41, R51, R53, R70, R118, R137 (gs A); R13, R26, R49, R56, R66, R108 (gs B)	Strains derived from nodules of red clover plants grown in the subpolar climate region (Norway, Tromsø region, 69° 38′ 36–40′′ N, 18° 54′ 00–01′′ E)	^46^
2-2, 3-1, 3-3, 4-3, 6-11, M2, M14, M16, M19 (gs B); 8-3, 10-3, 24.2 (gs E); 5-8, KW1-9, KW2-9 (gs K)	Strains derived from nodules of red clover plants grown in the temperate climate region (Poland, Lublin region, 51° 15′ 55–57′′ N, 22° 32′ 6–10′′ E)	^46^

However, our results obtained from the comparative analysis of the *nodC* gene sequences and clades formed in the *nodC* phylogram did not provide conclusive evidence that there are *nodC* genotypes unique to all isolates from the particular climate region, although more than half of them (i.e., 10 of the 16 temperate and 9 of the 15 subpolar strains) were grouped according to their geographic origin (Fig. 6). All the studied isolates formed a common cluster with reference *R. leguminosarum* sv. *trifolii* strains isolated from root nodules of *T. pratense* grown in the temperate climate region, i.e. strains RCAM1365 and 9B from central Russia (Moscow)^8,10^ and strain 24.2 from Poland^11^. The other reference *R. leguminosarum* sv. *trifolii* strains (i.e., ATCC 14,479 originally isolated in Virginia (USA), strains TA1, WSM78, WSM409, and WSM1325 isolated from *T. subterraneum* in Tasmania (Australia), Macedonia, Sardinia (Italy), and Greece, respectively^12–14^, and CC275e isolated from *T. repens* in Australia) formed small subclusters or individual branches separated from the clade encompassing the temperate and subpolar *T. pratense* isolates (Fig. 6). The only exception was the strain M19, whose *nodC* gene sequence was 100% similar to *R. leguminosarum* sv. *trifolii* CC275e. Similarly, M19 was placed as a single strain clearly separated from the other *T. pratense* isolates on the phylogeny *nifH* tree (Fig. 7). Based on this analysis, all of the studied strains were classified to the sv. *trifolii* together with the reference *R. leguminosarum* sv. *trifolii* strains and clearly separated from the strains of the *viciae* and *phaseoli* sv. Furthermore, the analyzed strains were grouped into two large clusters, encompassing only the temperate strains (2-2, KW1-9, 4-3, 6-11, 8-3, 8-11, 10-3, KW2-9, M2, M16) and the strains from both climatic zones (R108, R13, R56, R26, 3-3, 3-1, R70, M14, R32, R49, R137, R41, R66, R118, R1, R23, R51, R53, 5-8). However, the reference *R. leguminosarum* sv. *trifolii* strains from the other geographic regions were not grouped in one common cluster in this tree (as shown in the *nodC* tree), suggesting that the *nifH* analysis cannot provide such information. M19 was placed distantly from all of the studied *T. pratense* isolates in both phylogeny trees constructed on the basis of the symbiotic genes, but it was grouped together with these strains in the *atpD-glnII-gyrB-recA-rpoB* tree^46^. This can be explained by the finding that this strain possesses two unique alleles for both these symbiotic genes (VIII for *nodC* and VIII for *nifH*, respectively) (Table 5).

In conclusion, our data indicate that *R. leguminosarum* sv. *trifolii* strains derived from two distinct climatic zones show a high diversity in their symbiotic efficiency with the host plant *T. pratense* under different temperature conditions and in the symbiotic heterogeneity estimated on the basis of the *nodC-nifH* genotypes. Some of these strains exhibit very good symbiotic performance in a wide range of temperatures and can therefore be a promising material for future studies and improvement of legume crop production.

## Conclusions

*Trifolium pratense* is a forage legume cultivated worldwide. This plant is able to establish a N-fixing symbiosis with soil bacteria belonging to *R. leguminosarum* sv. *trifolii*. So far, no comparative analysis of the symbiotic efficiency of *T. pratense* microsymbionts derived from two geographic regions, essentially differing in the temperature conditions, has been performed. In this study, we have characterized the symbiotic potential and heterogeneity of representative strains of two populations originating from the subpolar and temperate climate zones in a wide range of temperatures. The data obtained indicate that the symbiotic efficiency of individual *R. leguminosarum* sv. *trifolii* strains in red clover yielding is associated with the temperature conditions. However, some of the studied strains from both populations were characterized by high symbiotic efficiency in a wide range of temperatures. Furthermore, our data indicate that *R. leguminosarum* sv. *trifolii* strains derived from the subpolar region are little more efficient in clover root nodule formation at low temperatures (10–15 °C) than the temperate strains. However, some temperate strains also exhibit high nodulation efficiency in low temperature conditions, which suggests high adaptability of rhizobial strains to this abiotic stress factor.

Moreover, high symbiotic heterogeneity of the studied *R. leguminosarum* sv. *trifolii* strains was confirmed. In total, twenty *nodC-nifH* genotypes were identified among the tested rhizobial strains. Types A, B, and C proved to be the most frequent and mainly occurred in strains belonging to gs B and A, which are dominant gs among all the hitherto characterized *R. leguminosarum* strains. The unique *nodC-nifH* genotypes were identified in the strains that belonged to gs A, B, K, and E. They were identified in 9 temperate and 7 subpolar strains, indicating a similar frequency of their occurrence within the two examined populations. Interestingly, strains 5-8, KW1-9, and KW2-9, classified to the very rare gs K, also possess unique *nodC-nifH* genotypes (E, F, M). Taken together, our data show high symbiotic efficiency in biomass production and adaptability to low temperature stress of the red clover microsymbionts derived from different climate and geographic regions.

## Methods

### Rhizobial strains and growth conditions

The *R. leguminosarum* sv. *trifolii* strains used in this study are listed in Table 5. As described earlier in our study^46^, the strains were isolated from root nodules of red clover plants sampled from two European regions (Poland, Lublin region, 51° 15′ 55–57′′ N, 22° 32′ 6–10′′ E and Norway, Tromsø region, 69° 38′ 36–40′′ N, 18° 54′ 00–01′′ E). In total, 31 strains (i.e., 15 and 16 strains from the subpolar and temperate climate regions, respectively) were examined. The strains were grown in modified 79CA medium (containing 1 g of yeast extract, 1 g of casein hydrolysate, 0.5 g of K_2_HPO_4_, 0.1 g of NaCl, 0.1 g Ca glicerophosphate, 0.2 g of MgSO_4_ × 7H_2_O in 1 L, 1% of glycerol as a carbon source, pH 7.2). To determine growth rate of the strains in a wide range of temperatures (10–25 °C), 5 ml of 79CA medium in glass tubes was inoculated with bacteria to obtain an optical density OD_600_ = 0.1. The cultures were grown at 10, 15, 20 and 25 °C during 0–96 h in a rotary shaker (160 rpm), and the culture OD_600_ was measured after each 24 h. The experiment was done in duplicate (i.e. two cultures per strain and temperature tested were used).

### Plant experiments

The nodulation capability and other symbiotic properties of the strains were tested using commercially available seeds of *T. pratense* (L.). The influence of temperature on the symbiotic parameters was determined at 10, 15, 20, and 25 °C. For these experiments, clover seeds were surface-sterilized as described earlier^6^ and incubated for 48 h at 25 °C on N-free Fåhraeus agar plates^47^. Then, the seedlings were transferred into glass tubes containing 10 ml of Fåhraeus agar (one seedling per tube) and grown for 4 days in a plant growth chamber (25 °C, 80% humidity). After this time, the seedlings were inoculated with bacterial suspensions of OD_600_ = 0.2 (100 µl aliquot per seedling) and grown for 5 weeks (a 14 h day cycle with 3800 lumens and a 10 h night cycle without light). The dynamics of both root infection and nodule formation were determined after each week using magnifying glass. 5 weeks plants were harvested, and the length of shoots and roots as well as their wet masses were determined. The experiment was repeated two times using 20 plants for each strain and temperature variant. Since results obtained from two experiments were very similar, we presented data for the second experiment (with the exception of root infection effectiveness, where data from two experiments were analyzed together). Statement for plant material: our study complies with relevant institutional, national, and international guidelines and legislation.

### Determination of nitrogenase activity

The nitrogenase activity was determined using an acetylene reduction assay^80^ and clover plants grown at 10 °C and 20 °C. For this assay, 5 weeks plants were transferred into 20 ml glass vials closed with a rubber plug, and 1 ml of acetylene was added into each vial with the use of a syringe. Next, the samples were incubated for 1 h at the respective (10 °C or 20 °C) temperatures in the growth chamber (with light). The sample resolution was performed at 180 °C for 14 min in a gas chromatograph Clarus 500 equipped with a flame ionization detector (Perkin Elmer, USA) and a Carbosphere 80/100 packed column (Grace, USA). Three samples for each strain and temperature variant were analyzed. The amount of acetylene reduced to ethylene by nitrogenase in the nodules was calculated as µmol of produced C_2_H_4_ per g of fresh plant weight and per g of fresh shoot weight, respectively.

### Determination of N concentration in dry clover shoots

For determination of the %N in clover, shoots from 5-week plants were dried overnight at 80 °C and ground into powder using a grinder. 0.5 g of the plant material was used for this analysis. Two samples for each strain and temperature tested were analyzed. Mineralization of the samples was performed by transformation of organic N compounds into NH_4_SO_4_ using 98% H_2_SO_4_ in the presence of a Cu catalyzer and alkalization of obtained solutions in the Tecator Digestor Auto 20 apparatus (FOSS, USA) at a temperature of 420 °C for 1 h. Next, distillation and titration were performed in an automatic analyzer KjelRoc (Opsis) using HCl.

### DNA isolation and sequence analysis of *nodC* and *nifH* genes

The total DNA required for PCR reactions was isolated using 5 ml overnight bacterial cultures grown at 25 °C and the guanidium thiocyanate extraction method^46^. The concentration and purity of the DNA samples were determined in a Nanodrop 2000/2000c device (Thermo Scientific, USA). To obtain *nodC* and *nifH* sequences, PCR reactions were performed using oligonucleotide primers listed in Table 6 and a T-48 Personal thermocycler (Biometra, Germany). For *nodC*, a 548-bp fragment was amplified and sequenced using primers NodC1 and NodC2 and the following conditions: initial denaturation at 94 °C for 4 min; 35 cycles: 1 min at 94 °C, 40 s at 50 °C, 1 min at 72 °C; followed by a final 5 min elongation step at 72 °C. For *nifH*, a 747 bp fragment was obtained using Nif1 and Nif2 primers and amplification conditions identical as for *nodC*, with the exception of the annealing temperature (53 °C). PCR was carried out in a total volume of 100 µl. The mixtures contained 5 µl of template DNA (100 ng/µl), 1 µl of each primer (10 pmol/µl), 50 µl polymerase reaction buffer (ReadyMix Taq kit, Sigma-Aldrich, USA), and 43 µl of milli-Q water. The PCR amplicons obtained were purified using a Clean-up kit (A&A Biotechnology, Poland) and sequenced using the BigDye terminator Cycle Sequencing kit and the 3500 Genetic Analyzer (Applied Biosystems, USA). The sequences obtained for the *nodC* and *nifH* genes were deposited in the GenBank database under accession numbers ON745315-ON745344 and ON745375-ON745404, respectively, and are now publicly available.

**Table 6:** Oligonucleotide primers used in this study.

Primer	Sequence (5′–3′)	Target gene	References
NodC1	CGCGCCGCGCAGGGACGCTTC	*nodC*	^49^
NodC2	AGTAAAAGAAAGACGTTCACGAGCGTG	*nodC*	^49^
NifH1	CCGAAATCGAGAAGCATGTCCTC	*nifH*	^81^
NifH2	GCGTCAGATCGCATTCTATGGAAA	*nifH*	^81^

### Construction of phylogenetic trees

For phylogenetic analyses, the nucleotide sequences of *nodC* and *nifH* were compared with those obtained from the National Center for Biotechnology Information (NCBI) database using the BLASTN program^82^. Then, the sequences of the examined strains and the sequences available in the databases were aligned using the ClustalX software^83^ and corrected manually using GeneDoc^84,85^. The phylogenetic trees of the individual genes were constructed with the Maximum-Likelihood (ML) method using the best DNA substitution model determined in MEGAX (MEGAX software package)^86^. The phylogenetic distances for the *nodC* and *nifH* genes were calculated according to the Tamura-Nei + I + G model^87^. The reliability of tree topologies was estimated by a bootstrap confidence analysis based on 1000 resamplings^87^.

### Statistical analyses

Statistical analyses were performed using TIBCO Statistica version 13.3 (TIBCO Sofware Inc, US). The data are reported as the mean ± standard deviation (SD). Statistical significance was assumed at a *p* value of < 0.05. Comparisons of the effects of the temperature, time, and type of strains on the nodule formation on clover roots and the dynamics of plant root infection were performed using three-way ANOVA, and comparisons were made using the Holm-Sidak method. Before analysis, the normality of data distribution in each population was examined using the Shapiro–Wilk test. The uniformity of the variance was checked using the Brown-Forsythe test. Plant fresh weight and length, %N in dry shoots, and nitrogenase activity for each strain at the particular temperatures were analyzed by two-way ANOVA and Tukey’s post hoc test. One-way ANOVA was used for the analysis of bacterial growth rates. Normality of data distribution was tested using the Shapiro–Wilk test. Significant differences between the two populations tested at the individual temperatures or time points tested were determined using Student’s t-test (*p* < 0.05).

### Ethics approval

This article does not contain any studies with human participants and/or animals performed by any of the authors. The formal consent is not required in this study.

### Statement for plant material

Our study complies with relevant institutional, national, and international guidelines and legislation. Commercially available seeds of the *T. pratense* plants were used to test the strains in closed conditions (as it was incubation chambers).

### Supplementary Information

Supplementary Tables.

## Data Availability

All sequence data that support the findings of this study have been deposited in GenBank (https://www.ncbi.nlm.nih.gov/genbank/) with Accession Numbers ON745315-ON745344 and ON745375-ON745404 and are now publicly available.
