# Supplementary material for: Symbiotic efficiency of Rhizobium leguminosarum sv. trifolii strains originating from the subpolar and temperate climate regions
Source: Sci Rep. 2024 Mar 15;14:6264. doi: 10.1038/s41598-024-56988-1 (PMC10943007; doi:10.1038/s41598-024-56988-1)
Supplement: Supplementary file 1 — Supplementary Tables. [file 41598_2024_56988_MOESM1_ESM.docx]

SUPPLEMENTARY MATERIAL

**Symbiotic efficiency of Rhizobium leguminosarum sv. trifolii strains originating from the subpolar and temperate climate regions**

Monika Janczarek ^1^*, Marta Kozieł ^1^, Paulina Adamczyk ^­­­­1^, Katarzyna Buczek ^1^, Michał Kalita ^2^, Anna Gromada ^1^, Aleksandra Mordzińska-Rak ^3^, Cezary Polakowski ^4^, Andrzej Bieganowski ^4^

^1^ Department of Industrial and Environmental Microbiology, Institute of Biological Sciences, Faculty of Biology and Biotechnology, Maria Curie-Skłodowska University, 19 Akademicka, 20-033 Lublin, Poland; Monika Janczarek monika.janczarek@mail.umcs.pl Marta Kozieł martakozielbiot@gmail.com; Paulina Adamczyk [paulina.adamczyk@mail.umcs.pl](mailto:paulina.adamczyk@mail.umcs.pl); Katarzyna Buczek [romanczuk.kat@gmail.com](mailto:romanczuk.kat@gmail.com); Anna Gromada anna.gromada@mail.umcs.pl

^2^ Department of Genetics and Microbiology, Institute of Biological Sciences, Faculty of Biology and Biotechnology, Maria Curie-Skłodowska University, 19 Akademicka, 20-033 Lublin, Poland; [michal.kalita@mail.umcs.pl](mailto:michal.kalita@mail.umcs.pl);

^3^ Department of Biochemistry and Molecular Biology, Faculty of Medical Studies, Medical University in Lublin, 1 Chodźki, 20-093 Lublin, Poland; olam98@gmail.com

^4^ [Department of Natural Environment Biogeochemistry](https://www.ipan.lublin.pl/zaklady/zaklad-biogeochemii-srodowiska-przyrodniczego/), Institute of Agrophysics, Polish Academy of Sciences, 4 Doświadczalna, 20-290 Lublin, Poland; Cezary Polakowski [c.polakowski@ipan.lublin.pl](mailto:c.polakowski@ipan.lublin.pl), Andrzej Bieganowski [a.bieganowski@ipan.lublin.pl](mailto:a.bieganowski@ipan.lublin.pl)

***** Correspondence: monika.janczarek@mail.umcs.pl; Tel.: +48 81 5375909

***** *Correspondence to:*

Monika Janczarek, E-mail: [monika.janczarek@mail.umcs.pl](mailto:monika.janczarek@mail.umcs.pl)

ORCID identifier: 0000-0002-2250-6358

**Table S1.** Kinetics of infection of red clover roots by *R. leguminosarum* sv. *trifolii* strains from the

temperate climate collection examined at a wide range of temperatures (10-25°C).

| **Strain** | **Temperature (°C)** | **Kinetics of clover root infection observed as % of plants with root nodules (dpi)** | | | | |
| --- | --- | --- | --- | --- | --- | --- |
|  |  | **7** | **14** | **21** | **28** | **35** |
| 2-2 | 10 | 0±0^(a)^ | 0±0 ^(a)*^ | 25±5^(a)*^ | 70±10 ^(a)*^ | 75±5 ^(a)*^ |
|  | 15 | 0±0 ^(a)^ | 0±0 ^(a)*^ | 40±10 ^(a)*^ | 65±10 ^(a)*^ | 90±10 ^(ab)^ |
|  | 20 | 55±5 ^(b)^ | 100±0 ^(b)*^ | 100±0 ^(b)^ | 100±0 ^(b)^ | 100±0 ^(b)^ |
|  | 25 | 95±5 ^(c)^ | 100±0 ^(b)^ | 100±0 ^(b)^ | 100±0 ^(b)^ | 100±0 ^(b)^ |
| 3-1 | 10 | 0±0 ^(a)^ | 10±0 ^(a)^ | 75±5 ^(a)*^ | 85±5 ^(a)*^ | 90±0 ^(a)*^ |
|  | 15 | 0±0 ^(a)^ | 60±10 ^(b)*^ | 90±0 ^(ab)*^ | 100±0 ^(a)*^ | 100±0 ^(a)^ |
|  | 20 | 65±10 ^(b)^ | 100±0 ^(c)*^ | 100±0 ^(b)^ | 100±0 ^(a)^ | 100±0 ^(a)^ |
|  | 25 | 75±10 ^(b)*^ | 100±0 ^(c)^ | 100±0 ^(b)^ | 100±0 ^(a)^ | 100±0 ^(a)^ |
| 3-3 | 10 | 0±0 ^(a)^ | 15±0 ^(a)*^ | 20±0 ^(a)*^ | 55±5 ^(a)*^ | 80±10 ^(a)*^ |
|  | 15 | 0±0 ^(a)^ | 15±0 ^(a)*^ | 30±0 ^(a)*^ | 80±10 ^(b)*^ | 100±0 ^(a)^ |
|  | 20 | 45±10 ^(b)*^ | 100±0 ^(b)*^ | 100±0 ^(b)^ | 100±0 ^(b)^ | 100±0 ^(a)^ |
|  | 25 | 90±0 ^(b)^ | 100±0 ^(b)^ | 100±0 ^(b)^ | 100±0 ^(b)^ | 100±0 ^(a)^ |
| 4-3 | 10 | 0±0 ^(a)^ | 15±0 ^(a)*^ | 65±5 ^(a)*^ | 85±5 ^(a)*^ | 100±0 ^(a)*^ |
|  | 15 | 0±0 ^(a)^ | 50±10 ^(b)*^ | 80±10 ^(ab)*^ | 100±0 ^(a)*^ | 100±0 ^(a)^ |
|  | 20 | 65±10 ^(b)^ | 100±0 ^(c)*^ | 100±0 ^(b)^ | 100±0 ^(a)^ | 100±0 ^(a)^ |
|  | 25 | 100±0 ^(c)*^ | 100±0 ^(c)^ | 100±0 ^(b)^ | 100±0 ^(a)^ | 100±0 ^(a)^ |
| 5-8 | 10 | 0±0 ^(a)^ | 0±0 ^(a)*^ | 80±10 ^(a)*^ | 95±5 ^(a)*^ | 100±0 ^(a)*^ |
|  | 15 | 0±0 ^(a)^ | 40±10 ^(b)*^ | 85±5 ^(a)*^ | 100±0 ^(a)*^ | 100±0 ^(a)^ |
|  | 20 | 45±5 ^(b)*^ | 90±0 ^(c)*^ | 95±0 ^(a)^ | 100±0 ^(a)^ | 100±0 ^(a)^ |
|  | 25 | 100±0 ^c)*^ | 100±0 ^(c)^ | 100±0 ^(a)^ | 100±0 ^(a)^ | 100±0 ^(a)^ |
| 6-11 | 10 | 0±0 ^(a)^ | 0±0 ^(a)*^ | 20±0 ^(a)*^ | 45±5^(a)*^ | 55±5 ^(a)*^ |
|  | 15 | 0±0 ^(a)^ | 15±5 ^(a)*^ | 30±10^(a)*^ | 60±10 ^(b)*^ | 80±5 ^(b)^ |
|  | 20 | 40±20 ^(b)^ | 100±0 ^(b)*^ | 100±0 ^(b)^ | 100±0 ^(c)^ | 100±0 ^(c)^ |
|  | 25 | 80±10 ^(c)^ | 100±0 ^(b)^ | 100±0 ^(b)^ | 100±0 ^(c)^ | 100±0 ^(c)^ |
| 8-3 | 10 | 0±0 ^(a)^ | 0±0 ^(a)*^ | 30±5 ^(a)*^ | 95±5 ^(a)*^ | 100±0 ^(a)*^ |
|  | 15 | 0±0 ^(a)^ | 10±5 ^(a)*^ | 60±10 ^(b)*^ | 100±0 ^(a)*^ | 100±0 ^(a)^ |
|  | 20 | 45±5 ^(b)*^ | 95±5 ^(b)*^ | 95±5 ^(c)^ | 100±0 ^(a)^ | 100±0 ^(a)^ |
|  | 25 | 90±10 ^(c)^ | 100±0 ^(b)^ | 100±0 ^(c)^ | 100±0 ^(a)^ | 100±0 ^(a)^ |
| 8-11 | 10 | 0±0 ^(a)^ | 0±0 ^(a)*^ | 50±10 ^(a)*^ | 75±10 ^(a)*^ | 95±5 ^(a)*^ |
|  | 15 | 0±0 ^(a)^ | 35±5 ^(a)*^ | 95±5 ^(b)*^ | 100±0 ^(b)^ | 100±0 ^(a)^ |
|  | 20 | 65±5 ^(b*)^ | 100±0 ^(b)*^ | 100±0 ^(b)^ | 100±0 ^(b)*^ | 100±0 ^(a)^ |
|  | 25 | 90±10 ^(c)^ | 100±0 ^(b)^ | 100±0 ^(b)^ | 100±0 ^(b)^ | 100±0 ^(a)^ |
| 10-3 | 10 | 0±0 ^(a)^ | 0±0 ^(a)*^ | 65±15 ^(a)*^ | 100±0 ^(a)*^ | 100±0 ^(a)*^ |
|  | 15 | 0±0 ^(a)^ | 10±0 ^(a)*^ | 55±10 ^(a)*^ | 95±5 ^(a)*^ | 95±5 ^(a)^ |
|  | 20 | 70±10 ^(b)*^ | 100±0 ^(b)*^ | 100±0 ^(b)^ | 100±0 ^(a)^ | 100±0 ^(a)^ |
|  | 25 | 100±0 ^(c)*^ | 100±0 ^(b)^ | 100±0 ^(b)^ | 100±0 ^(a)^ | 100±0 ^(a)^ |
| KW1-9 | 10 | 0±0 ^(a)^ | 15±5 ^(a)*^ | 70±10 ^(a)*^ | 80±10 ^(a)*^ | 80±0 ^(a)^ |
|  | 15 | 0±0 ^(a)^ | 60±10 ^(b)*^ | 100±0 ^(b)*^ | 100±0 ^(a)*^ | 100±0 ^(a)^ |
|  | 20 | 55±15 ^(b)^ | 90±5 ^(bc)^ | 100±0 ^(b)^ | 100±0 ^(a)^ | 100±0 ^(a)^ |
|  | 25 | 95±5 ^(c)^ | 100±0 ^(c)^ | 100±0 ^(b)^ | 100±0 ^(a)^ | 100±0 ^(a)^ |
| KW2-9 | 10 | 0±0 ^(a)^ | 15±5 ^(a)*^ | 90±5^(a)*^ | 95±5 ^(a)*^ | 95±5 ^(a)^ |
|  | 15 | 0±0 ^(a)^ | 30±5 ^(a)*^ | 90±0 ^(a)*^ | 100±0 ^(a)*^ | 100±0 ^(a)^ |
|  | 20 | 40±10 ^(b)*^ | 95±5 ^(b)*^ | 100±0 ^(a)^ | 100±0 ^(a)^ | 100±0 ^(a)^ |
|  | 25 | 100±0 ^(c)*^ | 100±0 ^(b)^ | 100±0 ^(a)^ | 100±0 ^(a)^ | 100±0 ^(a)^ |
| M2 | 10 | 0±0 ^(a)^ | 0±0 ^(a)*^ | 65±15 ^(a)*^ | 90±10 ^(a)*^ | 100±0 ^(a)*^ |
|  | 15 | 0±0 ^(a)^ | 60±10 ^(b)*^ | 100±0 ^(b)*^ | 100±0 ^(a)*^ | 100±0 ^(a)^ |
|  | 20 | 65±15 ^(b)^ | 95±5 ^(c)*^ | 100±0 ^(b)^ | 100±0 ^(a)^ | 100±0 ^(a)^ |
|  | 25 | 100±0 ^(c)*^ | 100±0 ^(c)^ | 100±0 ^(b)^ | 100±0 ^(a)^ | 100±0 ^(a)^ |
| M14 | 10 | 0±0 ^(a)^ | 10±0 ^(a)*^ | 75±10 ^(a)*^ | 95±5 ^(a)*^ | 100±0 ^(a)*^ |
|  | 15 | 0±0 ^(a)^ | 10±0 ^(a)*^ | 90±10 ^(ab)*^ | 100±0 ^(a)*^ | 100±0 ^(a)^ |
|  | 20 | 60±10 ^(b)^ | 100±0 ^(b)*^ | 100±0 ^(b)^ | 100±0 ^(a)^ | 100±0 ^(a)^ |
|  | 25 | 90±5 ^(c)^ | 100±0 ^(b)^ | 100±0 ^(b)^ | 100±0 ^(a)^ | 100±0 ^(a)^ |
| M16 | 10 | 0±0 ^(a)^ | 10±5 ^(a)^ | 60±5 ^(a)*^ | 100±0 ^(a)*^ | 100±0 ^(a)*^ |
|  | 15 | 0±0 ^(a)^ | 65±10 ^(b)*^ | 95±5 ^(b)*^ | 100±0 ^(a)*^ | 100±0 ^(a)^ |
|  | 20 | 50±10 ^(b)^ | 95±5 ^(c)*^ | 100±0 ^(b)^ | 100±0 ^(a)^ | 100±0 ^(a)^ |
|  | 25 | 100±0 ^(c)*^ | 100±0 ^(c)^ | 100±0 ^(b)^ | 100±0 ^(a)^ | 100±0 ^(a)^ |
| M19 | 10 | 0±0 ^(a)^ | 20±10 ^(a)*^ | 75±10 ^(a)*^ | 100±0 ^(a)*^ | 100±0 ^(a)*^ |
|  | 15 | 0±0 ^(a)^ | 30±15 ^(a)*^ | 95±5 ^(ab)*^ | 100±0 ^(a)*^ | 100±0 ^(a)^ |
|  | 20 | 65±15 ^(b)^ | 100±0 ^(b)*^ | 100±0 ^(b)^ | 100±0 ^(a)^ | 100±0 ^(a)^ |
|  | 25 | 100±0 ^(c)*^ | 100±0 ^(b)^ | 100±0 ^(b)^ | 100±0 ^(a)^ | 100±0 ^(a)^ |
| 24-2 | 10 | 0±0 ^(a)^ | 0±0 ^(a)*^ | 60±10 ^(a)*^ | 75±5 ^(a)*^ | 100±0 ^(a)*^ |
|  | 15 | 0±0 ^(a)^ | 35±10 ^(a)*^ | 90±5 ^(b)*^ | 100±0 ^(b)*^ | 100±0 ^(a)^ |
|  | 20 | 45±10 ^(b)*^ | 100±0 ^(b)*^ | 100±0 ^(b)^ | 100±0 ^(b)^ | 100±0 ^(a)^ |
|  | 25 | 100±0 ^(c)*^ | 100±0 ^(b)^ | 100±0 ^(b)^ | 100±0 ^(b)^ | 100±0 ^(a)^ |

Data are presented as mean ± SD. Lower case letters in brackets indicate statistically significant differences (p≤0.05) for each individual strain tested at different temperatures at the same time point (dpi); ANOVA, Tukey’s post hoc test; * indicates statistically significant differences (p≤0.05) between the strains tested at the particular temperature and time point (dpi).

| **Comparison between strains** | **p-value** | **Comparison between strains** | **p- value** | **Comparison between strains** | **p-value** | **Comparison between strains** | **p-value** |
| --- | --- | --- | --- | --- | --- | --- | --- |
| **7 dpi 20°C** |  |  |  |  |  |  |  |
| 3-3 vs. 10-3 | 0.002 | 5-8 vs. 8-11 | 0.002 | 5-8 vs. 10-3 | 0.003 | 8-3 vs. 8-11 | 0.002 |
| KW2-9 vs. 8-11 | 0.001 | KW2-9 vs. 10-3 | 0.003 | 24.2 vs. 10-3 | 0.002 | 24.2 vs. 8-11 | 0.003 |
| **7 dpi 25°C** |  |  |  |  |  |  |  |
| 3-1 vs. 4-3 | 0.003 | 3-1 vs. 24.2 | 0.003 | 3-1 vs. KW2-9 | 0.003 | 3-1 vs. M16 | 0.003 |
| 3-1 vs. 5-8 | 0.003 | 3-1 vs. 10-3 | 0.003 | 3-1 vs. M2 | 0.003 | 3-1 vs. M19 | 0.003 |
| **14 dpi 10°C** |  |  |  |  |  |  |  |
| 2-2 vs. 3-3 | 0.008 | 5-8 vs. 3-3 | 0.008 | 6-11 vs. 3-3 | 0.008 | 8-3 vs. 3-3 | 0.008 |
| 2-2 vs. 4-3 | 0.008 | 5-8 vs. 4-3 | 0.008 | 6-11 vs. 4-3 | 0.008 | 8-3 vs. 4-3 | 0.008 |
| 2-2 vs. KW1-9 | 0.008 | 5-8 vs. KW1-9 | 0.008 | 6-11 vs. KW1-9 | 0.008 | 8-3 vs. KW1-9 | 0.008 |
| 2-2 KW2-9 | 0.008 | 5-8 KW2-9 | 0.008 | 6-11 vs. KW2-9 | 0.008 | 8-3 KW2-9 | 0.008 |
| 2-2 vs. M19 | 0.003 | 5-8 vs. M19 | 0.003 | 6-11 vs. M19 | 0.003 | 8-3 vs. M19 | 0.003 |
| 8-11 vs. 3-3 | 0.008 | 10-3 vs. 3-3 | 0.008 | M2 vs. 3-3 | 0.008 | 24.2 vs. 3-3 | 0.008 |
| 8-11 vs. 4-3 | 0.008 | 10-3 vs. 4-3 | 0.008 | M2 vs. 4-3 | 0.008 | 24.2 vs. 4-3 | 0.008 |
| 8-11 vs. KW1-9 | 0.008 | 10-3 vs. KW1-9 | 0.008 | M2 vs. KW1-9 | 0.008 | 24.2 vs. KW1-9 | 0.008 |
| 8-11 vs. KW2-9 | 0.008 | 10-3 vs. KW2-9 | 0.008 | M2 KW2-9 | 0.008 | 24.2 KW2-9 | 0.008 |
| 8-11 vs. M19 | 0.003 | 10-3 vs.M19 | 0.003 | M2 vs. M19 | 0.003 | 24.2 vs. M19 | 0.003 |
| **14 dpi 15°C** |  |  |  |  |  |  |  |
| 2-2 vs. 3-1 | <0.001 | 8-3 vs. M2 | <0.001 | KW2-9 vs. KW1-9 | <0.001 | 8-3 vs. 8-11 | 0.003 |
| 2-2 vs. M2 | <0.001 | 8-3 vs. 3-1 | <0.001 | KW2-9 vs. M2 | <0.001 | 8-3 vs. 5-8 | 0.001 |
| 2-2 vs. M16 | <0.001 | 8-3 vs. 4-3 | <0.001 | KW2-9 vs. M16 | <0.001 | 10-3 vs. 5-8 | 0.002 |
| 2-2 vs. 8-11 | <0.001 | 8-3 vs. M16 | <0.001 | M14 vs. 3-1 | <0.001 | M14 vs. M19 | 0.004 |
| 2-2 vs. KW1-9 | <0.001 | 8-3 vs. KW1-9 | <0.001 | M14 vs. 8-11 | 0.001 | 2-2 vs. 3-3 | 0.006 |
| 6-11 vs. 3-1 | <0.001 | 10-3 vs. 3-1 | <0.001 | M14 vs. 24.2 | 0.002 | 8-3 vs. 24.2 | 0.006 |
| 6-11 vs. KW1-9 | <0.001 | 10-3 vs. 4-3 | <0.001 | 3-3 vs. KW2-9 | 0.002 | 8-11 vs. 3-1 | 0.006 |
| 6-11 vs. 4-3 | <0.001 | 10-3 vs. KW1-9 | <0.001 | 2-2 vs. M19 | 0.002 | 8-11 vs. KW1-9 | 0.006 |
| 6-11 vs. M2 | <0.001 | 10-3 vs. M2 | <0.001 | 3-3 vs. 8-11 | 0.003 | 8-11 vs. M2 | 0.006 |
| 6-11 vs. M16 | <0.001 | 10-3 vs. M16 | <0.001 | 2-2 vs. 24.2 | 0.003 | 2-2 vs. 6-11 | 0.012 |
| 3-3 vs. 3-1 | <0.001 | 3-3 vs. M16 | <0.001 | 2-2 vs. KW2-9 | 0.003 | 3-3 vs. 24.2 | 0.006 |
| 3-3 vs. KW1-9 | <0.001 | M14 vs. M16 | <0.001 | 3-3 vs. M19 | 0.004 |  |  |
| 3-3 vs. M2 | <0.001 | M14 vs. M2 | <0.001 | KW2-9 vs. 3-1 | 0.004 |  |  |
| M14 vs. 5-8 | <0.001 | M14 vs. 4-3 | <0.001 | 8-11 vs. M16 | 0.004 |  |  |
| **14 dpi 20°C** |  |  |  |  |  |  |  |
| 5-8 vs. 2-2 | 0.012 | 5-8 vs. 3-3 | 0.012 | 5-8 vs. 6-11 | 0.012 | 5-8 vs. 10-3 | 0.012 |
| 5.8 vs. 3-1 | 0.012 | 5-8 vs. 4-3 | 0.012 | 5-8 vs. 8-11 | 0.012 | 5-8 vs. M14 | 0.012 |
| 5-8 vs. M19 | 0.012 | 5-8 vs. 24.2 | 0.012 | 5-8 vs. M16 | 0.012 | 5-8 vs. M2 | 0.012 |
| **21 dpi 10°C** |  |  |  |  |  |  |  |
| 2-2 vs. 3-1 | <0.001 | 3-3 vs. 4-3 | <0.001 | 6-11 vs. M19 | <0.001 | 8-3 vs. KW1-9 | 0.002 |
| 2-2 vs. 4-3 | <0.001 | 3-3 vs. 5-8 | <0.001 | 6-11 vs. 24.2 | <0.001 | 8-3 vs. M14 | 0.002 |
| 2-2 vs. 5-8 | <0.001 | 3-3 vs. KW1-9 | <0.001 | 6-11 vs. 5-8 | <0.001 | 8-3 vs. M16 | 0.004 |
| 2-2 vs. KW1-9 | <0.001 | 3-3 vs. M16 | <0.001 | 6-11 vs. KW1-9 | <0.001 | 8-3 vs. 24.2 | 0.004 |
| 2-2 vs. KW2-9 | <0.001 | 3-3 vs. M2 | <0.001 | 6-11 vs. KW2-9 | <0.001 | 8-3 vs. 10-3 | 0.006 |
| 2-2 vs. M2 | <0.001 | 3-3 vs. M14 | <0.001 | 6-11 vs. M2 | <0.001 | 8-3 vs. M2 | 0.006 |
| 2-2 vs. M14 | <0.001 | 3-3 vs. KW2-9 | <0.001 | 6-11 vs. M14 | <0.001 | 2-2 vs. 8-11 | 0.012 |
| 2-2 vs. M16 | <0.001 | 3-3 vs. M19 | <0.001 | 6-11 vs. M16 | <0.001 | 3-3 vs. 8-11 | 0.012 |
| 2-2 vs. M19 | <0.001 | 3-3 vs. 24.2 | <0.001 | 8-3 vs. 3-1 | <0.001 | 6-11 vs. 8-11 | 0.012 |
| 2-2 vs. 24.2 | <0.001 | 6-11 vs. 3-1 | <0.001 | 8-3 vs. 4-3 | <0.001 | 6-11 vs. 8-11 | 0.006 |
| 3-3 vs. 3-1 | <0.001 | 6-11 vs. 4-3 | <0.001 | 8-3 vs. 5-8 | <0.001 | 8-11 vs. 3-3 | 0.018 |
| 8-3 vs. KW2-9 | <0.001 | 8-3 vs. M19 | <0.001 |  |  |  |  |
| **21 dpi 15°C** |  |  |  |  |  |  |  |
| 2-2 vs. 3-1 | <0.001 | 3-3 vs. 3-1 | <0.001 | 3-3 vs. 4-3 | <0.001 | 2-2 vs. 4-3 | 0.004 |
| 2-2 vs. 24.2 | <0.001 | 3-3 vs. 8-11 | <0.001 | 3-3 vs. 5-8 | <0.001 | 3-3 vs. 10-3 | 0.006 |
| 2-2 vs. 5-8 | <0.001 | 6-11 vs. 3-1 | <0.001 | 3-3 vs. KW1-9 | <0.001 | 8-3 vs. 3-1 | 0.004 |
| 2-2 vs. KW1-9 | <0.001 | 6-11 vs. 4-3 | <0.001 | 3-3 vs. M16 | <0.001 | 8-3 vs. 8-11 | 0.004 |
| 2-2 vs. KW2-9 | <0.001 | 6-11 vs. M19 | <0.001 | 3-3 vs. M2 | <0.001 | 8-3 vs. KW2-9 | 0.004 |
| 2-2 vs. M2 | <0.001 | 6-11 vs. 24.2 | <0.001 | 3-3 vs. M14 | <0.001 | 8-3 vs. M16 | 0.002 |
| 2-2 vs. M14 | <0.001 | 6-11 vs. 5-8 | <0.001 | 3-3 vs. KW2-9 | <0.001 | 8-3 vs. M19 | 0.002 |
| 2-2 vs. M16 | <0.001 | 6-11 vs. KW1-9 | <0.001 | 3-3 vs. M19 | <0.001 | 10-3 vs. 24.2 | 0.004 |
| 2-2 vs. M19 | <0.001 | 6-11 vs. KW2-9 | <0.001 | 3-3 vs. 24.2 | <0.001 | 10-3 vs. M16 | 0.004 |
| 6-11 vs. M14 | <0.001 | 6-11 vs. M2 | <0.001 | 6-11 vs. 8-11 | <0.001 | 10-3 vs. M19 | 0.004 |
| 6-11 vs. M16 | <0.001 | 8-3 vs. M2 | <0.001 | 8-3 vs. KW1-9 | <0.001 | 10-3 vs. 5-8 | 0.006 |
| 10-3 vs. KW2-9 | <0.001 | 10-3 vs. KW1-9 | <0.001 | 10-3 vs. 3-1 | 0.002 | 8-3 vs. 24.2 | 0.006 |
| 10-3 vs. M2 | <0.001 | 10-3 vs. 8-11 | 0.002 | 10-3 vs. 5-8 | 0.002 | 10-3 vs. M14 | 0.006 |
| **28 dpi 10°C** |  |  |  |  |  |  |  |
| 3-3 vs. 10-3 | <0.001 | 6-11 vs. KW1-9 | <0.001 | 2-2 vs. M16 | 0.004 | 6-11 vs. 2-2 | 0.012 |
| 6-11 vs. 3-1 | <0.001 | 6-11 vs. KW2-9 | <0.001 | 3-3 vs. 3-1 | 0.004 | 6-11 vs. M2 | 0.012 |
| 6-11 vs. 4-3 | <0.001 | 6-11 vs. M19 | 0.002 | 3-3 vs. 4-3 | 0.004 | 2-2 vs. 5-8 | 0.012 |
| 6-11 vs. 8-3 | <0.001 | 6-11 vs. M14 | 0.002 | 2-2 vs. M19 | 0.004 | 3-3 vs. KW1-9 | 0.012 |
| 6-11 vs. 10-3 | <0.001 | 6-11 vs. 24.2 | 0.006 | 6-11 vs. 8-11 | 0.006 | 3-3 vs. M19 | 0.022 |
| 6-11 vs. 5-8 | <0.001 | 3-3 vs. KW2-9 | 0.002 | 8-11 vs. 10-3 | 0.005 | 3-3 vs. M14 | 0.022 |
| 3-3 vs. 8-3 | 0.001 | 2-2 vs. 10-3 | 0.004 |  |  |  |  |
| **28 dpi 15°C** |  |  |  |  |  |  |  |
| 6-11 vs. 3-1 | <0.001 | 6-11 vs. M2 | <0.001 | 2-2 vs. M2 | 0.001 | 6-11 vs. 10-3 | 0.005 |
| 6-11 vs. 4-3 | <0.001 | 6-11 vs. M14 | <0.001 | 2-2 vs. M14 | 0.001 | 2-2 vs.4-3 | 0.002 |
| 6-11 vs. M19 | <0.001 | 6-11 vs. M16 | <0.001 | 2-2 vs. M16 | 0.001 | 2-2 vs. 8-3 | 0.002 |
| 6-11 vs. 24.2 | <0.001 | 6-11 vs. 8-3 | <0.001 | 2-2 vs. M19 | 0.001 | 2-2 vs. 8-11 | 0.003 |
| 6-11 vs. 5-8 | <0.001 | 6-11 vs. 8-11 | <0.001 | 2-2 vs. 3-1 | 0.002 | 2-2 vs. 10-3 | 0.005 |
| 6-11 vs. KW1-9 | <0.001 | 2-2 vs. 24.2 | 0.002 | 2-2 vs. KW1-9 | 0.002 |  |  |
| 6-11 vs. KW2-9 | <0.001 | 2-2 vs. 5-8 | 0.002 | 2-2 vs. KW2-9 | 0.002 |  |  |
| **35 dpi 10°C** |  |  |  |  |  |  |  |
| 6-11 vs. 3-1 | <0.001 | 6-11 vs. M2 | <0.001 | 24.2 vs. 4-3 | 0.004 | 24.2 vs. 8-3 | 0.004 |
| 6-11 vs. 4-3 | <0.001 | 6-11 vs. KW2-9 | <0.001 | 2-2 vs. 5-8 | 0.004 | 24.2 vs. 5-8 | 0.004 |
| 6-11 vs. M16 | <0.001 | 6-11 vs. M14 | <0.001 | 2-2 vs. 10-3 | 0.004 | 24.2 vs. 10-3 | 0.004 |
| 6-11 vs. 24.2 | <0.001 | 6-11 vs. 8-11 | 0.001 | 2-2 vs. M14 | 0.004 | 24.2 vs. M14 | 0.004 |
| 6-11 vs. 5-8 | <0.001 | 6-11 vs. KW1-9 | 0.004 | 2-2 vs. M16 | 0.004 | 24.2 vs. M16 | 0.004 |
| 6-11 vs. 8-3 | <0.001 | 2-2 vs. 4-3 | 0.004 | 2-2 vs. 8-11 | 0.012 | 6-11 vs. 2-2 | 0.013 |
| 6-11 vs. 10-3 | <0.001 | 2-2 vs. 8-3 | 0.004 | 2-2 vs. 3-1 | 0.012 |  |  |

**Table S2.** Kinetics of infection of red clover roots by *R. leguminosarum* sv. *trifolii* strains from the

subpolar climate collection examined at a wide range of temperatures (10-25°C)

| **Strain** | **Temperature (ºC)** | **Dynamics of plant root infection observed as % of plants with root nodules (dpi)** | | | | |
| --- | --- | --- | --- | --- | --- | --- |
|  |  | **7** | **14** | **21** | **28** | **35** |
| R1 | 10 | 0±0^(a)^ | 30±5^(a)*^ | 80±10 ^(a)*^ | 95±5 ^(a)*^ | 100±0 ^(a)^ |
|  | 15 | 0±0 ^(a)^ | 35±5 ^(a)*^ | 100±0 ^(a*)^ | 100±0 ^(a)^ | 100±0 ^(a)^ |
|  | 20 | 60±20 ^(b)*^ | 100±0 ^(b)^ | 100±0 ^(a)^ | 100±0 ^(a)^ | 100±0 ^(a)^ |
|  | 25 | 100±0 ^(c)*^ | 100±0 ^(b)^ | 100±0 ^(a)^ | 100±0 ^(a)^ | 100±0 ^(a)^ |
| R13 | 10 | 0±0 ^(a)^ | 35±5 ^(a)*^ | 95±5 ^(a)*^ | 100±0 ^(a)*^ | 100±0 ^(a)^ |
|  | 15 | 0±0 ^(a)^ | 70±10 ^(b)*^ | 100±0 ^(a)*^ | 100±0 ^(a)^ | 100±0 ^(a)^ |
|  | 20 | 80±10 ^(b)*^ | 100±0 ^(c)^ | 100±0 ^(a)^ | 100±0 ^(a)^ | 100±0 ^(a)^ |
|  | 25 | 95±5 ^(c)*^ | 100±0 ^(c)^ | 100±0 ^(a)^ | 100±0 ^(a)^ | 100±0 ^(a)^ |
| R23 | 10 | 0±0 ^(a)^ | 25±5 ^(a)*^ | 70±10 ^(a)*^ | 100±0 ^(a)*^ | 100±0 ^(a)^ |
|  | 15 | 0±0 ^(a)^ | 70±10 ^(b)*^ | 95±5 ^(b)*^ | 100±0 ^(a)^ | 100±0 ^(a)^ |
|  | 20 | 60±10 ^(b)*^ | 95±5 ^(c)^ | 100±0 ^(b)^ | 100±0 ^(a)^ | 100±0 ^(a)^ |
|  | 25 | 100±0 ^(c)*^ | 100±0 ^(c)^ | 100±0 ^(b)^ | 100±0 ^(a)^ | 100±0 ^(a)^ |
| R26 | 10 | 0±0 ^(a)^ | 10±0 ^(a)*^ | 95±5 ^(a)*^ | 95±5 ^(a)*^ | 100±0 ^(a)^ |
|  | 15 | 0±0 ^(a)^ | 40±5 ^(b)*^ | 100±0 ^(a)*^ | 100±0 ^(a)^ | 100±0 ^(a)^ |
|  | 20 | 55±5 ^(b)*^ | 100±0 ^(c)^ | 100±0 ^(a)^ | 100±0 ^(a)^ | 100±0 ^(a)^ |
|  | 25 | 95±5 ^(c)*^ | 95±5 ^(c)^ | 100±0 ^(a)^ | 100±0 ^(a)^ | 100±0 ^(a)^ |
| R32 | 10 | 0±0 ^(a)^ | 40±10^(a)*^ | 75±5 ^(a)*^ | 100±0 ^(a)*^ | 100±0 ^(a)^ |
|  | 15 | 0±0 ^(a)^ | 55±5 ^(b)*^ | 95±5 ^(b)*^ | 100±0 ^(a)^ | 100±0 ^(a)^ |
|  | 20 | 55±10 ^(b)^ | 100±0 ^(c)^ | 100±0 ^(b)^ | 100±0 ^(a)^ | 100±0 ^(a)^ |
|  | 25 | 95±5 ^(c)*^ | 100±0 ^(c)^ | 100±0 ^(b)^ | 100±0 ^(a)^ | 100±0 ^(a)^ |
| R41 | 10 | 0±0 ^(a)^ | 15±5 ^(a)*^ | 55±15 ^(a)*^ | 100±0 ^(a)*^ | 100±0 ^(a)^ |
|  | 15 | 0±0 ^(a)^ | 35±15 ^(a)*^ | 95±5 ^(b)*^ | 100±0 ^(a)^ | 100±0 ^(a)^ |
|  | 20 | 65±10 ^(b)^ | 100±0 ^(b)^ | 100±0 ^(b)^ | 100±0 ^(a)^ | 100±0 ^(a)^ |
|  | 25 | 95±5 ^(c)*^ | 100±0 ^(b)^ | 100±0 ^(b)^ | 100±0 ^(a)^ | 100±0 ^(a)^ |
| R49 | 10 | 0±0 ^(a)^ | 15±5 ^(a)*^ | 65±10 ^(a)*^ | 90±10 ^(a)^ | 95±5 ^(a)^ |
|  | 15 | 0±0 ^(a)^ | 50±10 ^(b)*^ | 100±0 ^(b)*^ | 100±0^a)^ | 100±0 ^(a)^ |
|  | 20 | 85±5 ^(b)*^ | 100±0 ^(c)^ | 100±0 ^(b)^ | 100±0 ^(a)^ | 100±0 ^(a)^ |
|  | 25 | 100±0 ^(b)*^ | 100±0 ^(c)^ | 100±0 ^(b)^ | 100±0 ^(a)^ | 100±0 ^(a)^ |
| R51 | 10 | 0±0 ^(a)^ | 0±0 ^(a) *^ | 60±15 ^(a)*^ | 95±5 ^(a)*^ | 95±5 ^(a)^ |
|  | 15 | 0±0 ^(a)^ | 60±10 ^(b)*^ | 90±10 ^(b)*^ | 90±10 ^(a)^ | 95±5 ^(a)^ |
|  | 20 | 50±10 ^(b)*^ | 95±5 ^(c)^ | 100±0 ^(b)^ | 100±0 ^(a)^ | 100±0 ^(a)^ |
|  | 25 | 95±5 ^(c)*^ | 100±0 ^(c)^ | 100±0 ^(b)^ | 100±0 ^(a)^ | 100±0 ^(a)^ |
| R53 | 10 | 0±0 ^(a)^ | 5±5 ^(a)*^ | 40±10 ^(a)*^ | 90±10 ^(a)^ | 90±5 ^(a)^ |
|  | 15 | 0±0 ^(a)^ | 10±5 ^(a)*^ | 60±10 ^(a)^ | 90±10 ^(a)^ | 100±0 ^(a)^ |
|  | 20 | 40±10 ^(b)*^ | 100±0 ^(b)^ | 100±0 ^(b)^ | 100±0 ^(a)^ | 100±0 ^(a)^ |
|  | 25 | 85±10 ^(c)*^ | 100±0 ^(b)^ | 100±0 ^(b)^ | 100±0 ^(a)^ | 100±0 ^(a)^ |
| R56 | 10 | 0±0 ^(a)^ | 0±0 ^(a) *^ | 40±10 ^(a)*^ | 80±10 ^(a)^ | 95±5 ^(a)^ |
|  | 15 | 0±0 ^(a)^ | 50±10 ^(b)*^ | 95±5 ^(b)*^ | 95±5 ^(a)^ | 100±0 ^(a)^ |
|  | 20 | 50±15 ^(b)*^ | 100±0 ^(c)^ | 100±0 ^(b)^ | 100±0 ^(a)^ | 100±0 ^(a)^ |
|  | 25 | 100±0 ^(c)*^ | 100±0 ^(c)^ | 100±0^b)^ | 100±0 ^(a)^ | 100±0 ^(a)^ |
| R66 | 10 | 0±0 ^(a)^ | 0±0 ^(a)*^ | 65±15 ^(a)*^ | 95±5 ^(a)*^ | 100±0 ^(a)^ |
|  | 15 | 0±0 ^(a)^ | 25±5 ^(b)*^ | 95±5 ^(b)*^ | 100±0 ^(a)^ | 100±0 ^(a)^ |
|  | 20 | 50±10 ^(b)^ | 90±10 ^(c)^ | 95±5 ^(b)^ | 100±0 ^(a)^ | 100±0 ^(a)^ |
|  | 25 | 55±15 ^(b)*^ | 90±10 ^(c)^ | 95±5 ^(b)^ | 100±0 ^(a)^ | 100±0 ^(a)^ |
| R70 | 10 | 0±0 ^(a)^ | 0±0 ^(a)*^ | 50±15 ^(a)*^ | 100±0 ^(a)*^ | 100±0 ^(a)^ |
|  | 15 | 0±0 ^(a)^ | 30±10 ^(b)*^ | 90±10 ^(b)*^ | 100±0 ^(a)^ | 100±0 ^(a)^ |
|  | 20 | 70±10 ^(b)*^ | 90±10 ^(c)^ | 100±0 ^(b)^ | 100±0 ^(a)^ | 100±0 ^(a)^ |
|  | 25 | 95±5 ^(c)*^ | 100±0 ^(c)^ | 100±0 ^(b)^ | 100±0 ^(a)^ | 100±0 ^(a)^ |
| R108 | 10 | 0±0 ^(a)^ | 10±5 ^(a)*^ | 75±10 ^(a)*^ | 95±5 ^(a)*^ | 100±0 ^(a)^ |
|  | 15 | 0±0 ^(a)^ | 35±10 ^(b)*^ | 85±15 ^(ab)^ | 100±0 ^(a)^ | 100±0 ^(a)^ |
|  | 20 | 50±10 ^(b)^ | 95±5 ^(c)^ | 100±0 ^(b)^ | 100±0 ^(a)^ | 100±0 ^(a)^ |
|  | 25 | 90±10 ^(c)*^ | 100±0 ^(c)^ | 100±0 ^(b)^ | 100±0 ^(a)^ | 100±0 ^(a)^ |
| R118 | 10 | 0±0 ^(a)^ | 0±0 ^(a)*^ | 25±5^(a)*^ | 65±15 ^(a)^ | 95±5 ^(a)^ |
|  | 15 | 0±0 ^(a)^ | 25±10 ^(b)*^ | 65±10 ^(b)^ | 90±10 ^(b)^ | 100±0 ^(a)^ |
|  | 20 | 70±10 ^(b)*^ | 90±10 ^(c)^ | 95±5 ^(c)^ | 95±5 ^(b)^ | 100±0 ^(a)^ |
|  | 25 | 100±0 ^(c)*^ | 100±0 ^(c)^ | 100±0 ^(c)^ | 100±0 ^(b)^ | 100±0 ^(a)^ |
| R137 | 10 | 0±0 ^(a)^ | 10±5 ^(a)*^ | 55±15 ^(a)*^ | 95±5 ^(a)*^ | 100±0 ^(a)^ |
|  | 15 | 0±0 ^(a)^ | 30±10 ^(a)*^ | 95±5 ^(b)*^ | 100±0 ^(a)^ | 100±0 ^(a)^ |
|  | 20 | 75±10 ^(b)*^ | 100±0 ^(b)^ | 100±0 ^(b)^ | 100±0 ^(a)^ | 100±0 ^(a)^ |
|  | 25 | 85±5 ^(b)*^ | 100±0 ^(b)^ | 100±0 ^(b)^ | 100±0 ^(a)^ | 100±0 ^(a)^ |

Data are presented as mean ± SD. Lower case letters in brackets indicate statistically significant differences (p≤0.05) for each individual strain tested at different temperatures at the same time point (dpi); ANOVA, Tukey’s post hoc test; * indicates statistically significant differences (p≤0.05) between the strains tested at the particular temperature and time point (dpi).

| **Comparison between strains** | **p-value** | **Comparison between strains** | **p-value** | **Comparison between strains** | **p-value** | **Comparison between strains** | **p-value** |
| --- | --- | --- | --- | --- | --- | --- | --- |
| **7 dpi 20°C** |  |  |  |  |  |  |  |
| R53 vs. R49 | 0.001 | R53 vs. R13 | 0.004 | R26 vs. R49 | 0.004 | R23 vs. R49 | 0.012 |
| R51 vs. R49 | 0.004 | R53 vs. R118 | 0.004 | R53 vs. R137 | 0.006 | R53 vs. R70 | 0.012 |
| **7 dpi 25°C** |  |  |  |  |  |  |  |
| R66 vs. R1 | 0.001 | R66 vs. R56 | 0.001 | R66 vs. R26 | 0.004 | R66 vs. R70 | 0.004 |
| R66 vs. R23 | 0.001 | R66 vs. R118 | 0.001 | R66 vs. R32 | 0.004 | R66 vs. R51 | 0.004 |
| R66 vs. R49 | 0.001 | R66 vs. R13 | 0.004 | R66 vs. R41 | 0.004 | R66 vs. R137 | 0.012 |
| **14 dpi 10°C** |  |  |  |  |  |  |  |
| R32 vs. R51 | <0.001 | R1 vs. R66 | 0.001 | R13 vs. R118 | 0.002 | R1 vs. R53 | 0.006 |
| R32 vs. R56 | <0.001 | R1 vs. R70 | 0.001 | R32 vs. R137 | 0.006 | R32 vs. R41 | 0.012 |
| R32 vs. R66 | <0.001 | R1 vs. R118 | 0.001 | R32 vs. R108 | 0.006 | R32 vs. R49 | 0.012 |
| R1 vs. R56 | 0.001 | R32 vs. R26 | 0.004 | R1 vs. R137 | 0.012 | R1 vs. R41 | 0.018 |
| R1 vs. R51 | 0.001 | R1 vs. R26 | 0.006 | R1 vs. R108 | 0.012 | R13 vs. R41 | 0.012 |
| R13 vs. R56 | 0.001 | R13 vs. R66 | 0.002 | R13 vs. R26 | 0.008 | R13 vs. R49 | 0.012 |
| R13 vs. R51 | 0.001 | R13 vs. R70 | 0.002 | R13 vs. R53 | 0.006 | R1 vs. R49 | 0.018 |
| **14 dpi 15°C** |  |  |  |  |  |  |  |
| R13 vs. R53 | <0.001 | R13 vs. R118 | 0.002 | R13 vs. R26 | 0.006 | R13 vs. R41 | 0.012 |
| R13 vs. R66 | <0.001 | R13 vs. R137 | 0.004 | R1 vs. R53 | 0.006 | R1 vs. R32 | 0.012 |
| R51 vs. R53 | <0.001 | R13 vs. R70 | 0.004 | R23 vs. R26 | 0.006 | R1 vs. R51 | 0.012 |
| R32 vs. R53 | <0.001 | R13 vs. R108 | 0.006 | R23 vs. R137 | 0.004 | R49 vs. R66 | 0.012 |
| R23 vs. R53 | <0.001 | R1 vs. R23 | 0.004 | R23 vs. R70 | 0.004 | R53 vs. R70 | 0.018 |
| R23 vs. R66 | <0.001 | R1 vs. R13 | 0.004 | R23 vs. R108 | 0.006 | R53 vs. R137 | 0.018 |
| R49 vs. R53 | 0.002 | R32 vs. R66 | 0.004 | R23 vs. R41 | 0.012 | R26 vs.R66 | 0.018 |
| R53 vs. R56 | 0.002 | R32 vs. R118 | 0.004 | R32 vs. R70 | 0.012 |  |  |
| R23 vs. R118 | 0.002 | R26 vs. R53 | 0.004 | R53 vs. R108 | 0.012 |  |  |
| **21 dpi 10°C** |  |  |  |  |  |  |  |
| R13 vs. R53 | <0.001 | R26 vs. R53 | <0.001 | R51 vs. R118 | 0.006 | R41 vs. R118 | 0.012 |
| R13 vs. R56 | <0.001 | R26 vs. R56 | <0.001 | R1 vs. R53 | 0.004 | R118 vs. R137 | 0.012 |
| R13 vs. R118 | <0.001 | R26 vs. R118 | <0.001 | R1 vs. R56 | 0.004 | R13 vs. R23 | 0.012 |
| R1 vs. R118 | <0.001 | R108 vs. R118 | <0.001 | R26 vs. R41 | 0.004 | R13 vs. R32 | 0.012 |
| R23 vs. R118 | <0.001 | R32 vs. R118 | <0.001 | R26 vs. R70 | 0.002 | R26 vs. R49 | 0.012 |
| R13 vs. R41 | 0.004 | R49 vs. R118 | 0.002 | R26 vs. R137 | 0.004 | R26 vs. R23 | 0.012 |
| R13 vs. R70 | 0.002 | R32 vs. R52 | 0.002 | R26 vs. R49 | 0.006 | R26 vs. R32 | 0.014 |
| R13 vs. R137 | 0.004 | R32 vs. R56 | 0.002 | R26 vs. R51 | 0.006 | R26 vs. R66 | 0.014 |
| R26 vs. R41 | 0.004 | R66 vs. R118 | 0.002 | R13 vs. R49 | 0.006 | R23 vs. R56 | 0.015 |
| R26 vs. R51 | 0.006 | R13 vs. R66 | 0.012 | R13 vs. R51 | 0.006 | R13 vs. R108 | 0.018 |
| R26 vs. R53 | 0.006 | R26 vs. R23 | 0.012 | R23 vs. R53 | 0.012 | R26 vs. R108 | 0.018 |
| **21 dpi 15°C** |  |  |  |  |  |  |  |
| R53 vs. R1 | 0.001 | R118 vs. R26 | 0.002 | R53 vs. R23 | 0.004 | R53 vs. R56 | 0.006 |
| R53 vs. R13 | 0.001 | R118 vs. R49 | 0.002 | R53 vs. R56 | 0.004 | R53 vs. R51 | 0.012 |
| R53 vs. R26 | 0.001 | R118 vs. R13 | 0.002 | R53 vs. R23 | 0.006 | R53 vs. R70 | 0.012 |
| R53 vs. R49 | 0.001 | R53 vs. R32 | 0.004 | R53 vs. R32 | 0.006 | R53 vs. R51 | 0.018 |
| R118 vs. R1 | 0.002 | R53 vs. R41 | 0.004 | R53 vs. R41 | 0.006 | R53 vs. R70 | 0.018 |
| **28 dpi 10°C** |  |  |  |  |  |  |  |
| R118 vs. R13 | 0.004 | R118 vs. R41 | 0.004 | R56 vs. R32 | 0.012 | R118 vs. R66 | 0.012 |
| R118 vs. R23 | 0.004 | R56 vs. R13 | 0.012 | R56 vs. R41 | 0.012 | R118 vs. R51 | 0.012 |
| R118 vs. R70 | 0.004 | R56 vs. R23 | 0.012 | R118 vs. R137 | 0.012 | R118 vs. R26 | 0.012 |
| R118 vs. R32 | 0.004 | R56 vs. R70 | 0.012 | R118 vs. R108 | 0.012 | R118 vs. R1 | 0.012 |

**Table S3.** Nodulation of red clover roots by *R. leguminosarum* sv. *trifolii* strains from the temperate climate population examined at a wide range of temperatures (10-25°C)

| **Strain** | | | **Temperature (°C)** | | **Average number of nodules per plant determined during 35-day experiment (dpi)** | | | | | | | | | | | | | | | |
| --- | --- | --- | --- | --- | --- | --- | --- | --- | --- | --- | --- | --- | --- | --- | --- | --- | --- | --- | --- | --- |
|  |  |  |  |  | **7** | | **14** | | | **21** | | **28** | | **35** | | | | | | |
| 2-2 | | | 10 | | 0±0^(a)^ | | 0±0 ^(a)^ | | | 0.25±0.15 ^(a)^ | | 0.9±0.31 ^(a)^ | | 1.15±0.41 ^(a)^ | | | | | | |
|  |  |  | 15 | | 0±0 ^(a)^ | | 0±0 ^(a)^ | | | 0.55±0.22 ^(a)^ | | 1.0±0.36 ^(a)^ | | 1.55±0.52 ^(a)^ | | | | | | |
|  |  |  | 20 | | 0.8±0.34 ^(a)^ | | 3.35±1.11 ^(b)^ | | | 6.1±1.82 ^(b)*^ | | 8.4±0.84 ^(b)*^ | | 9.45±1.55 ^(b)*^ | | | | | | |
|  |  |  | 25 | | 2.75±1.55 ^(a)^ | | 6.6±1.61 ^(c)^ | | | 8.55±2.21 ^(b)^ | | 11.2±1.62 ^(c)*^ | | 14.7±2.62 ^(c)*^ | | | | | | |
| 3-1 | | | 10 | | 0±0 ^(a)^ | | 0.15±0.05 ^(a)^ | | | 1.2±0.23 ^(a)^ | | 2.75±0.48 ^(a)^ | | 3.05±0.45 ^(a)^ | | | | | | |
|  |  |  | 15 | | 0±0 ^(a)^ | | 0.85±0.32 ^(a)^ | | | 1.75±0.51 ^(a)^ | | 3.3±0.56 ^(a)^ | | 3.7±071 ^(a)^ | | | | | | |
|  |  |  | 20 | | 1.0±0.3 ^(a)^ | | 4.75±1.1 ^(b)^ | | | 6.95±1.3 ^(b)*^ | | 9.4±1.3 ^(b)*^ | | 9.85±1.92 ^(b)*^ | | | | | | |
|  |  |  | 25 | | 1.4±0.5 ^(a)^ | | 5.0±1.3 ^(b)^ | | | 6.8±1.41 ^(b)*^ | | 9.5±1.41 ^(b)*^ | | 10.25±2.2 ^(b)*^ | | | | | | |
| 3-3 | | | 10 | | 0±0 ^(a)^ | | 0±0 ^(a)^ | | | 0.3±0.1 ^(a)^ | | 1.05±0.31 ^(a)^ | | 2.85±0.5 ^(a)^ | | | | | | |
|  |  |  | 15 | | 0±0 ^(a)^ | | 0.05±0.03 ^(a)^ | | | 0.45±0.18 ^(a)^ | | 1.6±0.42 ^(a)^ | | 2.9±0.43 ^(a)^ | | | | | | |
|  |  |  | 20 | | 0.5±0.35 ^(a)^ | | 3.35±0.72 ^(b)^ | | | 8.2±1.82 ^(b)*^ | | 9.85±2.2 ^(b)*^ | | 10.4±2.4 ^(b)*^ | | | | | | |
|  |  |  | 25 | | 2.75±1.9 ^(a)^ | | 5.85±1.83 ^(b)^ | | | 7.95±1.58 ^(b)^ | | 9.2±3.1 ^(b)*^ | | 10.75±2.7 ^(b)*^ | | | | | | |
| 4-3 | | | 10 | | 0±0 ^(a)^ | | 0.15±0.05 ^(a)^ | | | 1.3±0.25 ^(a)^ | | 2.0±0.36 ^(a)^ | | 3.1±0.7 ^(a)^ | | | | | | |
|  |  |  | 15 | | 0±0 ^(a)^ | | 0.6±0.13 ^(a)^ | | | 1.45±0.28 ^(a)^ | | 2.35±0.48 ^(a)^ | | 3.2±0.8 ^(a)^ | | | | | | |
|  |  |  | 20 | | 1.3±0.25 ^(a)^ | | 4.3±0.81 ^(b)^ | | | 7.1±1.32 ^(b)*^ | | 9.55±2.12 ^(b)*^ | | 11±2.1 ^(b)*^ | | | | | | |
|  |  |  | 25 | | 2.5±0.46 ^(a)^ | | 5.05±1.1 ^(b)^ | | | 6.75±1.24 ^(b)*^ | | 9.25±2.3 ^(b)*^ | | 11.5±2.4 ^(b)*^ | | | | | | |
| 5-8 | | | 10 | | 0±0 ^(a)^ | | 0±0 ^(a)^ | | | 1.5±0.7 ^(a)^ | | 2.95±0.56 ^(a)^ | | 3.7±0.72 ^(a)^ | | | | | | |
|  |  |  | 15 | | 0±0 ^(a)^ | | 0.55±0.21 ^(a)*^ | | | 1.5±0.65 ^(a)^ | | 3.05±0.61 ^(a)^ | | 4.5±0.8 ^(a)^ | | | | | | |
|  |  |  | 20 | | 0.5±0.2 ^(a)^ | | 2.05±1.4^(a)*^ | | | 3.95±0.9 ^(a)*^ | | 6.75±0.85 ^(b)*^ | | 8.75±1.34 ^(b*)^ | | | | | | |
|  |  |  | 25 | | 2.3±1.7 ^(a)^ | | 6.0±2.4 ^(b)^ | | | 7.9±2.2 ^(b)^ | | 9.55±1.58 ^(c)*^ | | 10.5±1.8 ^(b)*^ | | | | | | |
| 6-11 | | | 10 | | 0±0 ^(a)^ | | 0±0 ^(a)^ | | | 0.15±0.06 ^(a)^ | | 0.5±0.2 ^(a)^ | | 1.35±0.28 ^(a)^ | | | | | | |
|  |  |  | 15 | | 0±0 ^(a)^ | | 0.15±0.05 ^(a)^ | | | 0.45±0.13 ^(a)^ | | 1.5±0.85 ^(a)^ | | 1.95±0.5 ^(a)^ | | | | | | |
|  |  |  | 20 | | 0.55±0.22^(a)^ | | 4.15±0.7 ^(b)^ | | | 7.15±0.35 ^(b)*^ | | 9.35±2.05 ^(b)^ | | 10.7±2.1 ^(b)*^ | | | | | | |
|  |  |  | 25 | | 2.05±1.35^(a)^ | | 5.1±0.95 ^(b)^ | | | 6.65±0.4 ^(b)*^ | | 8.55±2.1 ^(b)*^ | | 10.15±2.3 ^(b)*^ | | | | | | |
| 8-3 | | | 10 | | 0±0 ^(a)^ | | 0±0 ^(a)^ | | | 0.35±0.11 ^(a)^ | | 1.85±0.55 ^(a)^ | | 3.65±0.62 ^(a)^ | | | | | | |
|  |  |  | 15 | | 0±0 ^(a)^ | | 0±0^(a)*^ | | | 0.75±0.3 ^(a)^ | | 2.45±0.6 ^(a)^ | | 3.85±0.8 ^(a)^ | | | | | | |
|  |  |  | 20 | | 0.9±0.35 ^(a)^ | | 2.3±0.5 ^(ab)^ | | | 4.1±1.3 ^(b)*^ | | 5.4±1.5 ^(b)^ | | 6.75±1.1 ^(b)*^ | | | | | | |
|  |  |  | 25 | | 2.1±0.93 ^(a)^ | | 3.75±0.95 ^(b)^ | | | 5.85±1.8 ^(b)*^ | | 7.5±2.2 ^(b)*^ | | 10.8±1.8 ^(c)*^ | | | | | | |
| 8-11 | | | 10 | | 0±0 ^(a)^ | | 0±0 ^(a)^ | | | 0.6±0.26 ^(a)^ | | 1.8±0.4 ^(a)^ | | 2.55±0.6 ^(a)^ | | | | | | |
|  |  |  | 15 | | 0±0 ^(a)^ | | 0.45±0.12^(a)*^ | | | 1.7±0.91 ^(a)^ | | 2.8±0.65 ^(a)^ | | 3.65±0.7 ^(a)^ | | | | | | |
|  |  |  | 20 | | 1.55±0.41 ^(a)^ | | 5.5±1.12 ^(b)^ | | | 7.65±1.78 ^(b*)^ | | 9.0±1.8 ^(b*)^ | | 9.35±1.7 ^(b)*^ | | | | | | |
|  |  |  | 25 | | 2.15±0.82 ^(a)^ | | 6.2±1.65 ^(b)^ | | | 7.4±1.62 ^(b)*^ | | 10.05±2.2 ^(b)*^ | | 11.75±2.6 ^(b)*^ | | | | | | |
| 10-3 | | | 10 | | 0±0 ^(a)^ | | 0.05±0.02 ^(a)^ | | | 1.05±0.3 ^(a)^ | | 1.9±0.41 ^(a)^ | | 2.35±0.42 ^(a)^ | | | | | | |
|  |  |  | 15 | | 0±0 ^(a)^ | | 0.1±0.03 ^(a)^ | | | 1.05±0.25 ^(a)^ | | 2.1±0.45 ^(a)^ | | 2.85±0.6 ^(a)^ | | | | | | |
|  |  |  | 20 | | 1.4±0.42 ^(a)^ | | 5.2±1.21 ^(b)^ | | | 10.6±2.1 ^(b)*^ | | 12.55±2.8 ^(b)*^ | | 14.7±3.2 ^(b)*^ | | | | | | |
|  |  |  | 25 | | 2.65±0.9 ^(a)^ | | 5.05±1.14 ^(b)^ | | | 8.55±1.65 ^(b)*^ | | 10.75±1.9 ^(b)*^ | | 11.95±2.8 ^(b)*^ | | | | | | |
| KW1-9 | | | 10 | | 0±0 ^(a)^ | | 0.2±0.05 ^(a)^ | | | 0.95±0.24 ^(a)^ | | 1.85±0.58 ^(a)^ | | 2.25±0.56 ^(a)^ | | | | | | |
|  |  |  | 15 | | 0±0 ^(a)^ | | 1.1±0.25 ^(a)^ | | | 2.65±0.56 ^(a)^ | | 4.05±1.11 ^(b)^ | | 4.3±0.92 ^(a)^ | | | | | | |
|  |  |  | 20 | | 0.75±0.44 ^(ab)^ | | 4.3±0.93 ^(b)^ | | | 7.6±1.63 ^(b)*^ | | 9.2±2.24 ^(c)*^ | | 10.05±2.15 ^(b)*^ | | | | | | |
|  |  |  | 25 | | 3.05±1.95 ^(b)^ | | 6.65±1.5 ^(b)^ | | | 7.35±1.9 ^(b)^ | | 9.3±2.31 ^(c)*^ | | 11.4±3.21 ^(b)*^ | | | | | | |
| KW2-9 | | | 10 | | 0±0 ^(a)^ | | 0.15±0.05 ^(a)^ | | | 1.65±0.4 ^(a)^ | | 3.05±0.56 ^(a)^ | | 3.65±0.82 ^(a)^ | | | | | | |
|  |  |  | 15 | | 0±0 ^(a)^ | | 0.35±0.11 ^(a)^ | | | 2.4±0.52 ^(a)^ | | 3.1±0.8 ^(a)^ | | 3.7±0.83 ^(a)^ | | | | | | |
|  |  |  | 20 | | 0.45±0.11 ^(ab)^ | | 3.0±1.32 ^(b)^ | | | 6.4±1.58 ^(b*)^ | | 8.0±1.12 ^(b)*^ | | 8.95±1.23 ^(b)*^ | | | | | | |
|  |  |  | 25 | | 2.85±0.61 ^(b)^ | | 6.1±1.81 ^(c)^ | | | 8.2±2.12 ^(b)*^ | | 11.4±1.54 ^(c)*^ | | 13.25±2.52 ^(c)*^ | | | | | | |
| M2 | | | 10 | | 0±0 ^(a)^ | | 0±0 ^(a)^ | | | 1.2±0.33 ^(a)^ | | 2.55±0.61 ^(a)^ | | 2.75±0.7 ^(a)^ | | | | | | |
|  |  |  | 15 | | 0±0 ^(a)^ | | 1.0±0.32 ^(a)^ | | | 2.0±0.38 ^(a)^ | | 2.75±0.72 ^(a)^ | | 3.65±0.87 ^(a)^ | | | | | | |
|  |  |  | 20 | | 1.45±0.9 ^(ab)^ | | 4.05±0.98 ^(b)^ | | | 6.45±1.43 ^(b)*^ | | 8.05±1.93 ^(b)*^ | | 8.6±2.12 ^(b)*^ | | | | | | |
|  |  |  | 25 | | 2.9±1.11 ^(b)^ | | 6.7±1.45 ^(b)^ | | | 8.25±2.1 ^(b)^ | | 10.3±2.4 ^(b)*^ | | 11.85±2.43 ^(c)*^ | | | | | | |
| M14 | | | 10 | | 0±0 ^(a)^ | | 0.1±0.04 ^(a)^ | | | 1.15±0.35 ^(a)^ | | 2.75±0.9 ^(a)^ | | 3.5±0.81 ^(a)^ | | | | | | |
|  |  |  | 15 | | 0±0 ^(a)^ | | 0.15±0.06 ^(a)^ | | | 1.15±0.4 ^(a)^ | | 2.1±0.56 ^(a)^ | | 3.55±0.77 ^(a)^ | | | | | | |
|  |  |  | 20 | | 1.05±0.42 ^(a)^ | | 4.45±1.15 ^(b)^ | | | 6.25±1.62 ^(b)*^ | | 7.55±1.62 ^(b)*^ | | 8.6±1.62 ^(b)*^ | | | | | | |
|  |  |  | 25 | | 2.3±0.98 ^(a)^ | | 7.15±1.85 ^(b)^ | | | 8.8±1.89 ^(b)^ | | 11.25±2.2 ^(c)*^ | | 13.35±2.45 ^(c)*^ | | | | | | |
| M16 | | | 10 | | 0±0 ^(a)^ | | 0.1±0.03 ^(a)^ | | | 1.15±0.38 ^(a)^ | | 2.6±0.52 ^(a)^ | | 3.4±0.9 ^(a)^ | | | | | | |
|  |  |  | 15 | | 0±0 ^(a)^ | | 0.9±0.28 ^(a)^ | | | 2.05±0.56 ^(a)^ | | 3.0±0.61 ^(a)^ | | 3.55±1.1 ^(a)^ | | | | | | |
|  |  |  | 20 | | 0.7±0.22 ^(a)^ | | 3.95±0.97 ^(b)^ | | | 7.4±1.46 ^(b)*^ | | 8.55±1.2 ^(b)*^ | | 9.7±2.75 ^(b)*^ | | | | | | |
|  |  |  | 25 | | 2.5±0.67 ^(b)^ | | 6.05±1.98 ^(b)^ | | | 7.8±1.67 ^(b)^ | | 11.5±1.86 ^(c)*^ | | 12.35±3.53 ^(b)*^ | | | | | | |
| M19 | | | 10 | | 0±0 ^(a)^ | | 0.25±0.08 ^(a)^ | | | 1.4±0.41 ^(a)^ | | 2.55±0.62 ^(a)^ | | 3.2±0.9 ^(a)^ | | | | | | |
|  |  |  | 15 | | 0±0 ^(a)^ | | 0.3±0.065 ^(a)^ | | | 1.45±0.39 ^(a)^ | | 2.3±0.77 ^(a)^ | | 3.25±0.82 ^(a)^ | | | | | | |
|  |  |  | 20 | | 1.1±0.28 ^(ab)^ | | 4.7±1.4 ^(b)^ | | | 7.55±1.52 ^(b)*^ | | 11.15±3.4 ^(b)*^ | | 13.2±3.84 ^(b)*^ | | | | | | |
|  |  |  | 25 | | 2.85±0.68 ^(b)^ | | 6.6±1.97 ^(b)^ | | | 9.55±2.18 ^(b)*^ | | 13.05±4.1 ^(b)*^ | | 16.25±4.16 ^(c)*^ | | | | | | |
| 24.2 | | | 10 | | 0±0 ^(a)^ | | 0±0 ^(a)^ | | | 1.1±0.35 ^(a)^ | | 2.05±0.76 ^(a)^ | | 2.7±0.78 ^(a)^ | | | | | | |
|  |  |  | 15 | | 0±0 ^(a)^ | | 0.55±0.16 ^(a)^ | | | 2.35±0.72 ^(a)^ | | 3.15±0.83 ^(a)^ | | 3.55±1.13 ^(a)^ | | | | | | |
|  |  |  | 20 | | 0.55±0.14 ^(a)^ | | 4.9±1.21 ^(b)^ | | | 11.2±3.2 ^(b)*^ | | 17.45±4.45 ^(b)*^ | | 19.3±5.1 ^(b)*^ | | | | | | |
|  |  |  | 25 | | 2.2±0.68 ^(a)^ | | 7.2±1.63 ^(b)^ | | | 10.75±2.83 ^(b)*^ | | 15.3±3.98 ^(b)*^ | | 15.85±4.11 ^(b)*^ | | | | | | |
|  |  | Data are presented as mean ± SD. Lower case letters in brackets indicate statistically significant differences (p≤0.05) for each individual strain tested at different temperatures at the same time point (dpi); ANOVA, Tukey’s post hoc test; * indicates statistically significant differences (p≤0.05) between the strains tested at the particular temperature and time point (dpi). | | | | | | | | | | | | |  |  | |  |  |  |
| **Comparison between strains** | | | | **p-value** | | **Comparison between strains** | | **p-value** | **Comparison between strains** | | **p-value** | | **Comparison between strains** | | | | **p-value** | | | |
| **14 dpi 15°C** | | | |  | |  | |  |  | |  | |  | | | |  | | | |
| 8-11 vs. 5-8 | | | | 0.015 | | 8-11 vs. 8-3 | | 0.044 |  | |  | |  | | | |  | | | |
| **21 dpi 20°C** | | | |  | |  | |  |  | |  | |  | | | |  | | | |
| 24.2 vs. 5-8 | | | | <0.001 | | 10-3 vs. M14 | | <0.001 | 8-11 vs. 5-8 | | 0.004 | | 10-3 vs. 4-3 | | | | 0.01 | | | |
| 24.2 vs. 8-3 | | | | <0.001 | | 24.2 vs. 3-1 | | <0.001 | 10-3 vs. 3-1 | | 0.005 | | M19 vs. 8-3 | | | | 0.012 | | | |
| 10-3 vs. 5-8 | | | | <0.001 | | 3-3 vs. 5-8 | | <0.001 | KW1-9 vs. 5-8 | | 0.005 | | 10-3 vs. 6-11 | | | | 0.012 | | | |
| 10-3 vs. 8-3 | | | | <0.001 | | 10-3 vs. KW2-9 | | <0.001 | 24.2 vs. M19 | | 0.005 | | M16 vs. 5-8 | | | | 0.012 | | | |
| 24.2 vs. 2-2 | | | | <0.001 | | 10-3 vs. M2 | | <0.001 | M19 vs. 5-8 | | 0.006 | | M16 vs. 8-3 | | | | 0.022 | | | |
| 24.2 vs. M14 | | | | <0.001 | | 3-3 vs. 8-3 | | <0.001 | 224.2 vs. KW1-9 | | 0.006 | | 10-3 vs. M16 | | | | 0.033 | | | |
| 24.2 vs. KW2-9 | | | | <0.001 | | 24.2 vs. 4-3 | | <0.001 | 8-11 vs. 8-3 | | 0.008 | | 6-11 vs. 5-8 | | | | 0.033 | | | |
| 24.2 vs. M2 | | | | <0.001 | | 24.2 vs. 6-11 | | <0.001 | 24.2 vs. 8-11 | | 0.008 | | 4-3 vs. 5-8 | | | | 0.04 | | | |
| 10-3 vs. 2-2 | | | | <0.001 | | 24.2 vs. M16 | | 0.003 | KW1-9 vs. 8-3 | | 0.009 | |  | | | |  | | | |
| **21 dpi 25°C** | | | |  | |  | |  |  | |  | |  | | | |  | | | |
| 24.2 vs. 8-3 | | | | <0.001 | | 24.2 vs. 6-11 | | <0.001 | 24.2 vs. 3-1 | | 0.001 | | 24.2 vs. KW1-9 | | | | 0.018 | | | |
| 24.2 vs. 10-3 | | | | <0.001 | | 24.2 vs. 4-3 | | 0.001 | M19 vs. 8-3 | | 0.05 | | 24.2 vs. 8-11 | | | | 0.022 | | | |
| **28 dpi 20°C** | | | |  | |  | |  |  | |  | |  | | | |  | | | |
| 24.2 vs. 8-3 | | | | <0.001 | | 10-3 vs. 5-8 | | <0.001 | M19 vs. 5-8 | | <0.001 | | 24.2 vs. 10-3 | | | | 0.001 | | | |
| 24.2 vs. 5-8 | | | | <0.001 | | 24.2 vs. M19 | | <0.001 | 10-3 vs. KW1-9 | | <0.001 | | KW1-9 vs. 8-3 | | | | 0.002 | | | |
| 24.2 vs. M14 | | | | <0.001 | | 10-3 vs. KW2-9 | | <0.001 | 10-3 vs. 6-11 | | <0.001 | | 10-3 vs. 3-3 | | | | 0.003 | | | |
| 24.2 vs. KW2-9 | | | | <0.001 | | 10-3 vs. M2 | | <0.001 | 4-3 vs. 8-3 | | <0.001 | | 8-11 vs. 8-3 | | | | 0.005 | | | |
| 24.2 vs. M2 | | | | <0.001 | | 10-3 vs. 2-2 | | <0.001 | 10-3 vs. 3-1 | | <0.001 | | M19 vs. M14 | | | | 0.005 | | | |
| 24.2 vs. 2-2 | | | | <0.001 | | 10-3 vs. M16 | | <0.001 | 10-3 vs. 4-3 | | <0.001 | | M19 vs. KW2-9 | | | | 0.038 | | | |
| 24.2 vs. M16 | | | | <0.001 | | 24.2 vs. 6-11 | | <0.001 | 10-3 vs. 8-3 | | <0.001 | | M16 vs. 8-3 | | | | 0.038 | | | |
| 24.2 vs. 8-11 | | | | <0.001 | | 24.2 vs. 3-1 | | <0.001 | 10-3 vs. 8-11 | | <0.001 | | M19 vs. M2 | | | | 0.046 | | | |
| 24.2 vs. KW1-9 | | | | <0.001 | | 24.2 vs. 4-3 | | <0.001 | 3-3 vs. 8-3 | | <0.001 | | 3-3 vs. 5-8 | | | | 0.045 | | | |
| 3-1 vs. 8-3 | | | | <0.001 | | 24.2 vs. 3-3 | | <0.001 | 6-11 vs. 8-3 | | 0.001 | |  | | | |  | | | |
| **28 dpi 25°C** | | | |  | |  | |  |  | |  | |  | | | |  | | | |
| 24.2 vs. 8-3 | | | | <0.001 | | 24.2 vs. 5-8 | | <0.001 | 24.2 vs. M14 | | <0.001 | | M19 vs. KW1-9 | | | | 0.003 | | | |
| 24.2 vs. 6-11 | | | | <0.001 | | 24.2 vs. 10-3 | | <0.001 | M16 vs. 8-3 | | <0.001 | | M14 vs. 8-3 | | | | 0.003 | | | |
| 24.2 vs. 3-3 | | | | <0.001 | | M19- vs. 8-3 | | <0.001 | 24.2 vs. KW2-9 | | 0.002 | | 2-2 vs. 8-3 | | | | 0.004 | | | |
| 24.2 vs. 4-3 | | | | <0.001 | | 24.2 vs. 8-11 | | <0.001 | KW2-9 vs. 8-3 | | 0.002 | | M19 vs. 3-1 | | | | 0.008 | | | |
| 24.2 vs. KW1-9 | | | | <0.001 | | 24.2 vs. M2 | | <0.001 | M19 vs. 3-3 | | 0.002 | | M19 vs. 5-8 | | | | 0.01 | | | |
| 24.2 vs. 3-1 | | | | <0.001 | | M19 vs. 6-11 | | <0.001 | 24.2 vs. M16 | | 0.002 | | M19 vs. 10-3 | | | | 0.023 | | | |
| 24.2 vs. 2-2 | | | | <0.001 | |  | |  |  | |  | |  | | | |  | | | |
| **35 dpi 20°C** | | | |  | |  | |  |  | |  | |  | | | |  | | | |
| 24.2 vs. 8-3 | | | | <0.001 | | 10-3 vs. 8-3 | | <0.001 | 10-3 vs. 8-11 | | <0.001 | | M19 vs. KW2-9 | | | | <0.001 | | | |
| 24.2 vs. M14 | | | | <0.001 | | 24.2 vs. 10-3 | | <0.001 | 10-3 vs. 2-2 | | <0.001 | | 6-11 vs. 8-3 | | | | 0.001 | | | |
| 24.2 vs. M2 | | | | <0.001 | | 10-3 vs. M14 | | <0.001 | 10-3 vs. M16 | | <0.001 | | M19 vs. 8-11 | | | | 0.002 | | | |
| 24.2 vs. 5-8 | | | | <0.001 | | 10-3 vs. M2 | | <0.001 | 10-3 vs. 3-1 | | <0.001 | | M19 vs. 2-2 | | | | 0.003 | | | |
| 24.2 vs. KW2-9 | | | | <0.001 | | 10-3 vs. 5-8 | | <0.001 | 10-3 vs. KW1-9 | | <0.001 | | 3-3 vs. 8-3 | | | | 0.004 | | | |
| 24.2 vs. 8-11 | | | | <0.001 | | 10-3 vs. KW2-9 | | <0.001 | 10-3 vs. 3-3 | | <0.001 | | M19 vs. M16 | | | | 0.008 | | | |
| 24.2 vs. 2-2 | | | | <0.001 | | M19 vs. 8-3 | | <0.001 | 10-3 vs. 6-11 | | <0.001 | | M19 vs. 3-1 | | | | 0.016 | | | |
| 24.2 vs. M16 | | | | <0.001 | | 24.2 vs. 3-3 | | <0.001 | 10-3 vs. 4-3 | | <0.001 | | KW1-9 vs. 8-3 | | | | 0.02 | | | |
| 24.2 vs. 3-1 | | | | <0.001 | | 24.2 vs. 6-11 | | <0.001 | M19 vs. M2 | | <0.001 | | M19 vs. KW1-9 | | | | 0.037 | | | |
| 24.2 vs. KW1-9 | | | | <0.001 | | 24.2 vs. 4-3 | | <0.001 | M19 vs. M14 | | <0.001 | | 3-1 vs. 8-3 | | | | 0.044 | | | |
| M19 vs. 5-8 | | | | <0.001 | | 24.2 vs. M19 | | <0.001 | 4-3 vs. 8-3 | | <0.001 | |  | | | |  | | | |
| **35 dpi 25°C** | | | |  | |  | |  |  | |  | |  | | | |  | | | |
| M19 vs. 6-11 | | | | <0.001 | | 24.2 vs. 10-3 | | <0.001 | 24.2 vs. 8-3 | | <0.001 | | 2-2 vs. 10-3 | | | | 0.003 | | | |
| M19 vs. 3-1 | | | | <0.001 | | M19 vs. KW1-9 | | <0.001 | 2-2 vs. 5-8 | | <0.001 | | 24.2 vs. M16 | | | | 0.009 | | | |
| M19 vs. 5-8 | | | | <0.001 | | M19 vs. M2 | | <0.001 | 24.2 vs. 8-11 | | <0.001 | | 2-2 vs. KW1-9 | | | | 0.023 | | | |
| 24.2 vs. 6-11 | | | | <0.001 | | 24.2 vs. 4-3 | | <0.001 | 24.2 vs. M2 | | <0.001 | | M14 vs. 6-11 | | | | 0.034 | | | |
| 24.2 vs. 3-1 | | | | <0.001 | | 24.2 vs. 5-8 | | <0.001 | 2-2 vs. 3-3 | | 0.001 | | 2-2 vs 4-3 | | | | 0.034 | | | |
| M19 vs. 3-3 | | | | <0.001 | | M19 vs. 10-3 | | <0.001 | 2-2 vs. 8-3 | | 0.001 | | KW2-9 vs. 6-11 | | | | 0.05 | | | |
| M19 vs. 8-3 | | | | <0.001 | | 24.2 vs. 3-3 | | <0.001 | M19 vs. M16 | | 0.001 | | M14 vs. 3-1 | | | | 0.05 | | | |

**Table S4.** Nodulation of red clover roots by *R. leguminosarum* sv. *trifolii* strains from the subpolar climate

population examined at a wide range of temperatures (10-25°C)

| **Strain** | | | **Temperature (ºC)** | | **Average number of nodules per plant determined during 35-day experiment (dpi)** | | | | | | | | | | | | | | |
| --- | --- | --- | --- | --- | --- | --- | --- | --- | --- | --- | --- | --- | --- | --- | --- | --- | --- | --- | --- |
|  |  |  |  |  | **7** | | **14** | | **21** | | **28** | | | **35** | | | | | |
| R1 | | | 10 | | 0±0^(a)^ | | 0.3±0.08 ^(a)^ | | 1.6±0.4 ^(a)^ | | 2.7±0.4 ^(a)^ | | | 4.25±0.6 ^(a)^ | | | | | |
|  |  |  | 15 | | 0±0 ^(a)^ | | 0.45±0.1 ^(a)^ | | 1.75±0.6 ^(a)^ | | 2.8±0.5 ^(a)^ | | | 4.55±0.5 ^(a)^ | | | | | |
|  |  |  | 20 | | 0.85±0.2 ^(ab)^ | | 4.2±0.85 ^(b)^ | | 8.8±1.8 ^(b)^ | | 11.5±1.1 ^(b)*^ | | | 13.3±1.3 ^(b)*^ | | | | | |
|  |  |  | 25 | | 2.95±0.75 ^(b)^ | | 6.9±1.2 ^(b)^ | | 10.25±2.2^(b)*^ | | 16.1±2.2 ^(c)*^ | | | 20.8±2.4 ^(c)*^ | | | | | |
| R13 | | | 10 | | 0±0 ^(a)^ | | 0.35±0.11 ^(a)^ | | 2.75±0.5 ^(a)^ | | 3.3±0.6 ^(a)^ | | | 4.0±0.9 ^(a)^ | | | | | |
|  |  |  | 15 | | 0±0 ^(a)^ | | 0.85±0.35 ^(a)^ | | 2.35±0.4 ^(a)^ | | 2.75±0.7 ^(a)^ | | | 3.8±0.8 ^(a)^ | | | | | |
|  |  |  | 20 | | 1.45±0.6 ^(a)^ | | 5.7±1.2 ^(b)^ | | 8.4±2.4 ^(b)^ | | 10.05±2.4^(b)*^ | | | 10.9±1.9 ^(b)*^ | | | | | |
|  |  |  | 25 | | 1.8±0.7 ^(a)^ | | 5.0±0.8 ^(b)^ | | 8.4±2.2 ^(b)^ | | 10.3±2.1 ^(b)*^ | | | 12.25±2.4 ^(b)*^ | | | | | |
| R23 | | | 10 | | 0±0 ^(a)^ | | 0.25±0.05 ^(a)^ | | 1.45±0.4 ^(a)^ | | 2.7±0.6^(a)^ | | | 2.9±0.6 ^(a)^ | | | | | |
|  |  |  | 15 | | 0±0 ^(a)^ | | 1.5±0.4^(a)^ | | 2.8±0.9^(a)^ | | 3.5±0.9 ^(a)^ | | | 4.25±0.95 ^(a)^ | | | | | |
|  |  |  | 20 | | 0.9±0.38^(ab)^ | | 2.55±0.58 ^(a)^ | | 9.45±1.5 ^(b)*^ | | 17.8±3.4 ^(b)*^ | | | 20.85±4.4 ^(b)*^ | | | | | |
|  |  |  | 25 | | 2.9±0.9 ^(b)^ | | 5.95±0.9 ^(b)^ | | 7.9±1.4 ^(b)^ | | 12.45±3.2^(b)*^ | | | 16.2±3.7 ^(b)*^ | | | | | |
| R26 | | | 10 | | 0±0 ^(a)^ | | 0.1±0.04 ^(a)^ | | 2.25±0.4 ^(a)^ | | 3.15±0.6 ^(a)^ | | | 4.8±1.3 ^(a)^ | | | | | |
|  |  |  | 15 | | 0±0 ^(a)^ | | 0.45±0.1 ^(ab)^ | | 2.3±0.5 ^(a)^ | | 3.2±0.7 ^(a)^ | | | 4.9±1.4 ^(a)^ | | | | | |
|  |  |  | 20 | | 0.7±0.25 ^(ab)^ | | 3.05±0.6 ^(b)^ | | 5.85±1.2 ^(b)^ | | 7.95±2.4 ^(b)*^ | | | 8.4±2.2 ^(b)*^ | | | | | |
|  |  |  | 25 | | 3.45±0.5 ^(b)^ | | 5.35±1.8 ^(bc)^ | | 8.3±2.1 ^(b)^ | | 10.3±3.2 ^(b)*^ | | | 14.8±3.1 ^(c)*^ | | | | | |
| R32 | | | 10 | | 0±0 ^(a)^ | | 0.4±0.15 ^(a)^ | | 1.75±0.4 ^(a)^ | | 3.25±0.7 ^(a)^ | | | 4.1±0.7 ^(a)^ | | | | | |
|  |  |  | 15 | | 0±0 ^(a)^ | | 0.5±0.17 ^(a)^ | | 1.6±0.35 ^(a)^ | | 2.35±0.55 ^(a)^ | | | 3.15±0.8 ^(a)^ | | | | | |
|  |  |  | 20 | | 0.8±0.3 ^(a)^ | | 2.85±0.6 ^(ab)^ | | 5.35±0.8 ^(b)*^ | | 7.75±1.0 ^(b)*^ | | | 7.9±1.5 ^(b)*^ | | | | | |
|  |  |  | 25 | | 1.9±0.9 ^(a)^ | | 3.6±0.7 ^(b)^ | | 6.5±0.9 ^(b)*^ | | 10.65±1.1 ^(c)*^ | | | 12.35±2.4 ^(c)*^ | | | | | |
| R41 | | | 10 | | 0±0 ^(a)^ | | 0.25±0.05 ^(a)^ | | 1.15±0.3 ^(a)^ | | 3.3±0.7 ^(a)^ | | | 3.75±0.7 ^(a)^ | | | | | |
|  |  |  | 15 | | 0±0 ^(a)^ | | 0.45±0.1 ^(a)^ | | 1.95±0.4 ^(a)^ | | 3.1±0.6 ^(a)^ | | | 4.45±0.6 ^(a)^ | | | | | |
|  |  |  | 20 | | 0.3±0.07 ^(a)^ | | 2.55±0.5 ^(ab)^ | | 6.25±0.6 ^(b)^ | | 9.7±0.8 ^(b)*^ | | | 11.65±2.7 ^(b)*^ | | | | | |
|  |  |  | 25 | | 2.05±0.4 ^(a)^ | | 4.45±0.4 ^(b)^ | | 7.55±1.2 ^(b)^ | | 13.05±1.5^(c)*^ | | | 18.0±4.1 ^(c)*^ | | | | | |
| R49 | | | 10 | | 0±0 ^(a)^ | | 0.2±0.08 ^(a)^ | | 1.55±0.4 ^(a)^ | | 2.5±0.6 ^(a)^ | | | 3.75±0.9 ^(a)^ | | | | | |
|  |  |  | 15 | | 0±0 ^(a)^ | | 0.6±0.2 ^(a)^ | | 1.7±0.5 ^(a)^ | | 3.0±0.7 ^(a)^ | | | 3.9±0.8 ^(a)^ | | | | | |
|  |  |  | 20 | | 1.4±0.4 ^(a)^ | | 2.65±0.8 ^(a)^ | | 5.35±1.1 ^(b)*^ | | 7.65±1.7 ^(b)*^ | | | 8.9±2.6 ^(b)*^ | | | | | |
|  |  |  | 25 | | 2.6±0.8 ^(a)^ | | 7.45±1.9 ^(b)^ | | 9.6±2.4 ^(c)^ | | 13.05±2.7 ^(c)*^ | | | 16.35±4.1 ^(c)*^ | | | | | |
| R51 | | | 10 | | 0±0 ^(a)^ | | 0±0 ^(a)^ | | 1.15±0.35 ^(a)^ | | 2.7±0.8 ^(a)^ | | | 3.3±0.8 ^(a)^ | | | | | |
|  |  |  | 15 | | 0±0 ^(a)^ | | 0.9±0.3 ^(ab)^ | | 2.1±0.45 ^(a)^ | | 2.75±0.75 ^(a)^ | | | 3.45±0.76 ^(a)^ | | | | | |
|  |  |  | 20 | | 0.75±0.25 ^(a)^ | | 3.75±0.75 ^(b)^ | | 7.55±1.7 ^(b)^ | | 12.2±3.1 ^(b)*^ | | | 14.1±1.3 ^(b)*^ | | | | | |
|  |  |  | 25 | | 2.05±0.7 ^(a)^ | | 5.6±2.2 ^(bc)^ | | 8.6±2.2 ^(b)^ | | 13.3±3.4 ^(b)*^ | | | 17.4±2.6 ^(b)*^ | | | | | |
| R53 | | | 10 | | 0±0 ^(a)^ | | 0.05±0.15^(a)^ | | 0.55±0.15^(a)^ | | 1.7±0.5 ^(a)^ | | | 2.2±0.5 ^(a)^ | | | | | |
|  |  |  | 15 | | 0±0 ^(a)^ | | 0.15±0.1 ^(a)^ | | 0.65±1.4 ^(a)^ | | 1.95±0.4 ^(a)^ | | | 3.05±0.6 ^(a)^ | | | | | |
|  |  |  | 20 | | 0.55±0.15 ^(a)^ | | 4.4±1.1 ^(b)^ | | 9.15±2.2 ^(b)^ | | 12.9±1.5 ^(b)*^ | | | 15.3±2.3 ^(b)*^ | | | | | |
|  |  |  | 25 | | 2±0.4 ^(a)^ | | 6.15±1.4 ^(b)^ | | 10.7±2.4 ^(b)*^ | | 16.8±2.1 ^(c)*^ | | | 22±3.1 ^(c)*^ | | | | | |
| R56 | | | 10 | | 0±0 ^(a)^ | | 0±0 ^(a)^ | | 0.65±0.3 ^(a)^ | | 1.25±0.5 ^(a)^ | | | 2.0±0.4 ^(a)^ | | | | | |
|  |  |  | 15 | | 0±0 ^(a)^ | | 0.85±0.3^(ab)^ | | 2.7±0.6 ^(a)^ | | 3.2±0.6 ^(a)^ | | | 3.95±1.1 ^(a)^ | | | | | |
|  |  |  | 20 | | 0.7±0.25 ^(a)^ | | 3.2±0.7 ^(b)^ | | 5.8±1.4 ^(b)^ | | 8.0±1.9 ^(b)*^ | | | 9.8±2.1 ^(b)*^ | | | | | |
|  |  |  | 25 | | 2.55±0.9 ^(a)^ | | 5.1±1.1 ^(bc)^ | | 7.0±01.9^(b)*^ | | 9.7±2.2 ^(b)*^ | | | 10.75±2.4 ^(b)*^ | | | | | |
| R66 | | | 10 | | 0±0 ^(a)^ | | 0±0 ^(a)^ | | 1.6±0.46^(a)^ | | 2.65±0.7 ^(a)^ | | | 3.0±0.9 ^(a)^ | | | | | |
|  |  |  | 15 | | 0±0 ^(a)^ | | 0.35±0.15 ^(a)^ | | 1.7±0.35 ^(a)^ | | 2.8±0.6 ^(a)^ | | | 3.7±1.1 ^(a)^ | | | | | |
|  |  |  | 20 | | 0.7±0.3^(a)^ | | 2.8±0.6 ^(a)^ | | 7.05±1.4 ^(b)^ | | 12.3±3.2 ^(b)*^ | | | 16.5±4.4 ^(b)*^ | | | | | |
|  |  |  | 25 | | 2.8±0.8 ^(a)^ | | 6.1±1.1^(b)^ | | 9.4±1.9^(b)^ | | 13.6±3.1 ^(b)*^ | | | 16.6±3.5 ^(b)*^ | | | | | |
| R70 | | | 10 | | 0±0 ^(a)^ | | 0±0 ^(a)^ | | 0.8±0.25 ^(a)^ | | 1.85±0.7 ^(a)^ | | | 2.75±0.8 ^(a)^ | | | | | |
|  |  |  | 15 | | 0±0 ^(a)^ | | 0.4±0.16 ^(a)^ | | 1.45±0.4 ^(a)^ | | 2.65±0.8 ^(a)^ | | | 3.35±0.7 ^(a)^ | | | | | |
|  |  |  | 20 | | 0.95±0.4 ^(a)^ | | 2.6±0.4 ^(a)^ | | 7.9±1.8^(b)^ | | 11.0±2.1 ^(b)*^ | | | 11.45±2.4 ^(b)*^ | | | | | |
|  |  |  | 25 | | 2.7±0.8 ^(a)^ | | 7.55±1.6 ^(b)^ | | 10.6±2.4 ^(b)*^ | | 16.3±2.8 ^(c)*^ | | | 21.15±3.3^(c)*^ | | | | | |
| R108 | | | 10 | | 0±0 ^(a)^ | | 0±0^a)^ | | 1.45±0.43^(a)^ | | 2.2±0.4 ^(a)^ | | | 2.7±0.4 ^(a)^ | | | | | |
|  |  |  | 15 | | 0±0^a)^ | | 0.55±0.2 ^(a)^ | | 2.4±0.6 ^(a)^ | | 3.45±0.7 ^(a)^ | | | 4.25±0.95 ^(a)^ | | | | | |
|  |  |  | 20 | | 0.75±0.25 ^(a)^ | | 2.65±0.58 ^(a)^ | | 5.75±0.9 ^(b)^ | | 7.35±1.2 ^(b)*^ | | | 8.55±1.4 ^(b)*^ | | | | | |
|  |  |  | 25 | | 1.9±0.4 ^(a)^ | | 5.6±1.1 ^(b)^ | | 8.9±1.8 ^(c)^ | | 11.6±2.1^(c)*^ | | | 14.35±2.5^(c)*^ | | | | | |
| R118 | | | 10 | | 0±0 ^(a)^ | | 0±0 ^(a)^ | | 0.15±0.05 ^(a)^ | | 0.45±0.15^(a)^ | | | 1.0±0.3 ^(a)^ | | | | | |
|  |  |  | 15 | | 0±0 ^(a)^ | | 0.25±0.1 ^(a)^ | | 0.55±0.15 ^(a)^ | | 1.55±0.35 ^(a)^ | | | 2.35±0.9 ^(a)^ | | | | | |
|  |  |  | 20 | | 1.1±0.25^(a)^ | | 3.2±0.7 ^(b)^ | | 7.15±0.8 ^(b)^ | | 9.2±2.4 ^(b)*^ | | | 11.35±3.4 ^(b)*^ | | | | | |
|  |  |  | 25 | | 2.55±0.65 ^(a)^ | | 6.6±1.4 ^(c)^ | | 8.95±1.6 ^(b)^ | | 10.7±2.5 ^(b)*^ | | | 13.8±4.2 ^(b)*^ | | | | | |
| R137 | | | 10 | | 0±0 ^(a)^ | | 0.15±0.05 ^(a)^ | | 0.85±0.25 ^(a)^ | | 1.9±0.4 ^(a)^ | | | 2.85±0.6 ^(a)^ | | | | | |
|  |  |  | 15 | | 0±0 ^(a)^ | | 0.45±0.2 ^(a)^ | | 1.8±0.5 ^(a)^ | | 3.0±0.5 ^(a)^ | | | 3.55±0.9 ^(a)^ | | | | | |
|  |  |  | 20 | | 1.35±0.5 ^(a)^ | | 3.2±0.8 ^(b)^ | | 6.7±0.9 ^(b)^ | | 11.3±2.1 ^(b)*^ | | | 12.05±2.4 ^(b)*^ | | | | | |
|  |  |  | 25 | | 1.7±0.5 ^(a)^ | | 6.4±1.2 ^(c)^ | | 9.85±1.2 ^(c)^ | | 14.2±2.7 ^(b)*^ | | | 21.85±3.2^(b)*^ | | | | | |
|  |  | Data are presented as mean ± SD. Lower case letters in brackets indicate statistically significant differences (p≤0.05) for each individual strain tested at different temperatures at the same time point (dpi); ANOVA, Tukey’s post hoc test; * indicates statistically significant differences (p≤0.05) between the strains tested at the particular temperature and time point (dpi). | | | | | | | | | | |  | |  | |  |  |  |
| **Comparison between strains** | | | **p-value** | **Comparison between strains** | | **p-value** | | **Comparison between strains** | | **p-value** | | **Comparison between strains** | | | | **p-value** | | | |
| **21 dpi 20°C** | | |  |  | |  | |  | |  | |  | | | |  | | | |
| R23 vs. R32 | | | 0.010 | R53 vs. R49 | | 0.029 | | R23vs. R108 | | 0.041 | |  | | | |  | | | |
| R23 vs. R49 | | | 0.009 | R53 vs. R32 | | 0.029 | | R23 vs. R56 | | 0.048 | |  | | | |  | | | |
| **21 dpi 25°C** | | |  |  | |  | |  | |  | |  | | | |  | | | |
| R53 vs. R32 | | | 0.006 | R70 vs. R32 | | 0.009 | | R1 vs. R32 | | 0.035 | | R53 vs. R56 | | | | 0.041 | | | |
| **28 dpi 20°C** | | |  |  | |  | |  | |  | |  | | | |  | | | |
| R23 vs. R108 | | | <0.001 | R23 vs. R70 | | <0.001 | | R51 vs. R108 | | <0.001 | | R137 vs. R108 | | | | 0.012 | | | |
| R23 vs. R49 | | | <0.001 | R23 vs. R137 | | <0.001 | | R66 vs. R49 | | <0.001 | | R1 vs. R49 | | | | 0.017 | | | |
| R23 vs. R32 | | | <0.001 | R23 vs. R1 | | <0.001 | | R66 vs. R32 | | 0.001 | | R1 vs. R32 | | | | 0.025 | | | |
| R23 vs. R26 | | | <0.001 | R23 vs. R51 | | <0.001 | | R51 vs. R49 | | 0.001 | | R53 vs. R118 | | | | 0.029 | | | |
| R23 vs. R56 | | | <0.001 | R23 vs. R108 | | <0.001 | | R51 vs. R32 | | 0.002 | | R70 vs. R108 | | | | 0.034 | | | |
| R23 vs. R118 | | | <0.001 | R23 vs. R66 | | <0.001 | | R66 vs. R26 | | 0.003 | | R137 vs. R49 | | | | 0.034 | | | |
| R23 vs. R41 | | | <0.001 | R53 vs. R49 | | <0.001 | | R66 vs. R56 | | 0.003 | | R137 vs. R32 | | | | 0.047 | | | |
| R23 vs. R13 | | | <0.001 | R53 vs. R32 | | <0.001 | | R51 vs. R26 | | 0.004 | | R1 vs. R26 | | | | 0.047 | | | |
| R66 vs. R108 | | | <0.001 | R53 vs. R26 | | <0.001 | | R51 vs. R56 | | 0.005 | |  | | | |  | | | |
| R23 vs. R53 | | | <0.001 | R53 vs. R56 | | <0.001 | | R1 vs. R108 | | 0.006 | |  | | | |  | | | |
| **28 dpi 25°C** | | |  |  | |  | |  | |  | |  | | | |  | | | |
| R53 vs. R56 | | | <0.001 | R70 vs. R32 | | <0.001 | | R1 vs. R118 | | <0.001 | | R137 vs. R26 | | | | 0.016 | | | |
| R70 vs. R56 | | | <0.001 | R70 vs. R118 | | <0.001 | | R53 vs. R108 | | 0.002 | | R66 vs. R56 | | | | 0.016 | | | |
| R53 vs. R13 | | | <0.001 | R1 vs. R32 | | <0.001 | | R70 vs. R108 | | 0.002 | | R70 vs. R23 | | | | 0.019 | | | |
| R53 vs. R26 | | | <0.001 | R70 vs. R13 | | <0.001 | | R1 vs. R108 | | 0.003 | | R53 vs. R49 | | | | 0.028 | | | |
| R1 vs. R56 | | | <0.001 | R70 vs. R26 | | <0.001 | | R137 vs. R56 | | 0.002 | | R53 vs. R41 | | | | 0.027 | | | |
| R53 vs. R32 | | | <0.001 | R1 vs. R26 | | <0.001 | | R53 vs. R23 | | 0.003 | | R1 vs. R23 | | | | 0.038 | | | |
| R53 vs. R118 | | | <0.001 | R1 vs. R13 | | <0.001 | | R137 vs. R13 | | 0.017 | | R51 vs. R56 | | | | 0.045 | | | |
| **35 dpi 20°C** | | |  |  | |  | |  | |  | |  | | | |  | | | |
| R23 vs. R32 | | | <0.001 | R53 vs.R32 | | <0.001 | | R51 vs. R26 | | <0.001 | | R23 vs. R66 | | | | 0.002 | | | |
| R23 vs. R26 | | | <0.001 | R53 vs. R26 | | <0.001 | | R66 vs. R13 | | <0.001 | | R51 vs. R56 | | | | 0.003 | | | |
| R23 vs. R108 | | | <0.001 | R23 vs. R51 | | <0.001 | | R23 vs. R53 | | <0.001 | | R137 vs. R32 | | | | 0.005 | | | |
| R23 vs. R49 | | | <0.001 | R23 vs. R41 | | <0.001 | | R51 vs. R108 | | <0.001 | | R53 vs. R118 | | | | 0.011 | | | |
| R23 vs. R56 | | | <0.001 | R23 vs. R137 | | <0.001 | | R53 vs. R56 | | <0.001 | | R53 vs. R70 | | | | 0.015 | | | |
| R23 vs. R13 | | | <0.001 | R66 vs. R32 | | <0.001 | | R1 vs. R32 | | <0.001 | | R41 vs. R32 | | | | 0.021 | | | |
| R23 vs. R118 | | | <0.001 | R66 vs. R26 | | <0.001 | | R51 vs. R49 | | <0.001 | | R53 vs. R41 | | | | 0.030 | | | |
| R23 vs. R70 | | | <0.001 | R66 vs. R108 | | <0.001 | | R53 vs. R108 | | <0.001 | | R137 vs. R26 | | | | 0.030 | | | |
| R66 vs. R70 | | | <0.001 | R23 vs. R1 | | <0.001 | | R66 vs. R56 | | <0.001 | | R70 vs. R32 | | | | 0.041 | | | |
| R1 vs. R26 | | | <0.001 | R53 vs. R49 | | <0.001 | | R66 vs. R137 | | 0.002 | | R137 vs. R108 | | | | 0.048 | | | |
| R66 vs. R118 | | | <0.001 | R51 vs. R32 | | <0.001 | | R53 vs. R13 | | 0.002 | | R1 vs. R56 | | | | 0.047 | | | |
| R1 vs. R108 | | | <0.001 | R66 vs. R41 | | <0.001 | | R1 vs. R49 | | 0.002 | |  | | | |  | | | |
| **35 dpi 25°C** | | |  |  | |  | |  | |  | |  | | | |  | | | |
| R53 vs. R56 | | | <0.001 | R53 vs. R118 | | <0.001 | | R51 vs. R13 | | <0.001 | | R1 vs. R66 | | | | 0.003 | | | |
| R137 vs. R56 | | | <0.001 | R137 vs. R118 | | <0.001 | | R51 vs. R32 | | <0.001 | | R41 vs. R118 | | | | 0.003 | | | |
| R70 vs. R56 | | | <0.001 | R53 vs. R108 | | <0.001 | | R70 vs. R26 | | <0.001 | | R49 vs. R13 | | | | 0.005 | | | |
| R1 vs. R56 | | | <0.001 | R137 vs. R108 | | <0.001 | | R1 vs. R26 | | <0.001 | | R26 vs. R56 | | | | 0.006 | | | |
| R53 vs. R13 | | | <0.001 | R70 vs. R118 | | <0.001 | | R66 vs. R56 | | <0.001 | | R53 vs. R41 | | | | 0.007 | | | |
| R53 vs. R32 | | | <0.001 | R41 vs. R56 | | <0.001 | | R5s vs. R23 | | <0.001 | | R49 vs. R32 | | | | 0.007 | | | |
| R137 vs. R13 | | | <0.001 | R53 vs. R26 | | <0.001 | | R41 vs. R13 | | <0.001 | | R23 vs. R13 | | | | 0.008 | | | |
| R137 vs. R32 | | | <0.001 | R137 vs. R26 | | <0.001 | | R41 vs. R32 | | <0.001 | | R23 vs. R32 | | | | 0.012 | | | |
| R70 vs. R13 | | | <0.001 | R1 vs. R118 | | <0.001 | | R53 vs. R49 | | <0.001 | | R137 vs. R41 | | | | 0.012 | | | |
| R70 vs. R32 | | | <0.001 | R70 vs. R108 | | <0.001 | | R137 vs. R23 | | <0.001 | | R70 vs. R51 | | | | 0.016 | | | |
| R1 vs. R13 | | | <0.001 | R51 vs. R56 | | <0.001 | | R49 vs. R56 | | <0.001 | | R41 vs. R108 | | | | 0.023 | | | |
| R1 vs. R32 | | | <0.001 | R1 vs. R108 | | <0.001 | | R137 vs. R49 | | <0.001 | | R108 vs. R56 | | | | 0.027 | | | |
| R70 vs. R23 | | | <0.001 | R53 vs. R51 | | <0.001 | | R137 vs. R51 | | 0.001 | | R51 vs. R118 | | | | 0.026 | | | |
| R70 vs. R49 | | | <0.001 | R70 vs. R66 | | <0.001 | | R1 vs. R49 | | 0.001 | |  | | | |  | | | |
| R1 vs. R23 | | | <0.001 | R23 vs. R56 | | <0.001 | | R66 vs. R13 | | 0.002 | |  | | | |  | | | |
| R137 vs. R66 | | | <0.001 | R53 vs. R66 | | <0.001 | | R66 vs. R32 | | 0.003 | |  | | | |  | | | |

**Table S5.** Growth kinetics of *R. leguminosarum* sv. *trifolii* strains from the temperate climate population examined in 79CA medium at a wide range of temperatures (10-25°C)

| **Strain** | **Temp.**  **(°C)** | **Optical density (OD_600_) of growth cultures determined after** | | | | | |
| --- | --- | --- | --- | --- | --- | --- | --- |
|  |  | **0** | | **24** | **48** | **72** | **96 hours** |
| 2-2 | 10 | 0.1±0^(a)^ | 0.12±0^(a)^ | | 0.14±0.01^(a)^ | 0.17±0.02^(a)^ | 0.185±0.015^(a)^ |
|  | 15 | 0.1±0^(a)^ | 0.11±0^(a)^ | | 0.23±0.02^(b)*^ | 0.36±0.025^(b)*^ | 0.395±0.035^(b)*^ |
|  | 20 | 0.1±0^(a)^ | 0.15±0.02^(a)*^ | | 0.24±0.025^(b)*^ | 0.38±0.05^(b)*^ | 0.47±0.04^(b)*^ |
|  | 25 | 0.1±0^(a)^ | 0.24±0.02^(b)*^ | | 0.355±0.035^(c)*^ | 0.45±0.04^(b)*^ | 0.46±0.035^(b)*^ |
| 3-1 | 10 | 0.1±0^(a)^ | 0.1±0^(a)^ | | 0.1±0^(a)^ | 0.145±0.005^(a)^ | 0.195±0.005^(a)^ |
|  | 15 | 0.1±0^(a)^ | 0.1±0^(a)^ | | 0.16±0.02^(a)*^ | 0.265±0.035^(b)*^ | 0.37±0.08^(b)*^ |
|  | 20 | 0.1±0^(a)^ | 0.15±0.015^(b)*^ | | 0.28±0.04^(b)*^ | 0.41±0.04^(c)*^ | 0.47±0.03^(b)*^ |
|  | 25 | 0.1±0^(a)^ | 0.225±0.025^(c)*^ | | 0.335±0.045^(b)*^ | 0.57±0.03^(d)*^ | 0.63±0.05^(c)*^ |
| 3-3 | 10 | 0.1±0^(a)^ | 0.1±0^(a)^ | | 0.1±0^(a)^ | 0.125±0.005^(a)^ | 0.165±0.015^(a)^ |
|  | 15 | 0.1±0^(a)^ | 0.11±0^(a)^ | | 0.14±0.02^(a)*^ | 0.195±0.025^(b)*^ | 0.385±0.055^(b)*^ |
|  | 20 | 0.1±0^(a)^ | 0.15±0.025^(b)*^ | | 0.41±0.045^(b)*^ | 0.52±0.05^(c)*^ | 0.53±0.05^(c)*^ |
|  | 25 | 0.1±0^(a)^ | 0.28±0.05^(c)*^ | | 0.36±0.04^(b)*^ | 0.52±0.045^(c)*^ | 0.55±0.03^(c)*^ |
| 4-3 | 10 | 0.1±0^(a)^ | 0.11±0.01^(a)^ | | 0.125±0.005^(a)^ | 0.16±0.02^(a)^ | 0.185±0.005^(a)^ |
|  | 15 | 0.1±0^(a)^ | 0.11±0.01^(a)^ | | 0.135±0.015^(a)*^ | 0.2±0.02^(a)*^ | 0.34±0.02^(b)*^ |
|  | 20 | 0.1±0^(a)^ | 0.24±0.03^(b)*^ | | 0.44±0.06^(b)*^ | 0.52±0.05^(b)*^ | 0.53±0.04^(c)*^ |
|  | 25 | 0.1±0^(a)^ | 0.255±0.025^(b)*^ | | 0.46±0.02^(b)*^ | 0.53±0.02^(b)*^ | 0.545±0.035^(c)*^ |
| 5-8 | 10 | 0.1±0^(a)^ | 0.1±0^(a)^ | | 0.11±0^(a)^ | 0.15±0.01^(a)^ | 0.18±0.02^(a)^ |
|  | 15 | 0.1±0^(a)^ | 0.1±0^(a)^ | | 0.12±0.01^(a)*^ | 0.22±0.05^(a)*^ | 0.29±0.03^(b)*^ |
|  | 20 | 0.1±0^(a)^ | 0.13±0.03^(a)*^ | | 0.31±0.05^(b)*^ | 0.43±0.04^(b)*^ | 0.45±0.03^(c)*^ |
|  | 25 | 0.1±0^(a)^ | 0.23±0.02^(b)*^ | | 0.335±0.045^(b)*^ | 0.46±0.05^(b)*^ | 0.465±0.035^(c)*^ |
| 6-11 | 10 | 0.1±0^(a)^ | 0.11±0.01^(a)^ | | 0.125±0.005^(a)^ | 0.13±0^(a)^ | 0.14±0.01^(a)^ |
|  | 15 | 0.1±0^(a)^ | 0.12±0^(a)^ | | 0.17±0.02^(b)*^ | 0.205±0.025^(b)*^ | 0.255±0.015^(b)*^ |
|  | 20 | 0.1±0^(a)^ | 0.2±0.02^(b)*^ | | 0.39±0.05^(c)*^ | 0.53±0.06^(c)*^ | 0.57±0.06^(c)*^ |
|  | 25 | 0.1±0^(a)^ | 0.38±0.03^(c)*^ | | 0.455±0.035^(c)*^ | 0.58±0.05^(c)*^ | 0.62±0.05^(c)*^ |
| 8-3 | 10 | 0.1±0^(a)^ | 0.105±0.005^(a)^ | | 0.12±0.02^(a)^ | 0.145±0.005^(a)^ | 0.185±0.015^(a)^ |
|  | 15 | 0.1±0^(a)^ | 0.11±0.01^(a)^ | | 0.13±0.02^(a)*^ | 0.185±0.015^(a)*^ | 0.265±0.025^(b)*^ |
|  | 20 | 0.1±0^(a)^ | 0.14±0.025^(ab)*^ | | 0.36±0.04^(b)*^ | 0.45±0.045^(b)*^ | 0.46±0.06^(c)*^ |
|  | 25 | 0.1±0^(a)^ | 0.235±0.025^(b)*^ | | 0.37±0.04^(b)*^ | 0.48±0.03^(b)*^ | 0.49±0.05^(c)*^ |
| 8-11 | 10 | 0.1±0^(a)^ | 0.1±0^(a)^ | | 0.11±0^(a)^ | 0.12±0.01^(a)^ | 0.14±0.015^(a)^ |
|  | 15 | 0.1±0^(a)^ | 0.1±0^(a)^ | | 0.115±0.005^(a)*^ | 0.15±0.01^(a)*^ | 0.19±0.02^(a)*^ |
|  | 20 | 0.1±0^(a)^ | 0.14±0.02^(b)*^ | | 0.34±0.05^(b)*^ | 0.48±0.06^(b)*^ | 0.51±0.05^(b)*^ |
|  | 25 | 0.1±0^(a)^ | 0.3±0.05^(c)*^ | | 0.46±0.03^(b)*^ | 0.59±0.04^(b)*^ | 0.625±0.055^(b)*^ |
| 10-3 | 10 | 0.1±0^(a)^ | 0.1±0^(a)^ | | 0.115±0.005^(a)^ | 0.16±0.015^(a)^ | 0.17±0.02^(a)^ |
|  | 15 | 0.1±0^(a)^ | 0.1±0^(a)^ | | 0.12±0.01^(a)*^ | 0.175±0.015^(a)*^ | 0.215±0.035^(a)*^ |
|  | 20 | 0.1±0^(a)^ | 0.16±0.025^(ab)*^ | | 0.34±0.06^(b)*^ | 0.44±0.05^(b)*^ | 0.46±0.05^(b)*^ |
|  | 25 | 0.1±0^(a)^ | 0.21±0.025^(b)*^ | | 0.365±0.045^(b)*^ | 0.46±0.03^(b)*^ | 0.475±0.025^(b)*^ |
| KW1-9 | 10 | 0.1±0^(a)^ | 0.105±0.005^(a)^ | | 0.11±0^(a)^ | 0.14±0.01^(a)^ | 0.17±0.02^(a)^ |
|  | 15 | 0.1±0^(a)^ | 0.1±0^(a)^ | | 0.115±0.005^(a)*^ | 0.13±0.01^(a)*^ | 0.235±0.025^(b)*^ |
|  | 20 | 0.1±0^(a)^ | 0.16±0.03^(b)*^ | | 0.28±0.04^(b)*^ | 0.36±0.07^(b)*^ | 0.37±0.07^(c)*^ |
|  | 25 | 0.1±0^(a)^ | 0.24±0.04^(c)*^ | | 0.39±0.05^(c)*^ | 0.49±0.03^(b)*^ | 0.5±0.04^(c)*^ |
| KW2-9 | 10 | 0.1±0^(a)^ | 0.11±0^(a)^ | | 0.115±0.005^(a)^ | 0.14±0.01^(a)^ | 0.16±0.02^(a)^ |
|  | 15 | 0.1±0^(a)^ | 0.11±0^(a)^ | | 0.15±0.03^(a)*^ | 0.17±0.02^(a)*^ | 0.215±0.025^(b)*^ |
|  | 20 | 0.1±0^(a)^ | 0.22±0.03^(b)*^ | | 0.38±0.04^(b)*^ | 0.44±0.05^(b)*^ | 0.47±0.03^(c)*^ |
|  | 25 | 0.1±0^(a)^ | 0.25±0.015^(b)*^ | | 0.41±0.04^(b)*^ | 0.48±0.03^(b)*^ | 0.485±0.025^(c)*^ |
| M2 | 10 | 0.1±0^(a)^ | 0.1±0^(a)^ | | 0.11±0^(a)^ | 0.13±0.01^(a)^ | 0.17±0.02^(a)^ |
|  | 15 | 0.1±0^(a)^ | 0.11±0.01^(a)^ | | 0.125±0.015^(a)*^ | 0.175±0.015^(a)*^ | 0.21±0.2^(a)*^ |
|  | 20 | 0.1±0^(a)^ | 0.17±0.03^(b)*^ | | 0.29±0.06^(b)*^ | 0.42±0.03^(b)*^ | 0.44±0.025^(b)*^ |
|  | 25 | 0,1±0^(a)^ | 0.255±0.035^(c)*^ | | 0.34±0.03^(b)*^ | 0.46±0.04^(b)*^ | 0.49±0.045^(b)*^ |
| M14 | 10 | 0.1±0^(a)^ | 0.115±0.005^(a)^ | | 0.12±0^(a)^ | 0.155±0.005^(a)^ | 0.17±0.03^(a)^ |
|  | 15 | 0.1±0^(a)^ | 0.115±0.005^(a)^ | | 0.145±0.015^(a)*^ | 0.175±0.015^(a)*^ | 0.19±0.02^(a)*^ |
|  | 20 | 0.1±0^(a)^ | 0.14±0.02^(a)*^ | | 0.31±0.03^(b)*^ | 0.42±0.05^(b)*^ | 0.45±0.04^(b)*^ |
|  | 25 | 0.1±0^(a)^ | 0.255±0.035^(b)*^ | | 0.4±0.06^(b)*^ | 0.495±0.025^(b)*^ | 0.51±0.04^(b)*^ |
| M16 | 10 | 0.1±0^(a)^ | 0.11±0^(a)^ | | 0.125±0.005^(a)^ | 0.15±0.01^(a)^ | 0.18±0.02^(a)^ |
|  | 15 | 0.1±0^(a)^ | 0.11±0^(a)^ | | 0.135±0.005^(a)*^ | 0.175±0.015^(a)*^ | 0.215±0.025^(a)*^ |
|  | 20 | 0.1±0^(a)^ | 0.18±0.025^(b)*^ | | 0.3±0.05^(b)*^ | 0.4±0.05^(b)*^ | 0.42±0.05^(b)*^ |
|  | 25 | 0.1±0^(a)^ | 0.21±0.03^(b)*^ | | 0.345±0.045^(b)*^ | 0.43±0.03^(b)*^ | 0.445±0.035^(b)*^ |
| M19 | 10 | 0.1±0^(a)^ | 0.115±0.05^(a)^ | | 0.12±0.01^(a)^ | 0.165±0.015^(a)^ | 0.19±0.02^(a)^ |
|  | 15 | 0.1±0^(a)^ | 0.12±0.01^(a)^ | | 0.15±0.015^(a)*^ | 0.18±0.01^(a)*^ | 0.21±0.025^(a)*^ |
|  | 20 | 0.1±0^(a)^ | 0.15±0.02^(a)*^ | | 0.3±0.03^(b)*^ | 0.46±0.07^(b)*^ | 0.49±0.05^(b)*^ |
|  | 25 | 0.1±0^(a)^ | 0.245±0.035^(b)*^ | | 0.38±0.05^(b)*^ | 0.475±0.045^(b)^ | 0.52±0.04^(b)*^ |
| 24.2 | 10 | 0.1±0^(a)^ | 0.11±0^(a)^ | | 0.115±0.005^(a)^ | 0.14±0.015^(a)^ | 0.175±0.015^(a)^ |
|  | 15 | 0.1±0^(a)^ | 0.11±0^(a)^ | | 0.145±0.015^(a)*^ | 0.175±0.015^(a)*^ | 0.225±0.025^(a)*^ |
|  | 20 | 0.1±0^(a)^ | 0.18±0.04^(b)*^ | | 0.37±0.07^(b)8^ | 0.47±0.06^(b)*^ | 0.51±0.03^(b)*^ |
|  | 25 | 0.1±0^(a)^ | 0.27±0.03^(c)*^ | | 0.42±0.05^(b)*^ | 0.49±0.03^(b)*^ | 0.52±0.05^(b)*^ |

Data are presented as mean ± SD. Lower case letters in brackets indicate statistically significant differences (p≤0.05) for each individual strain tested at different temperatures at the same time point (dpi); ANOVA, Tukey’s post hoc test; * indicates statistically significant differences (p≤0.05) between the strains tested at the particular temperature and time point (dpi).

| **Comparison between strains** | **p-value** | **Comparison between strains** | | | **p-value** | **Comparison between strains** | | | **p-value** | **Comparison between strains** | | **p-value** | | |  |
| --- | --- | --- | --- | --- | --- | --- | --- | --- | --- | --- | --- | --- | --- | --- | --- |
| **10°C 24 h** |  |  | | |  |  | | |  |  | |  | | |  |
| 2-2 vs. 3-1 | 0.04 | 3-3 vs. M14 | 0.05 | | | 8-11 vs. M16 | | 0.05 | | 3-1 vs. KW2-9 | | 0.05 | | |  |
| 2-2 vs. 3-3 | 0.04 | 3-3 vs. M16 | 0.05 | | | 8-11 vs. M19 | | 0.05 | | 3-1 vs. M14 | | 0.05 | | |  |
| 2-2 vs. 5-8 | 0.04 | 3-3 vs. M19 | 0.05 | | | 8-11 vs. 24.2 | | 0.05 | | 3-1 vs. M16 | | 0.05 | | |  |
| 2-2 vs. 6-11 | 0.05 | 3-3 vs. 24.2 | 0.05 | | | 10-3 vs. KW1-9 | | 0.05 | | 3-1 vs. M19 | | 0.05 | | |  |
| 2-2 vs. 8-3 | 0.05 | 5-8 vs. 6-11 | 0.05 | | | 10-3 vs. KW2-9 | | 0.05 | | 3-1 vs. 24.2 | | 0.05 | | |  |
| 2-2 vs.8-11 | 0.04 | 5-8 vs. 8-3 | 0.05 | | | 10-3 vs. M14 | | 0.05 | | 3-3 vs. 6-11 | | 0.05 | | |  |
| 2-2 vs. 10-3 | 0.04 | 5-8 vs. KW1-9 | 0.05 | | | 10-3 vs. M16 | | 0.05 | | 3-3 vs. 8-3 | | 0.05 | | |  |
| 2-2 vs. KW1-9 | 0.04 | 5-8 vs. KW2-9 | 0.05 | | | 10-3 vs. M19 | | 0.05 | | 3-3 vs. KW1-9 | | 0.04 | | |  |
| 2-2 vs. KW2-9 | 0.05 | 5-8 vs. M14 | 0.05 | | | 10-3 vs. 24.2 | | 0.05 | | 3-3 vs. KW2-9 | | 0.05 | | |  |
| 2-2 vs. M2 | 0.04 | 5-8 vs. M16 | 0.05 | | | KW1-9 vs. M2 | | 0.05 | | 8-3 vs. 8-11 | | 0.05 | | |  |
| 2-2 vs. M16 | 0.05 | 5-8 vs. M19 | 0.05 | | | KW2-9 vs. M2 | | 0.05 | | 8-3 vs. 10-3 | | 0.05 | | |  |
| 2-2 vs. 24.2 | 0.05 | 5-8 vs. 24.2 | 0.05 | | | M2 vs. M14 | | 0.05 | | 8-3 vs. M2 | | 0.05 | | |  |
| 3-1 vs. 6-11 | 0.05 | 6-11 vs. 8-11 | 0.05 | | | M2 vs. M16 | | 0.05 | | 8-11 vs. KW1-9 | | 0.05 | | |  |
| 3-1 vs. 8-3 | 0.05 | 6-11 vs. 10-3 | 0.05 | | | M2 vs. M19 | | 0.05 | | 8-11 vs. KW2-9 | | 0.05 | | |  |
| 3-1 vs. KW1-9 | 0.04 | 6-11 vs. M2 | 0.05 | | | M2 vs. 24.2 | | 0.05 | | 8-11 vs. M14 | | 0.05 | | |  |
| **10°C 48 h** |  |  |  | | |  |  |  |  |  |  |  |  |  |  |
| 3-1 vs. 5-8 | 0.05 | 2-2 vs. 3-1 | 0.035 | | | 3-3 vs. 6-11 | | 0.04 | | 3-1 vs. M16 | | 0.04 | | |  |
| 3-1 vs. 6-11 | 0.04 | 2-2 vs. 3-3 | 0.035 | | | 3-3 vs. 8-3 | | 0.04 | | 3-1 vs. M19 | | 0.04 | | |  |
| 3-1 vs. 8-3 | 0.04 | 3-1 vs. 4-3 | 0.04 | | | 3-3 vs. 8-11 | | 0.05 | | 3-1 vs. 24.2 | | 0.05 | | |  |
| 3-1 vs. 8-11 | 0.05 | 3-1 vs. KW2-9 | 0.05 | | | 3-3 vs. 10-3 | | 0.05 | | 3-3 vs. 4-3 | | 0.04 | | |  |
| 3-1 vs. 10-3 | 0.05 | 3-1 vs. M2 | 0.05 | | | 3-3 vs. KW1-9 | | 0.05 | | 3-3 vs. 5-8 | | 0.05 | | |  |
| 3-1 vs. KW1-9 | 0.05 | 3-1 vs. M14 | 0.04 | | |  |  |  |  |  |  |  |  |  |  |
| **10°C 72 h** |  |  |  |  |  |  |  |  |  |  |  |  |  |  |  |
| 2-2 vs. 3-3 | 0.05 | 3-3 vs. M14 | 0.035 | | | 6-11 vs. 8-3 | | 0.05 | | 8-11 vs. 10-3 | | 0.04 | | |  |
| 2-2 vs. 6-11 | 0.04 | 3-3 vs. M16 | 0.04 | | | 6-11 vs. 10-3 | | 0.04 | | 8-11 vs. M14 | | 0.04 | | |  |
| 2-2 vs. 8-11 | 0.035 | 3-3 vs. M19 | 0.035 | | | 6-11vs. M14 | | 0.04 | | 8-11 vs. M16 | | 0.05 | | |  |
| 2-2 vs. M2 | 0.04 | 4-3 vs. 6-11 | 0.04 | | | 6-11 vs. M16 | | 0.05 | | 8-11 vs. M19 | | 0.04 | | |  |
| 3-1 vs. 3-3 | 0.04 | 4-3 vs. 8-11 | 0.031 | | | 6-11 vs. M19 | | 0.04 | | 10-3 vs. M2 | | 0.04 | | |  |
| 3-1 vs. 6-11 | 0.05 | 4-3 vs. M2 | 0.04 | | | 8-3 vs. 8-11 | | 0.05 | | M2 vs. M14 | | 0.04 | | |  |
| 3-1 vs. 8-11 | 0.04 | 5-8 vs. 6-11 | 0.05 | | | 8-3 vs. M2 | | 0.05 | | M2 vs. M16 | | 0.05 | | |  |
| 3-1 vs. M2 | 0.05 | 5-8 vs. 8-11 | 0.05 | | | 3-3 vs. 8-3 | | 0.04 | | M2 vs. M19 | | 0.04 | | |  |
| 3-3 vs. 4-3 | 0.035 | 5-8 vs. M2 | 0.05 | | | 3-3 vs. 10-3 | | 0.035 | | 3-3 vs. 5-8 | | 0.04 | | |  |
| **10°C 96 h** |  |  |  |  |  |  |  |  |  |  |  |  |  |  |  |
| 2-2 vs. 3-1 | 0.026 | 2-2 vs. 4-3 | 0.023 | | | 2-2 vs. 6-11 | | 0.013 | | 2-2 vs. 24.2 | | 0.023 | | |  |
| 2-2 vs. 3-3 | 0.02 | 2-2 vs.5-8 | 0.026 | | | 2-2 vs. 8-3 | | 0,026 | | 3-1 vs. 6-11 | | 0.03 | | |  |
| 2-2 vs. 8-11 | 0.013 | 2-2 vs. M14 | 0.026 | | | 5-8 vs. 8-11 | | 0.05 | | 4-3 vs. 8-11 | | 0.035 | | |  |
| 2-2 vs. 10-3 | 0.02 | 2-2 vs. M16 | 0.026 | | | 6-11 vs. 8-3 | | 0.04 | | 5-8 vs. 6-11 | | 0.05 | | |  |
| 2-2 vs. KW1-9 | 0.023 | 2-2 vs.M19 | 0.03 | | | 6-11 vs. 10-3 | | 0.05 | | 6-11 vs. 24.2 | | 0.05 | | |  |
| 2-2 vs. KW2-9 | 0.018 | 3-1 vs. 8-11 | 0.03 | | | 6-11 vs. M16 | | 0.05 | | 8-3 vs. 8-11 | | 0.05 | | |  |
| 2-2 vs. M2 | 0.023 | 4-3 vs. 6-11 | 0.035 | | | 6-11 vs. M19 | | 0.04 | |  |  |  |  |  |  |
| **15°C 48 h** |  |  |  | | |  | |  | |  |  |  |  |  |  |
| 2-2 vs. 3-1 | 0.035 | 2-2 vs. 8-11 | 0.013 | | | 2-2 vs. 24.2 | | 0.026 | | 6-11 vs. KW1-9 | | 0.035 | | |  |
| 2-2 vs. 3-3 | 0.026 | 2-2 vs. 10-3 | 0.018 | | | 3-3 vs. 6-11 | | 0.05 | | 6-11 vs. M2 | | 0.05 | | |  |
| 2-2 vs. 4-3 | 0.023 | 2-2 vs. KW1-9 | 0.015 | | | 4-3 vs. 6-11 | | 0.05 | | 6-11 vs. M16 | | 0.05 | | |  |
| 2-2 vs. 5-8 | 0.018 | 2-2 vs. KW2-9 | 0.026 | | | 5-8 vs. 6-11 | | 0.04 | | 2-2 vs. M14 | | 0.026 | | |  |
| 2-2 vs. 6-11 | 0.035 | 2-2 vs. M2 | 0.02 | | | 6-11 vs. 8-3 | | 0.05 | | 2-2 vs. M16 | | 0.02 | | |  |
| 2-2 vs. 8-3 | 0.02 | 6-11 vs. 10-3 | 0.04 | | | 6-11 vs. 8-11 | | 0.035 | | 2-2 vs. M19 | | 0.03 | | |  |
| **15°C 72 h** |  |  |  | | |  | |  | |  |  |  |  |  |  |
| 2-2 vs. 3-1 | 0.03 | 2-2 vs. 10-3 | 0.011 | | | 2-2 vs. 24.2 | | 0.012 | | 4-3 vs. KW1-9 | | 0.03 | | |  |
| 2-2 vs. 3-3 | 0.012 | 2-2 vs. KW1-9 | 0.0083 | | | 3-1 vs. 8-11 | | 0.023 | | 5-8 vs. 8-11 | | 0.05 | | |  |
| 2-2 vs. 4-3 | 0.012 | 2-2 vs. KW2-9 | 0.012 | | | 3-1 vs. KW1-9 | | 0.015 | | 5-8 vs. KW1-9 | | 0.035 | | |  |
| 2-2 vs. 5-8 | 0.02 | 2-2 vs. M2 | 0.012 | | | 3-3 vs. 8-11 | | 0.05 | | 6-11 vs. 8-11 | | 0.04 | | |  |
| 2-2 vs. 6-11 | 0.012 | 2-2 vs. M14 | 0.012 | | | 3-3 vs. KW1-9 | | 0.035 | | 6-11 vs. KW1-9 | | 0.03 | | |  |
| 2-2 vs. 8-3 | 0.012 | 2-2 vs. M16 | 0.012 | | | 4-3 vs. 8-11 | | 0.04 | | 8-3 vs. 8-11 | | 0.05 | | |  |
| 2-2 vs. 8-11 | 0.009 | 2-2 vs. M19 | 0.012 | | |  |  |  |  |  |  |  |  |  |  |
| **15°C 96 h** |  |  |  | | |  |  |  |  |  |  |  |  |  |  |
| 2-2 vs. 6-11 | 0.015 | 3-1 vs. 10-3 | 0.03 | | | 3-3 vs. M14 | | 0.012 | | 4-3 vs. M2 | | 0.015 | | |  |
| 2-2 vs. 8-3 | 0.018 | 3-1 vs. KW1-9 | 0.035 | | | 3-3 vs. M16 | | 0.013 | | 4-3 vs. M14 | | 0.012 | | |  |
| 2-2 vs. 8-11 | 0.012 | 3-1 vs. KW2-9 | 0.026 | | | 3-3 vs. M19 | | 0.012 | | 4-3 vs. M16 | | 0.015 | | |  |
| 2-2 vs. 10-3 | 0.012 | 3-1 vs. M2 | 0.023 | | | 3-3 vs. 24.2 | | 0.018 | | 4-3 vs. M19 | | 0.013 | | |  |
| 2-2 vs. KW1-9 | 0.013 | 3-1 vs. M14 | 0.018 | | | 4-3 vs. 6-11 | | 0.026 | | 4-3 vs. 24.2 | | 0.02 | | |  |
| 2-2 vs. KW2-9 | 0.012 | 3-1 vs. M16 | 0.023 | | | 4-3 vs. 8-3 | | 0.03 | | 5-8 vs. 6-11 | | 0.035 | | |  |
| 2-2 vs. M2 | 0.012 | 3-1vs. M19 | 0.02 | | | 4-3 vs. 8-11 | | 0.012 | | 5-8 vs. 8-3 | | 0.04 | | |  |
| 2-2 vs. M14 | 0.012 | 3-1 vs. 24.2 | 0.03 | | | 4-3 vs. 10-3 | | 0.02 | | 5-8 vs. 8-11 | | 0.015 | | |  |
| 2-2 vs. M16 | 0.012 | 3-3 vs. 6-11 | 0.023 | | | 4-3 vs. KW1-9 | | 0.023 | | 5-8 vs. 10-3 | | 0.026 | | |  |
| 2-2 vs. M19 | 0.012 | 3-3 vs. 8-3 | 0.026 | | | 4-3 vs. KW2-9 | | 0.018 | | 5-8 vs. KW1-9 | | 0.03 | | |  |
| 2-2 vs. 24.2 | 0.012 | 3-3 vs. 8-11 | 0.012 | | | 3-3 vs. KW1-9 | | 0.023 | | 5-8 vs. KW2-9 | | 0.023 | | |  |
| 3-1 vs. 6-11 | 0.04 | 3-3 vs. 10-3 | 0.018 | | | 3-3 vs. KW2-9 | | 0.015 | | 5-8 vs. M2 | | 0.02 | | |  |
| 3-1 vs. 8-3 | 0.05 | 3-1 vs. 8-11 | 0.018 | | | 3-3 vs. M2 | | 0.013 | | 5-8 vs. M14 | | 0.015 | | |  |
| 5-8 vs. M16 | 0.02 | 6-11 vs. 8-11 | 0.035 | | | 6-11 vs. M19 | | 0.04 | | 8-3 vs. M16 | | 0.026 | | |  |
| 5-8 vs. M19 | 0.018 | 6-11 vs. M14 | 0.035 | | | 8-3 vs. 8-11 | | 0.03 | | 8-3 vs. M19 | | 0.035 | | |  |
| 5-8 vs. 24.2 | 0.026 | 6-11 vs. M16 | 0.05 | | | 8-3 vs. M14 | | 0.035 | |  |  |  |  |  |  |
| **20°C 24 h** |  |  |  |  |  |  |  |  |  |  |  |  |  |  |  |
| 2-2 vs. 4-3 | 0.035 | 4-3 vs. M14 | 0.035 | | | 4-3 vs. 10-3 | | 0.03 | | 4-3 vs. 8-3 | | 0.035 | | |  |
| 3-1 vs. 4-3 | 0.035 | 4-3 vs. M19 | 0.035 | | | 4-3 vs. KW1-9 | | 0.03 | | 4-3 vs. 8-11 | | 0.035 | | |  |
| 3-3 vs. 4-3 | 0.03 | 4-3 vs. 5-8 | 0.035 | | |  |  |  |  |  |  |  |  |  |  |
| **20°C 48 h** |  |  |  |  |  |  |  |  |  |  |  |  |  |  |  |
| 2-2 vs. 3-3 | 0.04 | 3-3 vs. 8-11 | 0.05 | | | 3-3 vs. M16 | | 0.035 | | 4-3 vs. M2 | | 0.05 | | |  |
| 2-2 vs. 4-3 | 0.012 | 3-3 vs. 10-3 | 0.05 | | | 3-3 vs. M19 | | 0.03 | | 4-3 vs. M14 | | 0.03 | | |  |
| 3-1 vs. 3-3 | 0.023 | 3-3 vs.KW1-9 | 0.026 | | | 4-3 vs. 8-3 | | 0.05 | | 4-3 vs. M16 | | 0.035 | | |  |
| 3-1 vs. 4-3 | 0.023 | 3-3 vs.M2 | 0.04 | | | 4-3 vs. 8-11 | | 0.05 | | 4-3 vs. M19 | | 0.04 | | |  |
| 3-3 vs. 5-8 | 0.04 | 3-3 vs. M14 | 0.035 | | | 4-3 vs. 10-3 | | 0.05 | |  |  |  |  |  |  |
| 3-3 vs. 8-3 | 0.026 | 4-3 vs. KW1-9 | 0.023 | | |  |  | | |  |  |  |  |  |  |
| **25°C 24 h** |  |  |  |  |  |  |  |  |  |  |  |  |  |  |  |
| 2-2 vs. 6-11 | 0.018 | 6-11 vs. KW1-9 | 0.026 | | | 6-11 vs. M16 | | 0.015 | | 5-8 vs. 6-11 | | 0.015 | | |  |
| 3-1 vs. 6-11 | 0.018 | 6-11 vs. KW2-9 | 0.026 | | | 6-11 vs. M19 | | 0.018 | | 6-11 vs. 8-3 | | 0.015 | | |  |
| 3-3 vs. 6-11 | 0.05 | 6-11 vs. M2 | 0.026 | | | 6-11 vs. 24.2 | | 0.03 | | 6-11 vs. 10-3 | | 0.013 | | |  |
| 4-3 vs. 6-11 | 0.03 | 6-11 vs. M14 | 0.026 | | |  |  |  |  |  |  |  |  |  |  |
| **25°C 48 h** |  |  |  | | |  |  |  |  |  |  |  |  |  |  |
| 2-2 vs. 4-3 | 0.026 | 3-1 vs. 8-11 | 0.013 | | | 4-3 vs. 5-8 | | 0.03 | | 4-3 vs. M16 | | | | 0.03 |  |
| 2-2 vs. 8-11 | 0.035 | 3-3 vs. 4-3 | 0.04 | | | 4-3 vs. 8-3 | | 0.05 | | 8-11 vs. M2 | | | | 0.04 |  |
| 3-1 vs. 4-3 | 0.03 | 3-3 vs. 8-11 | 0.05 | | | 4-3 vs. M2 | | 0.03 | | 8-11 vs. M16 | | | | 0.04 |  |
| **25°C 96 h** |  |  |  |  |  |  |  |  |  |  |  |  |  |  |  |
| 2-2 vs. 3-1 | 0.023 | 2-2 vs. 4-3 | | 0.03 | | 2-2 vs. 6-11 | 0.013 | | | | 2-2 vs. 8-11 | | 0.013 | | |
| 2-2 vs. 3-3 | 0.023 |  |  |  |  |  |  |  |  |  |  |  |  |  |  |

**Table S6.** Growth kinetics of *R. leguminosarum* sv. *trifolii* strains from the subpolar climate population examined in 79CA medium at a wide range of temperatures (10-25°C)

| **Strain** | **Temp.**  **(°C)** | **Optical density (OD_600_) of growth cultures determined after** | | | | | | | |
| --- | --- | --- | --- | --- | --- | --- | --- | --- | --- |
|  |  | **0** | **24** | | **48** | | **72** | | **96 hours** |
| R1 | 10 | 0.1±0^(a)^ | | 0.11±0^(a)^ | | 0.145±0.005^(a)*^ | | 0.185±0.005^(a)*^ | 0.265±0.025^(a)*^ |
|  | 15 | 0.1±0^(a)^ | | 0.115±0.005^(a)*^ | | 0.155±0.035^(a)^ | | 0.3±0.025^(b)*^ | 0.395±0.35^(b)*^ |
|  | 20 | 0.1±0^(a)^ | | 0.21±0.025^(b)*^ | | 0.45±0.06^(b)*^ | | 0.51±0.06^(c)*^ | 0.52±0.03^(c)*^ |
|  | 25 | 0.1±0^(a)^ | | 0.43±0.03^(c)*^ | | 0.45±0.04^(b)*^ | | 0.49±0.035^(c)*^ | 0.5±0.025^(c)*^ |
| R13 | 10 | 0.1±0^(a)^ | | 0.11±0^(a)^ | | 0.12±0^(a)*^ | | 0.14±0.01^(a)*^ | 0.185±0.015^(a)*^ |
|  | 15 | 0.1±0^(a)^ | | 0.11±0.005^(a)*^ | | 0.22±0.03^(b)*^ | | 0.33±0.03^(b)*^ | 0.475±0.025^(b)*^ |
|  | 20 | 0.1±0^(a)^ | | 0.17±0.02^(b)*^ | | 0.36±0.035^(c)*^ | | 0.47±0.04^(c)*^ | 0.51±0.05^(b)*^ |
|  | 25 | 0.1±0^(a)^ | | 0.23±0.025^(c)*^ | | 0.34±0.03^(c)^ | | 0.43±0.04^(c)*^ | 0.5±0.04^(b)*^ |
| R23 | 10 | 0.1±0^(a)^ | | 0.115±0.005^(a)^ | | 0.145±0.005^(a)*^ | | 0.195±0.025^(a)*^ | 0.335±0.025^(a)*^ |
|  | 15 | 0.1±0^(a)^ | | 0.12±0.01^(a)*^ | | 0.18±0.02^(b)*^ | | 0.235±0.025^(a)*^ | 0.375±0.025^(a)*^ |
|  | 20 | 0.1±0^(a)^ | | 0.21±0.02^(b)*^ | | 0.33±0.02^(c)*^ | | 0.47±0.04^(b)*^ | 0.495±0.03^(b)*^ |
|  | 25 | 0.1±0^(a)^ | | 0.2±0.02^(b)*^ | | 0.29±0.02^(c)*^ | | 0.43±0.03^(b)*^ | 0.475±0.025^(b)*^ |
| R26 | 10 | 0.1±0^(a)^ | | 0.115±0.005^(a)^ | | 0.13±0.005^(a)*^ | | 0.19±0.01^(a)*^ | 0.335±0.025^(a)*^ |
|  | 15 | 0.1±0^(a)^ | | 0.115±0.005^(a)*^ | | 0.14±0.01^(a)*^ | | 0.23±0.03^(a)*^ | 0.48±0.05^(b)*^ |
|  | 20 | 0.1±0^(a)^ | | 0.15±0.02^(b)*^ | | 0.39±0.08^(b)*^ | | 0.42±0.06^(b)*^ | 0.42±0.04^(b)*^ |
|  | 25 | 0.1±0^(a)^ | | 0.255±0.035^(c)*^ | | 0.41±0.04^(b)*^ | | 0.57±0.04^(c)*^ | 0.575±0.05^(c)*^ |
| R32 | 10 | 0.1±0^(a)^ | | 0.12±0^(a)^ | | 0.14±0.01^(a)*^ | | 0.2±0.01^(a)*^ | 0.31±0.01^(a)*^ |
|  | 15 | 0.1±0^(a)^ | | 0.12±0.01^(a)^ | | 0.16±0.03^(a)^ | | 0.23±0.02^(a)*^ | 0.46±0.05^(b)*^ |
|  | 20 | 0.1±0^(a)^ | | 0.15±0.02^(a)*^ | | 0.39±0.04^(b)*^ | | 0.47±0.09^(b)*^ | 0.5±0.07^(b)*^ |
|  | 25 | 0.1±0^(a)^ | | 0.24±0.02^(b)*^ | | 0.44±0.04^(b)*^ | | 0.54±0.04^(b)*^ | 0.58±0.04^(c)*^ |
| R41 | 10 | 0.1±0^(a)^ | | 0.12±0.01^(a)^ | | 0.15±0.01^(a)*^ | | 0.215±0.015^(a)*^ | 0.31±0.03^(a)*^ |
|  | 15 | 0.1±0^(a)^ | | 0.14±0.01^(a)*^ | | 0.16±0.02^(a)*^ | | 0.23±0.02^(a)*^ | 0.345±0.035^(a)*^ |
|  | 20 | 0.1±0^(a)^ | | 0.13±0.03^(a)*^ | | 0.19±0.03^(ab)*^ | | 0.25±0.04^(a)*^ | 0.37±0.04^(a)*^ |
|  | 25 | 0.1±0^(a)^ | | 0.215±0.025^(b)*^ | | 0.3±0.05^(b)^ | | 0.36±0.03^(b)*^ | 0.39±0.03^(ab)*^ |
| R49 | 10 | 0.1±0^(a)^ | | 0.12±0.01^(a)^ | | 0.12±0.01^(a)*^ | | 0.19±0.02^(a)*^ | 0.32±0.03^(a)*^ |
|  | 15 | 0.1±0^(a)^ | | 0.12±0.01^(a)^ | | 0.165±0.005^(a)^ | | 0.23±0.03^(a)*^ | 0.4±0.035^(b)*^ |
|  | 20 | 0.1±0^(a)^ | | 0.14±0.01^(a)*^ | | 0.38±0.04^(b)*^ | | 0.43±0.04^(b)*^ | 0.46±0.04^(b)*^ |
|  | 25 | 0.1±0^(a)^ | | 0.24±0.03^(b)*^ | | 0.4±0.04^(b)*^ | | 0.48±0.04^(b)*^ | 0.485±0.05^(b)*^ |
| R51 | 10 | 0.1±0^(a)^ | | 0.11±0.01^(a)^ | | 0.12±0.01^(a)*^ | | 0.165±0.035^(a)*^ | 0.25±0.03^(a)*^ |
|  | 15 | 0.1±0^(a)^ | | 0.11±0.01^(a)*^ | | 0.145±0.005^(a)^ | | 0.23±0.03^(a)*^ | 0.33±0.03^(b)*^ |
|  | 20 | 0.1±0^(a)^ | | 0.13±0.01^(a)*^ | | 0.32±0.03^(b)*^ | | 0.44±0.04^(b)*^ | 0.45±0.05^(c)*^ |
|  | 25 | 0.1±0^(a)^ | | 0.25±0.02^(b)*^ | | 0.345±0.035^(b)^ | | 0.43±0.03^(b)*^ | 0.44±0.04^(c)*^ |
| R53 | 10 | 0.1±0^(a)^ | | 0.13±0.01^(a)^ | | 0.145±0.025^(a)*^ | | 0.215±0.025^(a)*^ | 0.32±0.03^(a)*^ |
|  | 15 | 0.1±0^(a)^ | | 0.135±0.015^(a)*^ | | 0.16±0.02^(a)^ | | 0.25±0.04^(a)*^ | 0.38±0.04^(a)*^ |
|  | 20 | 0.1±0^(a)^ | | 0.17±0.025^(a)*^ | | 0.33±0.025^(b)*^ | | 0.45±0.03^(b)*^ | 0.47±0.04^(b)*^ |
|  | 25 | 0.1±0^(a)^ | | 0.32±0.03^(b)*^ | | 0.38±0.03^(b)*^ | | 0.44±0.04^(b)*^ | 0.445±0.035^(a)*^ |
| R56 | 10 | 0.1±0^(a)^ | | 0.125±0.005^(a)^ | | 0.13±0.01^(a)*^ | | 0.16±0.02^(a)*^ | 0.265±0.025^(a)*^ |
|  | 15 | 0.1±0^(a)^ | | 0.11±0^(a)*^ | | 0.135±0.005^(a)^ | | 0.25±0.03^(b)*^ | 0.385±0.025^(b)*^ |
|  | 20 | 0.1±0^(a)^ | | 0.15±0.02^(a)*^ | | 0.26±0.03^(b)*^ | | 0.34±0.04^(c)*^ | 0.36±0.03^(b)*^ |
|  | 25 | 0.1±0^(a)^ | | 0.24±0.03^(b)*^ | | 0.35±0.04^(c)^ | | 0.465±0.035^(d)*^ | 0.485±0.035^(c)*^ |
| R66 | 10 | 0.1±0^(a)^ | | 0.12±0^(a)^ | | 0.125±0.005^(a)*^ | | 0.155±0.005^(a)*^ | 0.26±0.03^(a)*^ |
|  | 15 | 0.1±0^(a)^ | | 0.12±0^(a)*^ | | 0.13±0.01^(a)^ | | 0.18±0.03^(a)*^ | 0.28±0.035^(a)*^ |
|  | 20 | 0.1±0^(a)^ | | 0.15±0.015^(a)*^ | | 0.3±0.03^(b)*^ | | 0.38±0.04^(b)*^ | 0.41±0.04^(b)*^ |
|  | 25 | 0.1±0^(a)^ | | 0.25±0.03^(b)*^ | | 0.355±0.025^(b)^ | | 0.515±0.055^(c)*^ | 0.54±0.05^(c)*^ |
| R70 | 10 | 0.1±0^(a)^ | | 0.115±0.005^(a)^ | | 0.12±0^(a)*^ | | 0.135±0.015^(a)*^ | 0.23±0.03^(a)*^ |
|  | 15 | 0.1±0^(a)^ | | 0.11±0.01^(a)*^ | | 0.135±0.015^(a)^ | | 0.18±0.02^(b)*^ | 0.24±0.02^(a)*^ |
|  | 20 | 0.1±0^(a)^ | | 0.13±0.015^(a)*^ | | 0.24±0.015^(b)*^ | | 0.33±0.04^(c)*^ | 0.33±0.03^(b)*^ |
|  | 25 | 0.1±0^(a)^ | | 0.36±0.03^(b)*^ | | 0.42±0.03^(c)*^ | | 0.5±0.05^(d)*^ | 0.5±0.03^(c)*^ |
| R108 | 10 | 0.1±0^(a)^ | | 0.11±0^(a)^ | | 0.12±0.01^(a)*^ | | 0.14±0.01^(a)*^ | 0.275±0.025^(a)*^ |
|  | 15 | 0.1±0^(a)^ | | 0.13±0.02^(a)^ | | 0.165±0.005^(b)^ | | 0.255±0.035^(b)*^ | 0.41±0.04^(b)*^ |
|  | 20 | 0.1±0^(a)^ | | 0.17±0.02^(a)*^ | | 0.42±0.05^(c)*^ | | 0.44±0.03^(c)*^ | 0.43±0.045^(b)*^ |
|  | 25 | 0.1±0^(a)^ | | 0.24±0.03^(b)*^ | | 0.42±0.04^(c)*^ | | 0.57±0.04^(d)*^ | 0.6±0.05^(c)*^ |
| R118 | 10 | 0.1±0^(a)^ | | 0.115±0.005^(a)^ | | 0.12±0.01^(a)*^ | | 0.135±0.005^(a)*^ | 0.19±0.02^(a)*^ |
|  | 15 | 0.1±0^(a)^ | | 0.12±0^(a)*^ | | 0.165±0.015^(a)^ | | 0.235±0.025^(b)*^ | 0.3±0.03^(b)*^ |
|  | 20 | 0.1±0^(a)^ | | 0.17±0.025^(b)*^ | | 0.25±0.03^(b)*^ | | 0.43±0.045^(c)*^ | 0.47±0.05^(c)*^ |
|  | 25 | 0.1±0^(a)^ | | 0.235±0.025^(c)*^ | | 0.41±0.04^(c)*^ | | 0.55±0.04^(d)*^ | 0.65±0.08^(d)*^ |
| R137 | 10 | 0.1±0^(a)^ | | 0.11±0^(a)^ | | 0.125±0.005^(a)*^ | | 0.14±0.01^(a)*^ | 0.23±0.02^(a)*^ |
|  | 15 | 0.1±0^(a)^ | | 0.12±0.02^(a)^ | | 0.17±0.01^(a)^ | | 0.255±0.025^(b)*^ | 0.43±0.03^(b)*^ |
|  | 20 | 0.1±0^(a)^ | | 0.21±0.04^(b)*^ | | 0.35±0.03^(b)*^ | | 0.49±0.06^(c)*^ | 0.51±0.07^(bc)*^ |
|  | 25 | 0.1±0^(a)^ | | 0.23±0.02^(b)*^ | | 0.44±0.05^(c)*^ | | 0.63±0.06^(d)*^ | 0.66±0.07^(c)*^ |

Data are presented as mean ± SD. Lower case letters in brackets indicate statistically significant differences (p≤0.05) for each individual strain tested at different temperatures at the same time point (dpi); ANOVA, Tukey’s post hoc test; * indicates statistically significant differences (p≤0.05) between the strains tested at the particular temperature and time point (dpi).

| **Comparison between strains** | **p-value** | **Comparison between strains** | | | **p-value** | **Comparison between strains** | **p-value** | **Comparison between strains** | | **p-value** |
| --- | --- | --- | --- | --- | --- | --- | --- | --- | --- | --- |
| **10°C 48 h** |  |  |  | | |  |  |  | |  |
| R1 vs. R13 | 0.04 | R13 vs. R32 | 0.04 | | | R32 vs. R49 | 0.04 | R41 vs. R66 | | 0.04 |
| R1 vs. R26 | 0.04 | R13 vs. R41 | 0.04 | | | R32 vs. R51 | 0.04 | R41 vs. R70 | | 0.04 |
| R1 vs. R49 | 0.04 | R23 vs. R26 | 0.04 | | | R32 vs. R56 | 0.05 | R41 vs. R118 | | 0.05 |
| R1 vs. R51 | 0.035 | R23 vs. R49 | 0.04 | | | R32 vs. R66 | 0.05 | R41 vs. R137 | | 0.04 |
| R1 vs. R56 | 0.05 | R23 vs. R51 | 0.05 | | | R32 vs. R70 | 0.05 | R26 vs. R32 | | 0.04 |
| R1 vs. R66 | 0.05 | R23 vs. R56 | 0.05 | | | R32 vs. R118 | 0.05 | R26 vs. R41 | | 0.035 |
| R1 vs. R70 | 0.04 | R23 vs. R66 | 0.04 | | | R32 vs. R137 | 0.05 | R41 vs. R56 | | 0.05 |
| R1 vs. R118 | 0.05 | R23 vs. R70 | 0.04 | | | R41 vs. R49 | 0.035 | R23 vs. R137 | | 0.04 |
| R1 vs. R137 | 0.04 | R23 vs. R118 | 0.04 | | | R41 vs. R51 | 0.05 | R13 vs. R23 | | 0.04 |
| **10°C 72 h** |  |  |  | | |  |  |  | |  |
| R1 vs. R13 | 0.035 | R13 vs. R26 | 0.04 | | | R23 vs. R108 | 0.03 | R41 vs. R70 | | 0.018 |
| R1 vs. R56 | 0.05 | R13 vs. R32 | 0.03 | | | R23 vs. R118 | 0.03 | R41 vs. R108 | | 0.026 |
| R1 vs. R66 | 0.035 | R13 vs. R41 | 0.026 | | | R23 vs. R137 | 0.03 | R41 vs. R118 | | 0.02 |
| R1 vs. R70 | 0.035 | R13 vs. R49 | 0.04 | | | R26 vs. R56 | 0.05 | R41 vs. R137 | | 0.026 |
| R1 vs. R108 | 0.035 | R13 vs. R53 | 0.03 | | | R26 vs. R66 | 0.05 | R49 vs. R56 | | 0.05 |
| R1 vs. R118 | 0.03 | R23 vs. R56 | 0.05 | | | R26 vs. R70 | 0.04 | R49 vs. R66 | | 0.023 |
| R1 vs. R137 | 0.035 | R23 vs. R66 | 0.04 | | | R26 vs. R108 | 0.04 | R49 vs. R70 | | 0.023 |
| R13 vs. R23 | 0.035 | R23 vs. R70 | 0.035 | | | R26 vs. R118 | 0.035 | R53 vs. R70 | | 0.026 |
| R32 vs. R56 | 0.05 | R49 vs. R108 | 0.04 | | | R26 vs. R137 | 0.04 | R53 vs. R108 | | 0.03 |
| R32 vs. R66 | 0.035 | R49 vs. R118 | 0.04 | | | R53 vs. R56 | 0.05 | R53 vs. R118 | | 0.026 |
| R32 vs. R70 | 0.03 | R49 vs. R137 | 0.04 | | | R53 vs. R66 | 0.05 | R53 vs. R137 | | 0.03 |
| R32 vs. R108 | 0.03 | R41 vs. R56 | | 0.04 | | R32 vs. R137 | 0.03 | R32 vs. R118 | | 0.026 |
| R41 vs. R66 | 0.026 |  |  | | |  |  |  | |  |
| **10°C 96 h** |  |  |  | | |  |  | R70 vs. R118 | | 0.05 |
| R1 vs. R13 | 0.03 | R13 vs. R41 | 0.013 | | | R23 vs. R70 | 0.026 | R108 vs. R118 | | 0.026 |
| R1 vs. R23 | 0.04 | R13 vs. R49 | 0.013 | | | R23 vs. R118 | 0.04 | R118 vs. R137 | | 0.05 |
| R1 vs. R32 | 0.05 | R13 vs. R51 | 0.04 | | | R23 vs. R137 | 0.023 | R49 vs. R118 | | 0.015 |
| R1 vs. R41 | 0.05 | R13 vs. R53 | 0.015 | | | R26 vs. R118 | 0.026 | R49 vs. R137 | | 0.026 |
| R1 vs. R49 | 0.05 | R13 vs. R56 | 0.03 | | | R32 vs. R70 | 0.03 | R51 vs. R118 | | 0.05 |
| R1 vs. R53 | 0.05 | R13 vs. R66 | 0.035 | | | R32 vs. R118 | 0.015 | R53 vs. R70 | | 0.035 |
| R1 vs. R118 | 0.035 | R13 vs. R70 | 0.05 | | | R32 vs. R137 | 0.026 | R53 vs. R118 | | 0.018 |
| R13 vs. R23 | 0.012 | R13 vs. R108 | | 0.023 | | R41 vs. R70 | 0.03 | R53 vs. R137 | | 0.03 |
| R13 vs. R26 | 0.023 | R13 vs. R137 | 0.05 | | | R41 vs. R118 | 0.015 | R56 vs. R118 | | 0.05 |
| R13 vs. R32 | 0.013 | R49 vs. R70 | 0.03 | | | R41 vs. R137 | 0.026 | R66 vs. R118 | | 0.05 |
| **15°C 24h** |  |  |  | | |  |  |  | |  |
| R1 vs. R23 | 0.05 | R13 vs. R53 | 0.035 | | | R41 vs. R70 | 0.05 | R53 vs. R70 | | 0.04 |
| R1 vs. R26 | 0.05 | R23 vs. R56 | 0.05 | | | R41 vs. R118 | 0.05 | R53 vs. R118 | | 0.045 |
| R1 vs. R41 | 0.04 | R41 vs. R51 | 0.05 | | | R51 vs. R53 | 0.05 | R56 vs. R66 | | 0.05 |
| R1 vs. R53 | 0.04 | R41 vs. R56 | 0.04 | | | R53 vs. R56 | 0.04 | R56 vs. R118 | | 0.05 |
| R1 vs. R66 | 0.04 | R41 vs. R66 | 0.04 | | | R53 vs. R66 | 0.04 | R13 vs. R41 | | 0.035 |
| R1 vs. R118 | 0.05 |  |  | | |  |  |  | |  |
| **15°C 48h** |  |  |  | | |  |  |  | |  |
| R13 vs. R26 | 0.03 | R23 vs. R26 | 0.04 | | | R13 vs. R41 | 0.04 | R23 vs. R41 | | 0.05 |
| **15°C 72h** |  |  |  | | |  |  |  | |  |
| R1 vs. R66 | 0.03 | R13 vs. R118 | 0.023 | | | R13 vs. R51 | 0.02 | R32 vs. R70 | | 0.05 |
| R1 vs.R70 | 0.026 | R13 vs. R137 | 0.03 | | | R13 vs. R53 | 0.035 | R41 vs. R66 | | 0.05 |
| R13 vs. R23 | 0.023 | R23 vs. R66 | 0.026 | | | R13 vs. R56 | 0.03 | R41 vs. R70 | | 0.05 |
| R13 vs. R26 | 0.023 | R23 vs. R70 | 0.023 | | | R13 vs. R66 | 0.012 | R49 vs. R66 | | 0.05 |
| R13 vs. R32 | 0.02 | R26 vs. R66 | 0.05 | | | R13 vs. R70 | 0.012 | R49 vs. R70 | | 0.05 |
| R13 vs. R41 | 0.02 | R26 vs. R70 | 0.05 | | | R13 vs. R108 | 0.026 | R51 vs. R66 | | 0.05 |
| R13 vs. R49 | 0.02 | R32 vs. R66 | 0.05 | | | R66 vs. R108 | 0.03 | R51 vs. R70 | | 0.05 |
| R56 vs. R66 | 0.05 | R56 vs. R70 | 0.05 | | | R53 vs. R70 | 0.05 | R53 vs. R66 | | 0.05 |
| R66 vs. R118 | 0.05 | R66 vs. R137 | 0.035 | | | R70 vs. R108 | 0.03 | R70 vs. R118 | | 0.05 |
| R70 vs. R137 | 0.035 |  |  | | |  |  |  |  |  |
| **15°C 96h** |  |  |  | | |  |  |  |  |  |
| R1 vs. R66 | 0.05 | R26 vs. R66 | 0.026 | | | R51 vs. R70 | 0.026 | R23 vs. R66 | | 0.015 |
| R1 vs. R70 | 0.05 | R26 vs. R70 | 0.013 | | | R53 vs. R66 | 0.035 | R23 vs. R70 | | 0.01 |
| R13 vs. R23 | 0.026 | R32 vs. R66 | 0.013 | | | R53 vs. R70 | 0.018 | R13 vs. R118 | | 0.012 |
| R13 vs. R41 | 0.02 | R32 vs. R70 | 0.01 | | | R56 vs. R66 | 0.026 | R70 vs. R108 | | 0.012 |
| R13 vs. R51 | 0.013 | R41 vs. R66 | 0.05 | | | R56 vs. R70 | 0.013 | R49 vs. R70 | | 0.012 |
| R13 vs. R66 | 0.01 | R41 vs. R70 | 0.026 | | | R66 vs. R108 | 0.02 | R51 vs. R66 | | 0.05 |
| R13 vs. R70 | 0.008 | R49 vs. R66 | 0.02 | | | R66 vs. R137 | 0.015 | R70 vs. R137 | | 0.01 |
| **20°C 24h** |  |  |  | | |  |  |  | |  |
| R1 vs. R26 | 0.035 | R1 vs. R66 | 0.03 | | | R23 vs. R32 | 0.03 | R23 vs. R108 | | 0.05 |
| R1 vs. R32 | 0.035 | R1 vs. R70 | 0.023 | | | R23 vs. R41 | 0.026 | R23 vs. R118 | | 0.05 |
| R1 vs. R41 | 0.023 | R1 vs. R108 | 0.05 | | | R23 vs. R49 | 0.03 | R41 vs. R53 | | 0.05 |
| R1 vs. R49 | 0.03 | R1 vs. R118 | 0.05 | | | R23 vs. R51 | 0.023 | R41 vs. R108 | | 0.05 |
| R1 vs. R51 | 0.023 | R13 vs. R41 | 0.05 | | | R23 vs. R53 | 0.04 | R41 vs. R137 | | 0.035 |
| R1 vs. R53 | 0.04 | R13 vs. R70 | 0.04 | | | R23 vs. R56 | 0.03 | R53 vs. R70 | | 0.05 |
| R1 vs. R56 | 0.035 | R23 vs. R26 | 0.04 | | | R23 vs. R66 | 0.03 | R23 vs. R70 | | 0.023 |
| R70 vs. R108 |  |  |  | | |  |  |  |  |  |
| **20°C 48h** |  |  |  |  |  |  |  |  |  |  |
| R1 vs. R23 | 0.03 | R23 vs. R32 | 0.04 | | | R26 vs. R118 | 0.013 | R41 vs. R137 | | 0.012 |
| R1 vs. R41 | 0.009 | R23 vs. R41 | 0.015 | | | R32 vs. R41 | 0.011 | R51 vs. R70 | | 0.026 |
| R1 vs. R56 | 0.03 | R23 vs. R49 | 0.05 | | | R32 vs. R56 | 0.02 | R51 vs. R118 | | 0.04 |
| R1 vs. R70 | 0.011 | R23 vs. R70 | | 0.026 | | R32 vs. R70 | 0.013 | R56 vs. R70 | | 0.05 |
| R1 vs. R118 | 0.012 | R23 vs. R108 | 0.03 | | | R32 vs. R118 | 0.013 | R56 vs. R108 | | 0.015 |
| R13 vs. R41 | 0.011 | R23 vs. R118 | 0.04 | | | R41 vs. R49 | 0.011 | R56 vs. R118 | | 0.05 |
| R13 vs. R70 | 0.013 | R26 vs. R41 | 0.009 | | | R41 vs. R51 | 0.015 | R66 vs. R70 | | 0.05 |
| R13 vs. R118 | 0.02 | R26 vs. R56 | 0.013 | | | R41 vs. R53 | 0.015 | R66 vs. R118 | | 0.05 |
| R23 vs. R26 | 0.03 | R26 vs. R70 | 0.01 | | | R41 vs. R56 | 0.035 | R70 vs. R108 | | 0.012 |
| R41 vs. R108 | 0.009 | R108 vs. R118 | 0.013 | | | R41 vs. R66 | 0.026 | R70 vs. R137 | | 0.02 |
| **20°C 72h** |  |  | |  | |  |  |  | |  |
| R1 vs. R41 | <0.001 | R23 vs. R70 | 0.015 | | | R41 vs. R53 | <0.001 | R49 vs. R56 | | 0.04 |
| R1 vs. R56 | 0.013 | R26 vs. R41 | 0.015 | | | R41 vs. R56 | 0.03 | R49 vs. R70 | | 0.035 |
| R1 vs. R70 | 0.012 | R32 vs. R41 | 0.015 | | | R41 vs. R66 | 0.02 | R51 vs. R56 | | 0.03 |
| R13 vs. R41 | <0.001 | R32 vs. R56 | 0.05 | | | R41 vs. R70 | 0.035 | R51 vs. R70 | | 0.026 |
| R13 vs. R56 | 0.011 | R32 vs. R70 | 0.04 | | | R41 vs. R108 | 0.01 | R53 vs. R56 | | 0.023 |
| R13 vs. R70 | 0.015 | R41 vs. R49 | 0.012 | | | R41 vs. R118 | 0.012 | R53 vs. R70 | | 0.02 |
| R23 vs. R41 | <0.001 | R41 vs. R51 | 0.01 | | | R41 vs. R137 | <0.001 | R56 vs. R108 | | 0.05 |
| R23 vs. R56 | 0.018 | R70 vs. R137 | 0.02 | | | R70 vs. R118 | 0.015 | R56 vs. R118 | | 0.04 |
| R70 vs. R108 | 0.026 | R56 vs. R137 | | 0.018 | |  |  |  | |  |
| **20°C 96h** |  |  | |  | |  |  |  | |  |
| R1 vs. R41 | 0.015 | R41 vs. R53 | | 0.03 | | R53 vs. R70 | 0.018 | R23 vs. R70 | | 0.012 |
| R1 vs. R56 | 0.013 | R41 vs. R108 | | 0.05 | | R56 vs. R108 | 0.05 | R32 vs. R41 | | 0.035 |
| R1 vs. R70 | 0.01 | R41 vs. R118 | | 0.05 | | R56 vs. R118 | 0.035 | R32 vs. R56 | | 0.03 |
| R13 vs. R41 | 0.013 | R41 vs. R137 | | 0.03 | | R56 vs. R137 | 0.026 | R32 vs. R70 | | 0.02 |
| R13 vs. R56 | 0.012 | R49 vs. R56 | | 0.03 | | R70 vs. R108 | 0.035 | R41 vs. R49 | | 0.035 |
| R13 vs. R70 | 0.01 | R49 vs. R70 | | 0.02 | | R70 vs. R118 | 0.023 | R41 vs. R51 | | 0.05 |
| R23 vs. R41 | 0.018 | R51 vs. R56 | | 0.05 | | R70 vs. R137 | 0.018 | R53 vs. R56 | | 0.026 |
| R23 vs. R56 | 0.015 | R51 vs. R70 | | 0.03 | |  |  |  |  |  |
| **25°C 24h** |  |  | |  | |  |  |  |  |  |
| R1 vs. R13 | 0.01 | R49 vs. R70 | | 0.02 | | R1 vs. R137 | 0.01 | R1 vs. R53 | | 0.026 |
| R1 vs. R23 | 0.0078 | R51 vs. R70 | | 0.023 | | R13 vs. R70 | 0.02 | R1 vs. R56 | | 0.01 |
| R1 vs. R26 | 0.012 | R56 vs. R70 | | 0.026 | | R23 vs. R70 | 0.015 | R1 vs. R66 | | 0.012 |
| R1 vs. R32 | 0.01 | R66 vs. R70 | | 0.03 | | R26 vs. R70 | 0.04 | R1 vs. R70 | | 0.05 |
| R1 vs. R41 | 0.01 | R70 vs. R108 | | 0.026 | | R32 vs. R70 | 0.018 | R1 vs. R108 | | 0.012 |
| R1 vs. R49 | 0.01 | R70 vs. R118 | | 0.018 | | R41 vs. R70 | 0.015 | R1 vs. R118 | | 0.01 |
| R1 vs. R51 | 0.011 | R70 vs. R137 | | 0.018 | |  |  |  |  |  |
| **25°C 48h** |  |  |  |  |  |  |  |  |  |  |
| R1 vs. R23 | 0.015 | R23 vs. R49 | | 0.03 | | R23 vs. R70 | 0.023 | R23 vs. R118 | | 0.02 |
| R23 vs. R26 | 0.026 | R23 vs. R53 | | 0.035 | | R23 vs. R108 | 0.02 | R23 vs. R137 | | 0.015 |
| R23 vs. R32 | 0.018 |  | |  | |  |  |  | |  |
| **25°C 72h** |  |  |  |  |  |  |  |  |  |  |
| R1 vs. R23 | 0.04 | R23 vs. R66 | | 0.05 | | R26 vs. R41 | 0.013 | R41 vs. R70 | | 0.035 |
| R1 vs. R41 | 0.02 | R23 vs. R70 | | 0.05 | | R32 vs. R41 | 0.013 | R41 vs. R108 | | 0.012 |
| R23 vs. R26 | 0.026 | R23 vs. R108 | | 0.02 | | R41 vs. R49 | 0.035 | R41 vs. R118 | | 0.01 |
| R23 vs. R32 | 0.026 | R23 vs. R118 | | 0.02 | | R41 vs. R56 | 0.05 | R41 vs. R137 | | 0.01 |
| R23 vs. R49 | 0.05 | R23 vs. R137 | | 0.013 | | R41 vs. R66 | 0.04 | R23 vs. R56 | | 0.05 |
| **25°C 96h** |  |  |  |  |  |  |  |  |  |  |
| R1 vs. R41 | 0.026 | R26 vs. R41 | | 0.023 | | R41 vs. R108 | 0.012 | R51 vs. R118 | | 0.012 |
| R1 vs. R118 | 0.015 | R32 vs. R41 | | 0.012 | | R41 vs. R118 | 0.009 | R53 vs. R118 | | 0.01 |
| R13 vs. R41 | 0.04 | R41 vs. R56 | | 0.05 | | R41 vs. R137 | 0.01 | R56 vs. R118 | | 0.015 |
| R13 vs. R118 | 0.01 | R41 vs. R66 | | 0.05 | | R49 vs. R118 | 0.015 | R70 vs. R118 | | 0.023 |
| R23 vs. R41 | 0.05 | R41 vs. R70 | | 0.05 | | R23 vs. R118 | 0.018 | R23 vs. R32 | | 0.018 |
| R23 vs. R66 | 0.05 | R23 vs. R32 | | 0.018 | | R23 vs. R66 | 0.05 |  |  |  |
